# Ratiometric Fluorescent Sensors Illuminate Cellular Magnesium Imbalance in a Model of Acetaminophen-Induced Liver Injury

**DOI:** 10.1021/jacs.3c05704

**Published:** 2023-10-02

**Authors:** Michael Brady, Veronika I. Shchepetkina, Irene González-Recio, María L. Martínez-Chantar, Daniela Buccella

**Affiliations:** † Department of Chemistry, New York University, New York, New York 10003, United States; ‡ Liver Disease Lab, Center for Cooperative Research in Biosciences (CIC bioGUNE), Basque Research and Technology Alliance (BRTA), Bizkaia Technology Park, Building 801A, 48160 Derio, Spain; § Centro de Investigación Biomédica en Red de Enfermedades Hepáticas y Digestivas (CIBERehd), Carlos III National Health Institute, 28029 Madrid, Spain

## Abstract

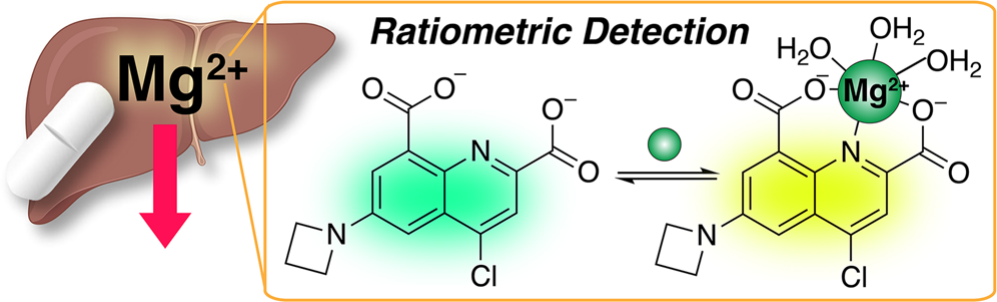

Magnesium(II) plays catalytic, structural, regulatory, and signaling roles in living organisms. Abnormal levels of this metal have been associated with numerous pathologies, including cardiovascular disease, diabetes, metabolic syndrome, immunodeficiency, cancer, and, most recently, liver pathologies affecting humans. The role of Mg^2+^ in the pathophysiology of liver disease, however, has been occluded by concomitant changes in concentration of interfering divalent cations, such as Ca^2+^, which complicates the interpretation of experiments conducted with existing molecular Mg^2+^ indicators. Herein, we introduce a new quinoline-based fluorescent sensor, MagZet1, that displays a shift in its excitation and emission wavelengths, affording ratiometric detection of cellular Mg^2+^ by both fluorescence microscopy and flow cytometry. The new sensor binds the target metal with a submillimolar dissociation constant—well suited for detection of changes in free Mg^2+^ in cells—and displays a 10-fold selectivity against Ca^2+^. Furthermore, the fluorescence ratio is insensitive to changes in pH in the physiological range, providing an overall superior performance over existing indicators. We provide insights into the metal selectivity profile of the new sensor based on computational modeling, and we apply it to shed light on a decrease in cytosolic free Mg^2+^ and altered expression of metal transporters in cellular models of drug-induced liver injury caused by acetaminophen overdose.

## Introduction

Magnesium(II) is an essential metal cation that influences a vast number of cellular processes, ranging from DNA replication and protein synthesis to metabolic enzymatic activity and ion transport.^1,2^ Adequate magnesium intake is crucial for human health,^3^ and abnormal levels of this metal, resulting from dietary deficiency or abnormal absorption/elimination, have been linked with conditions such as cardiovascular disease, diabetes, metabolic syndrome, immunodeficiency, and cancer.^1,4−9^ Magnesium deficiency has also been correlated with the incidence of various liver pathologies^10^ and is exacerbated by alcohol consumption.^11^ Conversely, magnesium supplementation has been shown to reduce mortality from liver disease.^12^

Recently, our team identified the upregulation of cyclin M4 (CNNM4), a protein involved in regulating Mg^2+^ transport,^13,14^ in the development of nonalcoholic steatohepatitis (NASH)^15^ and of drug-induced liver disease (DILI) generated by acetaminophen (paracetamol) overdose.^16^ Significantly, silencing *Cnnm4* restored Mg^2+^ serum levels and reduced steatosis and other hallmarks of both conditions in animal models. A thorough understanding of the mechanism connecting abnormal magnesium levels to these and other liver pathologies, however, is still lacking. The inflammatory response associated with liver damage and disease is thought to involve the activation of several Ca^2+^-dependent pathways and the elevation of Ca^2+^ levels in affected tissues. In fact, it has been proposed that Mg^2+^ deficiency promotes inflammation through the disruption of its natural Ca^2+^ antagonism.^17,18^ Unfortunately, the study of cellular Mg^2+^ in this and other systems in which Ca^2+^ may play a role has been hampered by the scarcity of tools and methods for the dynamic detection of the former cation without interference from the latter. To meet this need, we set out to develop a new fluorescent indicator that could be used for the *selective* detection of Mg^2+^ over Ca^2+^ in live cell imaging applications.

Cation selectivity has been a long-standing challenge in the development of fluorescent indicators for the study of Mg^2+^ in cells. Our thermodynamic analysis of metal binding to APTRA,^19^ a pentadentate metal-binding motif commonly used in Mg^2+^ indicators that exhibits limited selectivity,^20,21^ suggested that decreasing the denticity of carboxylate-based sensors could decrease the affinity for competing divalent cations and improve selectivity for the target Mg^2+^.^22^ The metal binding profile of sensors of the KMG family, based on bidentate metal-binding motifs, illustrates this effect.^23,24^ Bidentate sensors, however, allow the formation of ternary sensor-Mg^2+^-biomolecule complexes that obscure the response to the “free” metal.^25^ Considering this shortcoming and based on the design principles that arose from our thermodynamic analysis, the group of Kikuchi developed a tridentate quinoline-2,8-dicarboxylate (QDC) chelator with excellent Mg^2+^/Ca^2+^ selectivity and low tendency to form ternary complexes.^26,27^ Unfortunately, all sensors built thus far using this chelator display fluorescence quenching, i.e., turn-off response, upon Mg^2+^ coordination as well as strong pH dependence, which limits their utility. The selective detection of free Mg^2+^ in the presence of Ca^2+^ thus remains challenging. Addressing this gap, we report herein a new wavelength *ratiometric* sensor, MagZet1, that affords the selective detection of Mg^2+^ free of Ca^2+^ and pH interference under physiological conditions. We demonstrate the application of the new indicator in both fluorescence microscopy and flow cytometry, shedding light on a decrease in the level of cytosolic free Mg^2+^ correlated with the altered level of expression of CNNM4 in a cellular model of DILI.

## Results and Discussion

### Design and Synthesis of MagZet1, a Mg^2+^-Selective Ratiometric Sensor

Intensiometric, turn-on, and turn-off sensors are the most common types of fluorescent indicators used to visualize metal cations in cellular imaging. The intensity of their fluorescence emission, however, depends on their cellular concentration as much as on the concentration of the target metal itself. Ratiometric indicators showing a wavelength shift are of greater analytical value, as they offer a response that is independent of sensor concentration and minimizes the effect of light source fluctuations and other variables.^28^ To capitalize on the properties of the QDC moiety in the design of a wavelength ratiometric sensor for Mg^2+^, we sought to functionalize the electron-deficient quinoline with an electron-donating amino group to complete a push–pull electron donor–acceptor system. We surmised that coordination of Mg^2+^ to the acceptor moiety would stabilize an internal charge transfer (ICT) excited state, resulting in a red shift in the electronic spectra.^29,30^

To test our design hypothesis, we synthesized a dimethylamino-functionalized sensor, MagDMA (Table 1), as depicted in Scheme S1 (Supporting Information). Treatment of the sensor with increasing concentrations of Mg^2+^ in aqueous buffer (50 mM PIPES, 100 mM KCl, pH 7.0) leads to a hypsochromic shift in absorption from 488 to 430 nm^31^ and a bathochromic shift in emission from 500 to 530 nm (Figure S1). Nonlinear fit of the fluorescence emission data as a function of metal concentration was used to estimate the apparent dissociation constant for Mg^2+^, *K*_d,Mg_ = 0.22 ± 0.01 mM. This apparent affinity is well suited for the detection of physiological concentrations of free Mg^2+^ (0.5–1.2 mM),^32^ while the lower affinity for Ca^2+^ (*K*_d,Ca_ = 2.2 mM, Figure S2) suggests that binding of this cation would not be a source of interference in the cellular milieu. Overall, the spectral shift and favorable preliminary binding data of MagDMA supported the feasibility of the ratiometric detection of biological Mg^2+^ with our design. The low emission quantum yield of the compound (Table 1), however, limited its utility for imaging applications.

**Table 1:**
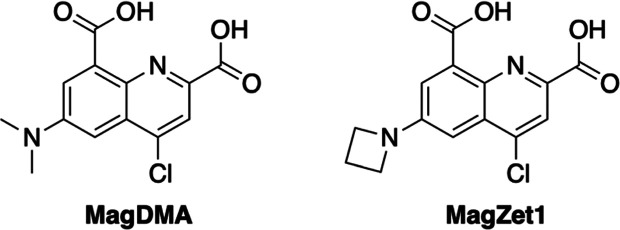
Summary of the Spectroscopic and Metal Binding Properties of Quinoline-Based Fluorescent Indicators for Magnesium, MagDMA, and MagZet1

	Absorption [λ_max_ (nm), ε × 10^3^ M^–1^ cm^–1^]		Fluorescence [λ_max_ (nm), φ (%)^b^]		*K*_d_^′^ (mM)^a^	
	Metal-free	Metal-saturated	Metal-free	Metal-saturated	25 °C	37 °C
**MagDMA**						
Mg^2+^	488, 5.6 ± 0.7	430, 7.3 ± 0.5	500, 6	530, <1	0.22 ± 0.01	ND^c^
Ca^2+^	488	416	500, 6	520, ND	2.2 ± 0.1	ND
**MagZet1**						
Mg^2+^	490, 2.2 ± 0.2	395, 4.9 ± 0.2	500, 40.4 ± 0.9	530, 76 ± 1%	0.14 ± 0.08	0.088 ± 0.003
Ca^2+^	490	383	500, 40.4 ± 0.9	520, ND	1.7 ± 0.5	1.7 ± 0.3
Zn^2+^	490	410	500, 40.4 ± 0.9	545, ND	(2.6 ± 0.6) × 10^–6^	ND

a Values for the dissociation constants were obtained in aqueous buffer (50 mM PIPES, 100 mM KCl, pH 7.0) and represent the average of three independent titrations ± standard deviation.

b Quantum yield values were obtained relative to a quinine sulfate standard in 0.5 M H_2_SO_4_ (φ = 0.54).^33^ Each species was excited at its absorption maximum.

c ND= not determined.

**Scheme 1:**
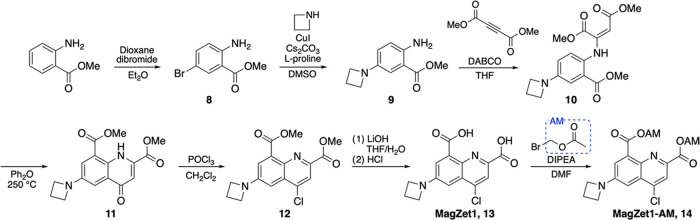
Synthesis of the Sensor MagZet1

Fluorescent dyes featuring dialkylamino substituents often display low emission quantum yields that stem from access to low-energy twisted internal charge transfer (TICT) states, leading to nonradiative relaxation.^34^ Lavis and others have shown that the brightness of many fluorophores can be improved by replacing dimethylamino substituents with azetidine moieties to disfavor the formation of the fully charge-separated TICT state upon photoexcitation.^35,36^ We thus explored this strategy to enhance the brightness of our sensor. MagZet1 (Table 1), an analogue of MagDMA featuring an azetidine moiety at the 6-position of the quinoline, was then synthesized according to Scheme 1. The azetidine moiety was installed through Ullman coupling to methyl anthranilate **8**. The resulting intermediate was then subjected to an aza-Michael addition with dimethyl acetylenedicarboxylate, followed by thermal cyclization to yield oxoquinoline **11**. The sensor in its methyl ester form, compound **12**, was obtained by chlorination with POCl_3_. Quantitative hydrolysis using LiOH in a 1:1 mixture of THF and water followed by neutralization yielded the final MagZet1 sensor in its free acid form.

**Figure 1:**
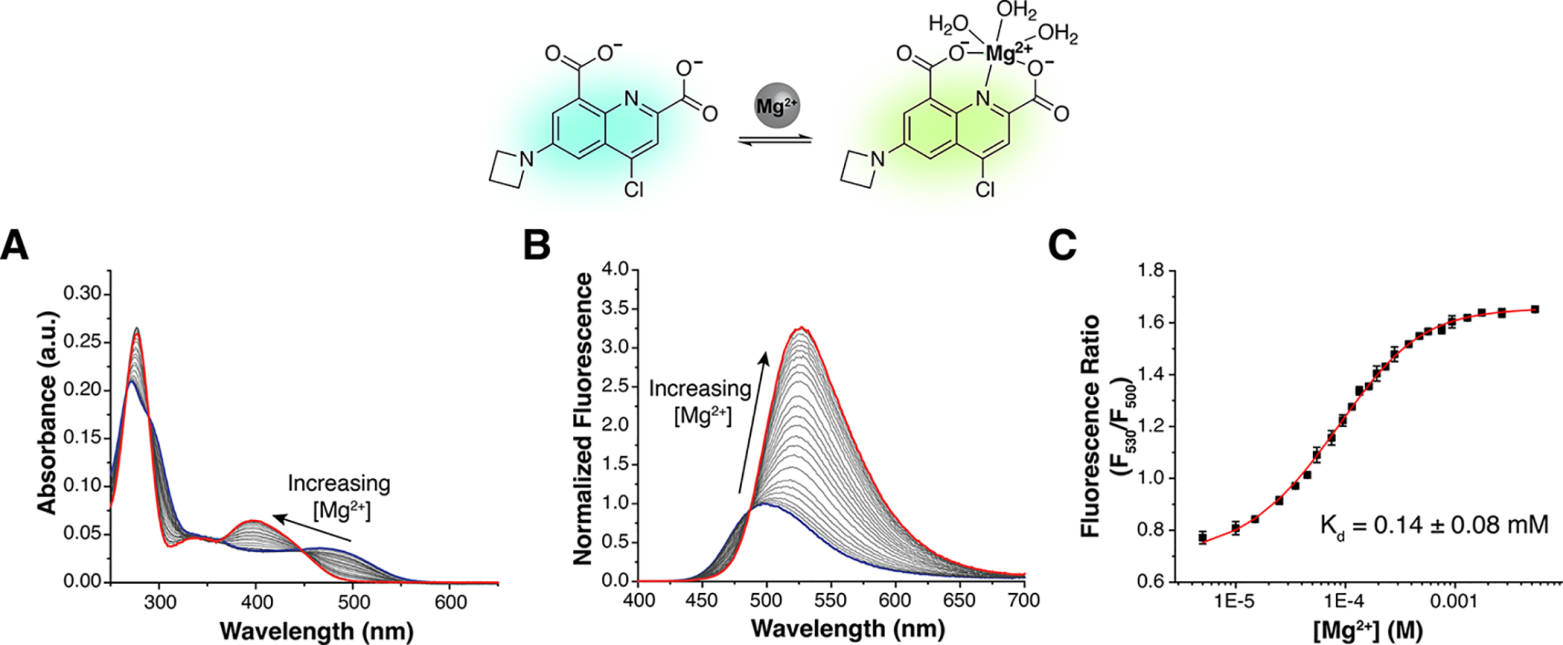
Response of MagZet1 to Mg^2+^. Absorption (A) and fluorescence emission (B) spectra of a 10 μM solution of MagZet1 in aqueous buffer at pH 7.0, treated with increasing concentrations of MgCl_2_. (C) Binding isotherm at 25 °C. Mg^2+^ dissociation constant was determined from nonlinear fit (red curve) of the ratio of fluorescence at 500 and 530 nm (F_530_/F_500_) upon excitation at 390 nm.

The effect of azetidine substitution on the photophysical properties of the resulting sensor was readily apparent. In aqueous buffer at pH 7, MagZet1 shows quantum yields of 40 and 76% in the Mg^2+^-free and -saturated forms, respectively (Table 1), which are desirable for imaging applications. The absorption maximum of the compound shifts from 490 nm in its free form to 395 nm in its Mg^2+^-bound form, whereas the fluorescence emission maximum shifts from 500 to 530 nm upon metal coordination (Figure 1). This spectral shift resembles that of MagDMA and enables both excitation and emission ratiometric detection of the metal. The dissociation constant of MagZet1 at 25 °C (*K*_d_ = 0.14 mM, determined from the emission ratio, F_530_/F_500_ upon excitation at 390 nm) also resembles that of MagDMA and is well-tuned for maximum sensitivity in the physiological range of intracellular free Mg^2+^ concentrations.

**Figure 2:**
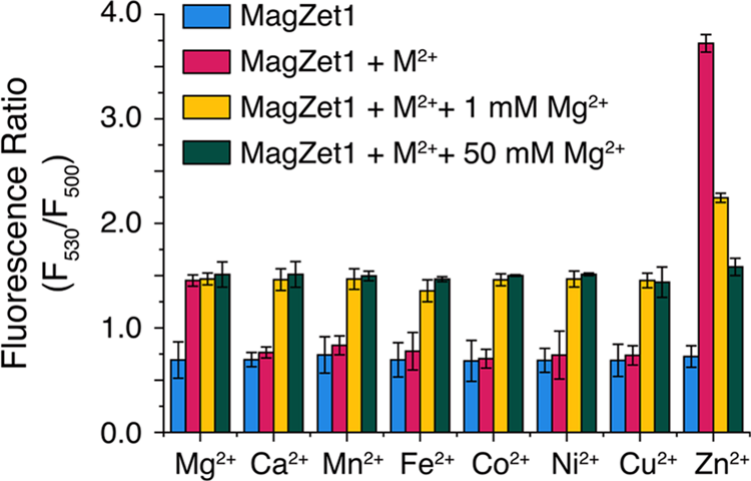
Metal selectivity profile of MagZet1. Fluorescence ratio of 1 μM MagZet1 in aqueous buffer at pH 7.0, 25 °C (blue bars); in the presence of biologically relevant divalent cations (1 mM Mg^2+^, 50 μM Ca^2+^, or 1 μM for other metals, red bars); in the presence of Mg^2+^ (1 mM) and competing cations (yellow bars); or in the presence of saturating Mg^2+^ (50 mM) and competing cations (green bars). Error bars correspond to the standard deviation of triplicate experiments (λ_exc_ = 390 nm).

The optical response of MagZet1 to other biologically relevant divalent cations was also investigated (Figure 2). No significant change in fluorescence ratio was observed in the presence of up to 50 μM Ca^2+^ or equimolar concentrations of first-row *d*-block metals, with the exception of Zn^2+^. An increase in ratio was observed with the latter, but upon closer inspection, we noticed that the Zn^2+^ complex is significantly less emissive than the Mg^2+^ complex and the emission profiles of the two are clearly distinct, which could help discriminate between the metals (Figure S3). Competition experiments conducted with one equivalent of other metals in the presence of 1 mM Mg^2+^ showed no significant interference by the other divalent cations in the detection of physiological levels of Mg^2+^. Some fluorescence quenching was observed in the presence of Co^2+^ and Ni^2+^, suggesting metal binding. The fluorescence is restored, however, in the presence of Mg^2+^.

The thermodynamics of binding of Ca^2+^ and Zn^2+^ to MagZet1 were further investigated (Figures S3–S6). The dissociation constant for the Ca^2+^ complex was determined to be *K*_d,Ca_ = 1.7 mM; thus, typical intracellular concentrations of Ca^2+^ are not expected to interfere with Mg^2+^ detection. The affinity for Zn^2+^, on the other hand, is higher than for either main group metal (*K*_d,Zn_ = 2.6 nM) but still rests outside the range of typical basal concentrations of Zn^2+^ in cells (0.01–0.1 nM).^37^ Accordingly, MagZet1 should not suffer from significant interference from Zn^2+^ in the detection of Mg^2+^ in typical samples. Care should be taken, however, when the sensor is to be employed in samples that are particularly rich in Zn^2+^, such as neurons.^38,39^

### Computational Insight into the Improved Mg^2+^/Ca^2+^ Selectivity

Computational studies were used to gain insight into the cation selectivity of MagZet1. Ground state geometries of the Mg^2+^ and Ca^2+^ complexes were optimized by DFT calculations using the M062X hybrid functional and the 6-311+G(d,p) basis set, as shown to be well suited for push–pull systems and noncovalent interactions.^40^ Input structures were generated from crystal structures of Mg^2+^ complexes of 2-quinoline carboxylic acids.^41^ The coordination spheres of the metals were completed with aqua ligands to achieve typical coordination numbers for Mg^2+^ (six) and Ca^2+^ (eight).

**Figure 3:**
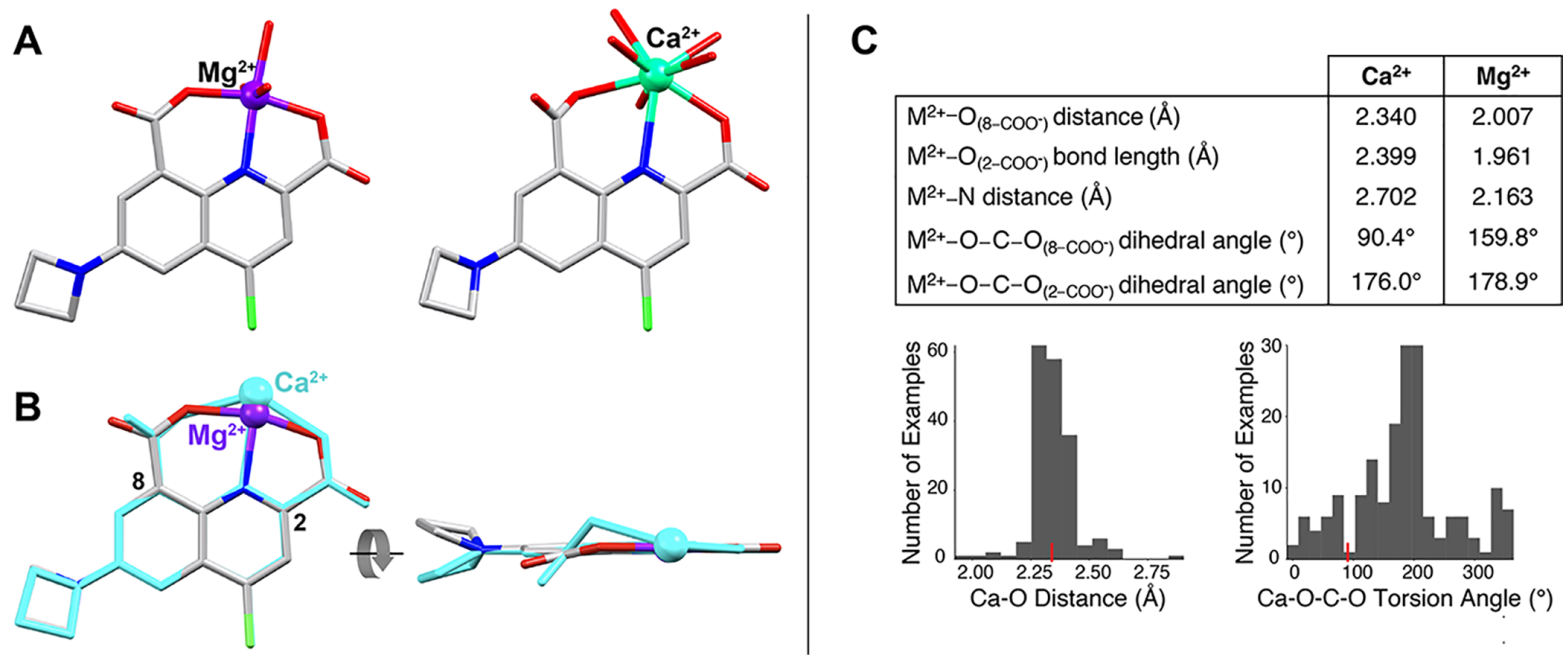
Calculated molecular structures for MagZet1 complexes. (A) Ground state geometry-optimized structures (DFT/M062X) of MagZet1 complexes with Mg^2+^ and Ca^2+^. Hydrogen atoms are omitted for clarity. (B) Overlay of structures, showing the structural distortion of the carboxylate in the 8-position for the Ca^2+^ complex (cyan). Aqua ligands and hydrogen atoms are omitted for clarity. (C) Selected bond lengths and angles on the calculated structures and histograms of Ca–O distances and Ca–O–C–O dihedral angles for all crystallographically characterized monodentate Ca^2+^–acetate complexes in the CSD. Values corresponding to the calculated MagZet1·Ca^2+^ complex are shown in red.

In the optimized geometry for the Mg^2+^ complex, the metal is located approximately in the plane of the sensor (Figure 3). In the Ca^2+^ complex, on the other hand, the larger metal center sits slightly above the plane of the sensor, and the 8-carboxylate group is twisted out of plane by 89.5°. For comparison, most monodentate Ca^2+^ acetate complexes in the Cambridge Crystallographic Database (v 2021.2)^42^ show dihedral angles around 180°, with an average Ca–O distance of 2.3(1) Å (Figure 3).^43,44^ This out-of-plane twist of the carboxylate in [MagZet1·Ca(H_2_O)_5_], not observed in the Mg^2+^ complex, likely weakens the Ca^2+^–carboxylate interaction and overall binding of the metal.

### MagZet1 Detects Mg^2+^ Free from pH Interference

**Figure 4:**
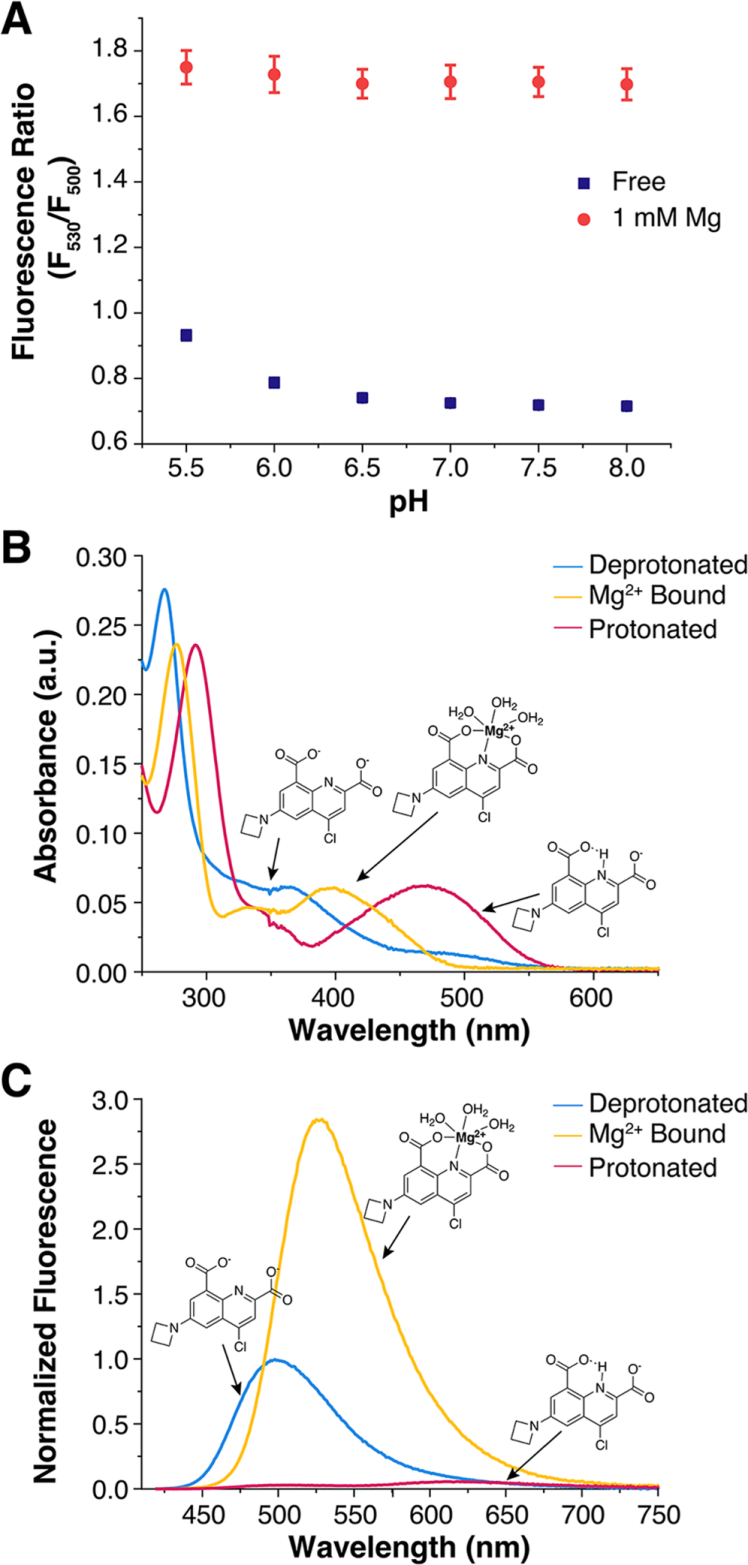
Effect of pH on the fluorescence MagZet1. (A) Fluorescence ratio of a solution of 10 μM MagZet1 as a function of pH in the presence (red circles) and absence (blue squares) of 1 mM MgCl_2_ at 25 °C, λ_exc_ = 390 nm. Values represent the average of three independent measurements with error bars representing the standard deviation. (B) Absorption profile of MagZet1 at 10 μM. (C) Fluorescence emission profile of the same solution.

The response of MagZet1 to changes in pH in the biological range (pH 5–8)^45^ was investigated by both absorption and fluorescence spectroscopy (Figures 4 and S7). The absorption maximum shows a shift from 480 nm in the acidic form to 380 nm in the basic form, with an isosbestic point at 409 nm. The emission spectrum also shows a large blue shift with increasing pH, and the intensity of the emission increases significantly as the sensor is deprotonated. From single-wavelength readings, a p*K*_a_ of 7.1 was determined. The ratio of fluorescence emission intensities at 530 and 500 nm, however, shows almost no pH dependence in the physiologically relevant range of pH= 5.5 to 8 in the presence or absence of metal (Figure 4). This stable response can be attributed to the fact that the protonated form of the sensor is essentially nonemissive and is significantly red-shifted (λ_em_ = 630 nm) compared to both deprotonated and metal-bound forms (Figure 4C). As such, the protonated form can be spectrally resolved from the other two and does not contribute to the F_530_/F_500_ ratio, which effectively becomes dependent on the metal concentration only. The metal selectivity profile also remains relatively unaffected at lower pH values (Figure S8). The overall fluorescence intensity, however, is lower at lower pH values; thus, studies that involve such conditions may require higher concentrations of the sensor, and studies below pH 5 may be hampered by a low signal-to-noise ratio. The isosbestic point for the deprotonated and metal-bound forms of the sensor is 390 nm. This wavelength was used for fluorescence excitation in all photophysical characterization studies conducted in vitro.

### MagZet1 Does Not Suffer from the Formation of Ternary Complexes with Polyphosphates

**Figure 5:**
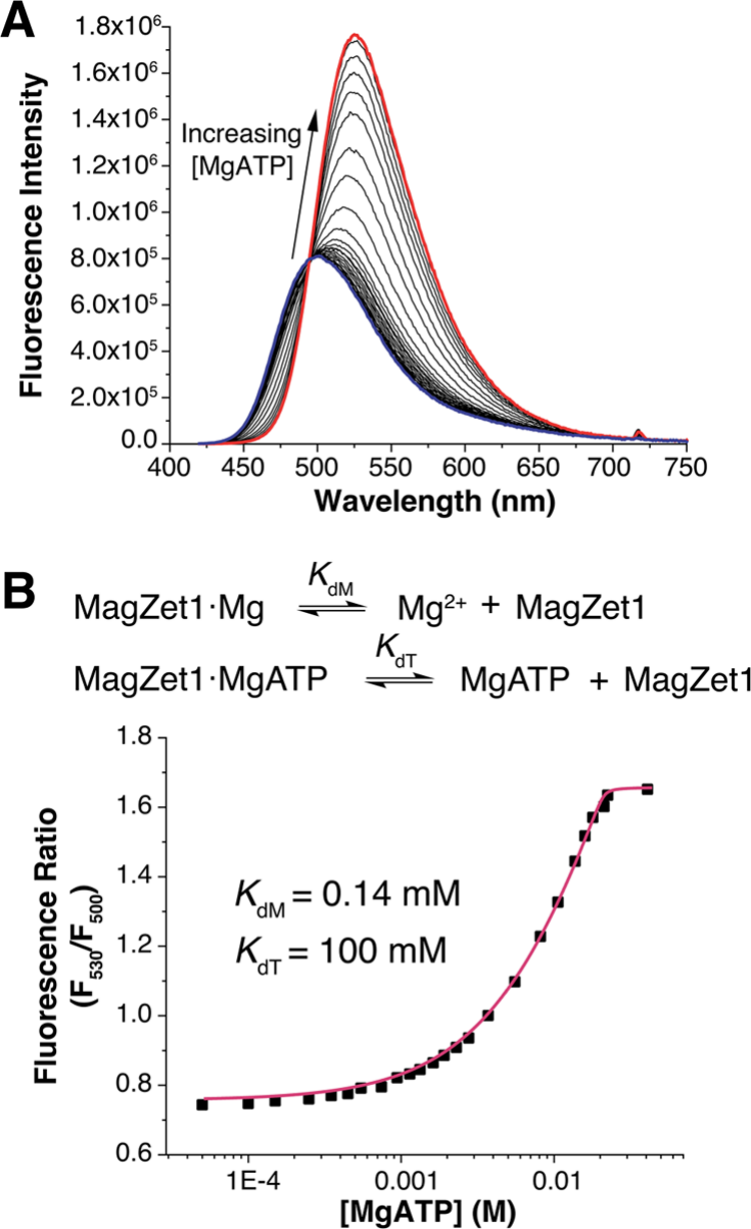
(A) Fluorescence response of a solution of 20 μM MagZet1 to increasing concentrations of MgATP in aqueous buffer at pH 7, 25 °C. λ_exc_ = 390 nm. (B) Binding isotherm at 25 °C. The data could be fitted using a model that includes both binary and ternary complexes, the latter with a weak binding constant.

An established shortcoming of low-denticity sensors and chelators is their propensity to form biomolecule·Mg^2+^·sensor ternary complexes,^25^ which may hamper the study of free Mg^2+^ in biological matrices. Therefore, the ability of MagZet1 to form ternary complexes with MgATP was tested. The addition of high concentrations of MgATP, prepared in situ from a one-to-one mixture of Mg^2+^ and ATP, led to a gradual increase in wavelength and intensity of the fluorescence emission (Figure 5). The fluorescence ratio as a function of concentration was analyzed by using a model that incorporated the formation of both binary [MagZet1·Mg^2+^] (from competition with ATP) and ternary [MagZet1·Mg^2+^·ATP] complexes (Figures 5 and S9). Using this model and a dissociation constant of 50 μM for MgATP,^46^ we determined a dissociation constant of 100 mM for the ternary complex. This value far exceeds the total concentration of Mg^2+^ in the cell (17–20 mM, including both free and biomolecule-bound metal);^7^ thus, MagZet1 could be used to monitor changes in intracellular free Mg^2+^ with little interference from the bound forms.

We also investigated the effect of various amino acids and metabolites that may bind the metal or interact with the fluorescent indicator, including histidine, glutamate, aspartate, cysteine, glutathione, citrate, pyruvate, and malate. The presence of these species at physiological levels neither altered the fluorescence output of the sensors nor its response to Mg^2+^ (Figure S10).

### Visualizing Cytosolic Mg^2+^ Content in Live Cells by Microscopy and Flow Cytometry

**Figure 6:**
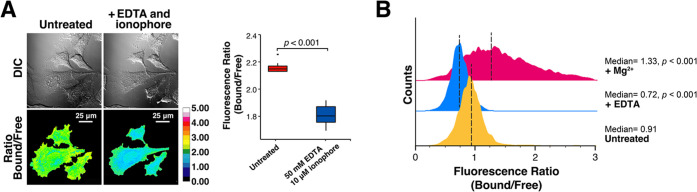
MagZet1 can be used to detect Mg^2+^ in live cells. (A) Fluorescence images of HeLa cells stained with 5 μM MagZet1-AM before and after treatment with ionophore 4-Br-A-23187 and EDTA. Box plot shows the change in the fluorescence ratio from a decrease in intracellular Mg^2+^ concentration. *t* test, *n* = 10. (B) Flow cytometry histograms of fluorescence ratio of HeLa cells stained with MagZet1-AM in the presence of 50 mM Mg^2+^ and 10 μM ionophore 4-Br-A-23187 (pink) or coloaded with 1 mM EDTA-AM (blue) vs untreated controls (yellow). Dashed lines mark the median fluorescence ratio. *p*-values were calculated from χ^2^ values corresponding to comparisons of each population to the vehicle-treated control.

Given the excellent properties shown in the cuvette, the ability of MagZet1 to detect Mg^2+^ in live cells was investigated. The sensor was converted into an acetoxymethyl ester form, MagZet1-AM (Scheme 1), to enable passive loading into cells.^47^ HeLa cells incubated with 5 μM MagZet1-AM, followed by a wash and 30 min de-esterification period, showed bright diffuse cytosolic staining, indicating even distribution of the sensor (Figure 6). To verify that the sensor could detect changes in Mg^2+^ concentration, cytosolic Mg^2+^ was lowered by treatment with excess extracellular EDTA in the presence of nonfluorescent ionophore, 4-Br-A-23187, to equilibrate the Mg^2+^ concentration across membranes.^48−50^ Within several minutes, a significant decrease in the fluorescence ratio was observed, confirming that the sensor was indeed sensitive to changes in intracellular Mg^2+^ content (Figure 6). Experiments conducted with either BAPTA-AM, a Ca^2+^-specific chelator, or tris-picolylamine (TPA), a rapid and low-toxicity Zn^2+^ chelator,^51^ did not result in significant changes in the fluorescence ratio, thus ruling out the detection of either of these metals by MagZet1 (Figure S11).

We then sought to investigate the detection of intracellular Mg^2+^ by flow cytometry, taking advantage of the shift in the emission wavelength elicited by metal binding to MagZet1. Flow cytometry is a powerful technique that enables the rapid analysis of large populations of cells, providing robust measurements and statistics. This technique, however, has not been widely used for the study of Mg^2+^ due to the lack of suitable probes. Mag-Indo-1, an analogue of the Ca^2+^ sensor Indo-1 suitable for such applications,^52^ suffers from significant interference from competing divalent cations and interactions with proteins,^53,54^ photobleaching, and is no longer available from most commercial sources. As such, there is a need for new emission ratiometric sensors for free Mg^2+^ compatible with the technique.

HeLa cells stained with 5 μM MagZet1-AM were analyzed using a 405 nm excitation laser and the combination of 450/50 bandpass (for the metal-free sensor) and 530/30 bandpass (for the metal-bound) filters. Treatment with the sensor did not have a negative impact on cell shape or granularity (Figure S12), typically associated with cell toxicity. Suspensions of MagZet1-stained HeLa cells in the absence and presence of excess extracellular Mg^2+^ and metal ionophore 4-Br-A-23817, or coloaded with EDTA-AM, were then analyzed. Histograms showed significantly different fluorescence ratios arising from the sample treated with excess Mg^2+^ (pink, Figure 6) and the sample coloaded with EDTA-AM (blue, Figure 6), compared to the untreated stained cells (yellow, Figure 6). These results confirm that MagZet1 can be used to detect changes in intracellular Mg^2+^ by both microscopy and flow cytometry.

### Liver Cells Treated with Acetaminophen Show Decreased Intracellular Mg^2+^ Linked to Overexpression of CNNM4

Acetaminophen (APAP) is a common drug used to treat mild to moderate pain. This drug, however, is responsible for more than 50% of overdose-related acute liver failure cases in the United States.^55^ APAP hepatoxicity occurs through the formation of a reactive metabolite, *N-*acetyl-*p*-benzoquinoneimine (NAPQI), which depletes glutathione and leads to Ca^2+^ redistribution, mitochondrial dysfunction, and ER stress in hepatocytes.^55−57^ CNNM4, a protein involved in regulating Mg^2+^ transport,^13,14^ was found to be markedly upregulated in hepatocytes from patients suffering DILI generated by APAP overdose, whereas its silencing resulted in restoration of Mg^2+^ serum levels and amelioration of various hallmarks of the disease in animal models.^16^ With a sensor capable of the selective detection of Mg^2+^ against Ca^2+^, we set out to investigate changes in Mg^2+^ in a cellular model of DILI by flow cytometry.

**Figure 7:**
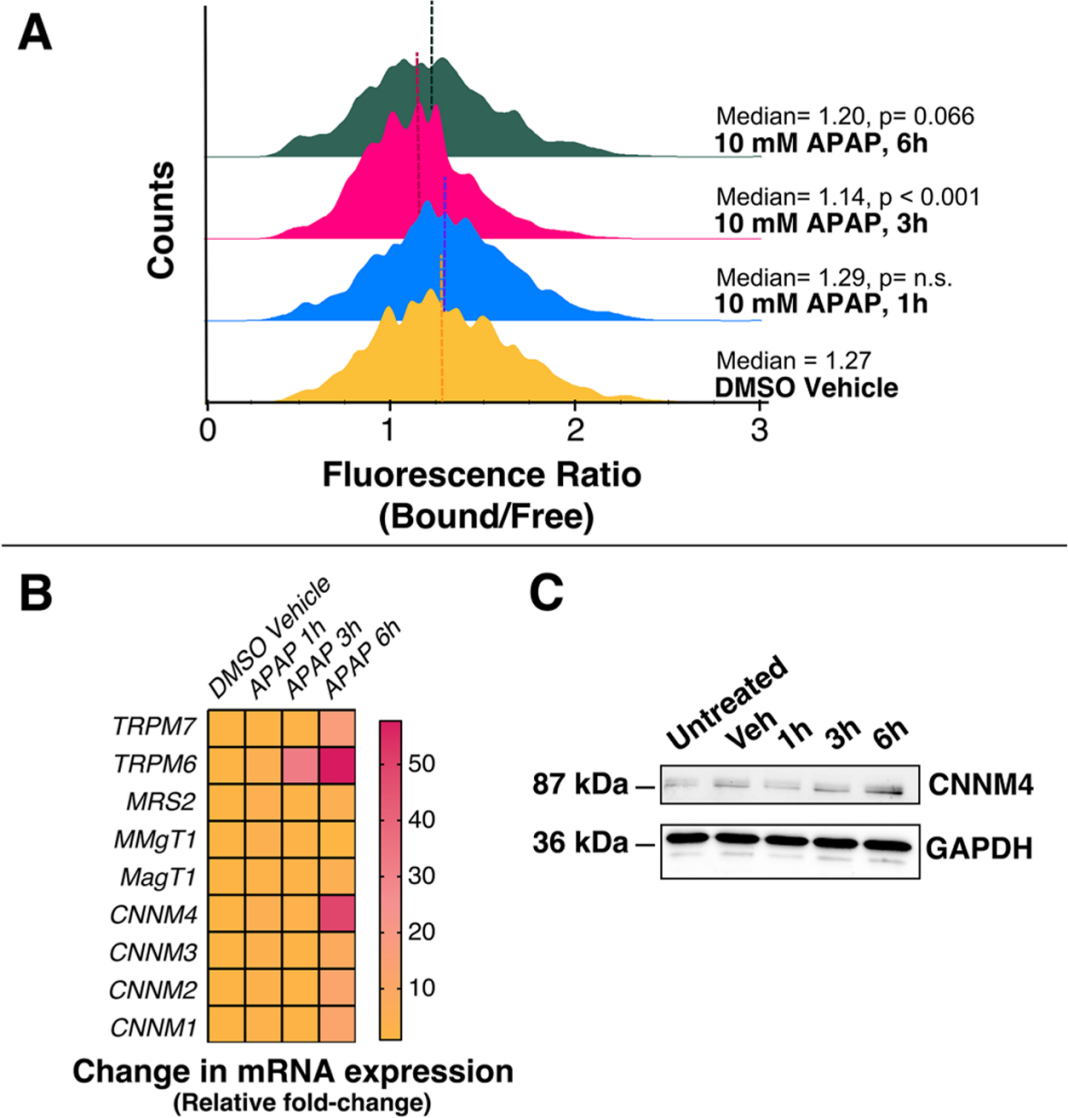
Changes in Mg^2+^ levels and transporters in liver cells treated with acetaminophen, APAP. (A) Flow cytometry histograms of the MagZet1 fluorescence ratio in THLE-2 cells treated with DMSO vehicle (yellow) or 10 mM APAP for 1 (blue), 3 (red), or 6 h (green). Dashed lines represent the median fluorescence ratio. *p*-values were calculated from χ^2^ values corresponding to comparison of the corresponding population to the vehicle-treated control. (B) Changes in mRNA levels of genes encoding Mg^2+^ transporters *TRPM7, TRPM6, MRS2, MMgT1, MagT1*, and *CNNM1–4* in THLE-2 cells under exposure to 10 mM APAP for 1, 3, or 6 h vs DMSO vehicle. (C) Protein levels of CNNM4 (Western blot) in THLE-2 cells under exposure to 10 mM APAP for 1, 3, or 6 h vs DMSO vehicle control group.

Transfected human liver epithelial (THLE-2) cells express the levels of phase I and phase II drug-metabolizing enzymes and transporters comparable to primary hepatocytes^58,59^ and display distinct characteristics of DILI within several hours of treatment with sublethal concentrations (5–10 mM) of APAP.^16^ Fluorescence microscopy revealed diffuse cytosolic staining in THLE-2 cells treated with 5 μM MagZet1-AM for 30 min at room temperature, followed by an additional 30 min de-esterification period at room temperature (Figure S13). We thus used similar sensor loading conditions in the preparation of samples for flow cytometry. THLE-2 cells treated with 10 mM APAP displayed a decrease in the fluorescence ratio after just 3 h of exposure to the drug (Figures 7 and S14–S15). Analysis of the expression of various proteins involved in magnesium transport revealed an upregulation of *CNNM4*, consistent with prior observations in primary hepatocytes from DILI patients and animal models.^16^ An upregulation of *TRPM6* was also detected by RT-qPCR (Figure 7), although this had not been observed in patient samples. Of note, TRPM6 is a channel kinase with multiple cation conductance^60,61^ that is mainly expressed in the kidney and colon^62^ and whose mutations are linked to hypomagnesemia with secondary hypocalcemia.^63^ The excellent Mg^2+^/Ca^2+^ selectivity afforded by MagZet1 is key to the unambiguous analysis of cellular Mg^2+^ in this and other systems in which Ca^2+^ levels may be altered as well.

## Conclusions

The study of Mg^2+^ in Ca^2+^-rich environments by fluorescence techniques requires tools that selectively detect Mg^2+^. While much effort has been put into achieving this goal, current sensors still lack on multiple fronts.^26,27,64^ Addressing this gap, we developed MagZet1, a ratiometric sensor based on a 2,8-quinoline dicarboxylate chelator, which displays a 10-fold selectivity for Mg^2+^ over Ca^2+^ and a dissociation constant well suited for the detection of typical cellular concentrations of the metal. Upon metal binding, MagZet1 displays a red shift in fluorescence emission, making it one of the few emission ratiometric sensors available for studying Mg^2+^. Significantly, the fluorescence emission ratio is solely dependent on the concentration of free Mg^2+^ and does not suffer from significant pH interference in the biological range. Moreover, the sensor has a low propensity to form ternary complexes with polyphosphates at typical biological concentrations, making MagZet1 an excellent tool for the study of cellular free Mg^2+^.

The emission ratiometric response of MagZet1 expands the possibilities beyond conventional microscopy techniques to applications in flow cytometry, a powerful technique that has thus far been underutilized in the study of Mg^2+^ homeostasis. Flow cytometry allows the robust analysis of large numbers of cells, providing greater insight into population heterogeneity and the detection of small but significant changes. With a suitable sensor in hand, we employed flow cytometric analysis to shed light on a decrease in the level of cytosolic Mg^2+^ in a cellular model of APAP-induced liver injury. The changes in metal levels correlate with upregulation of CNNM4 in the cells, previously observed in liver biopsies from DILI patients,^16^ and are consistent with CNNM4 playing a role in promoting metal extrusion from the liver. The selectivity for Mg^2+^ over Ca^2+^ displayed by the sensor is imperative for the study of this system, in which changes in ER Ca^2+^-releasing capacity and ER stress reveal alterations in Ca^2+^ homeostasis at play. We anticipate that the new sensor and methodology reported herein will likewise open the door to the study of Mg^2+^ in other systems, thus far largely inaccessible, in which Ca^2+^ levels are high or may be altered. As such, it may help to shed light on the intricate interplay between the two cations that, for the lack of better tools, had only been studied from a Ca^2+^-centric perspective.

## Supporting Information Available

Supporting figures and tables, experimental procedures, coordinates of optimized geometries of metal complexes from DFT calculations, and spectroscopic characterization data for new compounds (PDF)
