# Supplementary material for: Ratiometric Fluorescent Sensors Illuminate Cellular Magnesium Imbalance in a Model of Acetaminophen-Induced Liver Injury
Source: J Am Chem Soc. 2023 Oct 2;145(40):21841–50. doi: 10.1021/jacs.3c05704 (PMC10571084; doi:10.1021/jacs.3c05704)
Supplement: Supplementary file 1 — ja3c05704_si_001.pdf [file ja3c05704_si_001.pdf]

Supporting Information to

Ratiometric fluorescent sensors illuminate cellular magnesium imbalance in model of acetaminophen-induced liver injury

Michael Brady[1], Veronika I. Shchepetkina,[1] Irene González-Recio[2], María L. Martínez-Chantar[2,3]*, and Daniela Buccella[1]*

Table of Contents

1 Supporting Figures and Tables 2

2 Experimental Details 10

2.1 Synthesis of new compounds 10

2.1.1 General synthetic details 10

2.1.2 Synthesis of MagDMA 10

2.1.3 Synthesis of MagZet1 15

2.2 Spectroscopic Studies 20

2.2.1 General spectroscopic details 20

2.2.2 Spectroscopic Measurements 21

2.3 Computational details 26

2.4 Cell culture 29

2.5 Imaging 29

2.5.1 General imaging details 29

2.5.2 Imaging free Mg^{2+} in HeLa cells with MagZet1AM 30

2.5.3 Control experiments to rule out detection of Ca^{2+} or Zn^{2+} 30

2.5.4 Imaging free Mg^{2+} in paracetamol-treated THLE-2 cells with MagZet1AM 31

2.6 Flow cytometry 31

2.6.1 Statistical analysis 32

2.7 Analysis of transporter expression in paracetamol-treated THLE-2 cells 33

2.7.1 RNA isolation and first strand cDNA generation 33

2.7.2 Quantitative Real-Time Polymerase Chain Reaction (RT-qPCR) 33

2.7.3 Protein isolation and Western blotting 34

3 Spectroscopic data for new compounds 35

4 References 54

1 Supporting Figures and Tables

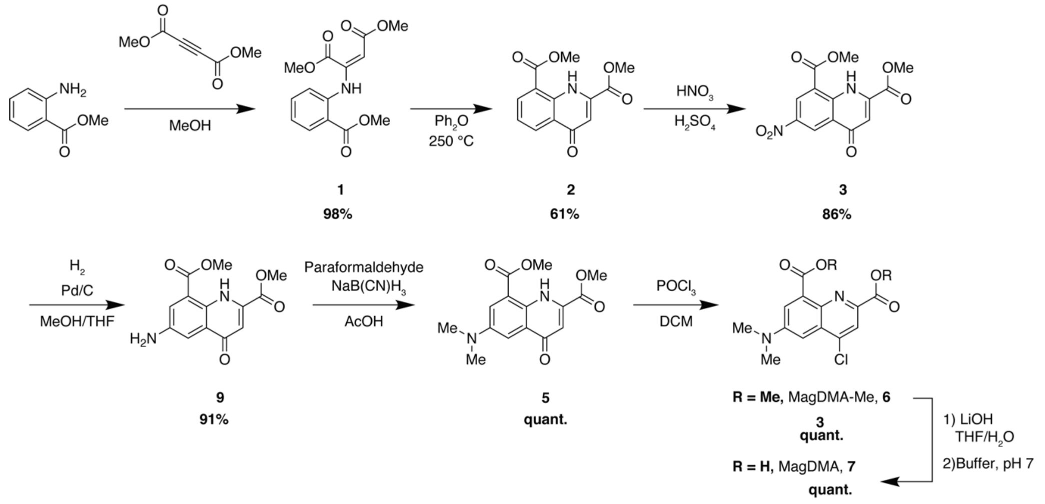

Scheme S1. Synthesis of MagDMA.

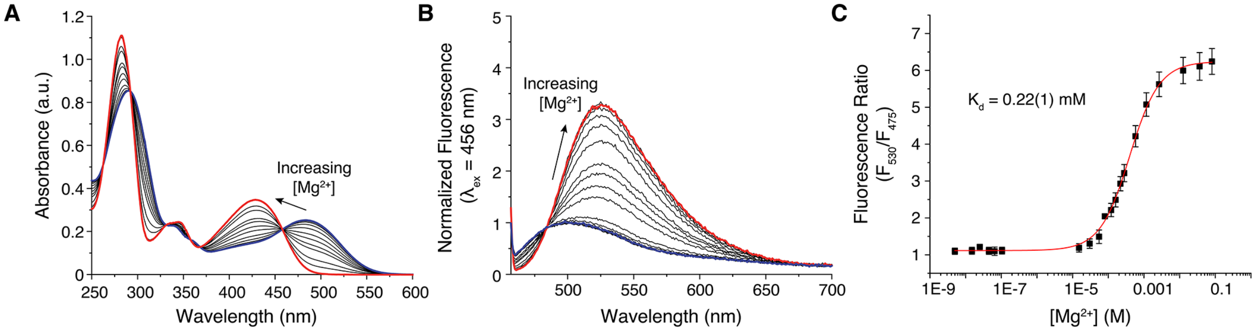

Figure S1. Absorption (A) and fluorescence emission (B) spectra of 25 µM MagDMA tretated with increasing concentrations of $MgCl_2$ in aqueous buffer (50 mM PIPES, 100 mM KCl, pH = 7.0) at 25 °C. (C) Non-linear fit of the fluorescence isotherm, λ_{exc} = 456 nm.

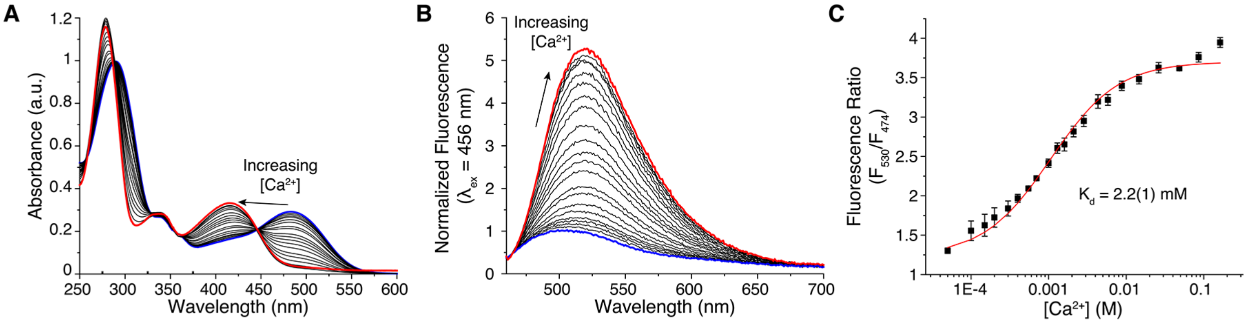

Figure S2. Absorption (A) and fluorescence emission (B) spectra of 25 μM MagDMA treated with increasing concentrations of $CaCl_2$ in aqueous buffer (50 mM PIPES, 100 mM KCl, pH = 7.0) at 25 °C. (C) Non-linear fit of the fluorescence isotherm, λ_{exc} = 456 nm.

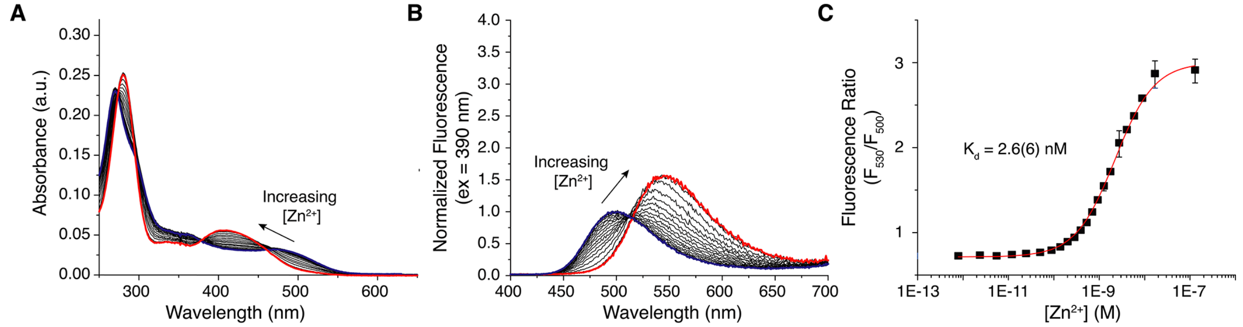

Figure S3. Absorption (A) and fluorescence (B) spectra of 10 μM MagZet1 in the presence of increasing concentrations of $ZnCl_2$ in aqueous buffer (50 mM PIPES, 100 mM KCl, pH = 7.0) at 25 °C. (C) Non-linear fit of the fluorescence isotherm, λ_{exc} = 390 nm.

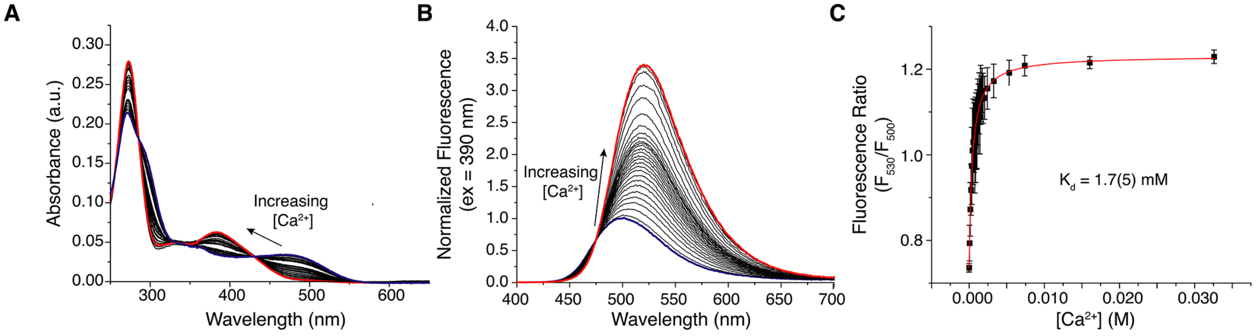

Figure S4. Absorption (A) and fluorescence (B) spectra of 10 μM MagZet1 in the presence of increasing concentrations of $CaCl_2$ in aqueous buffer (50 mM PIPES, 100 mM KCl, pH = 7.0) at 25 °C. (C) Non-linear fit of the fluorescence isotherm, λ_{exc} = 390 nm.

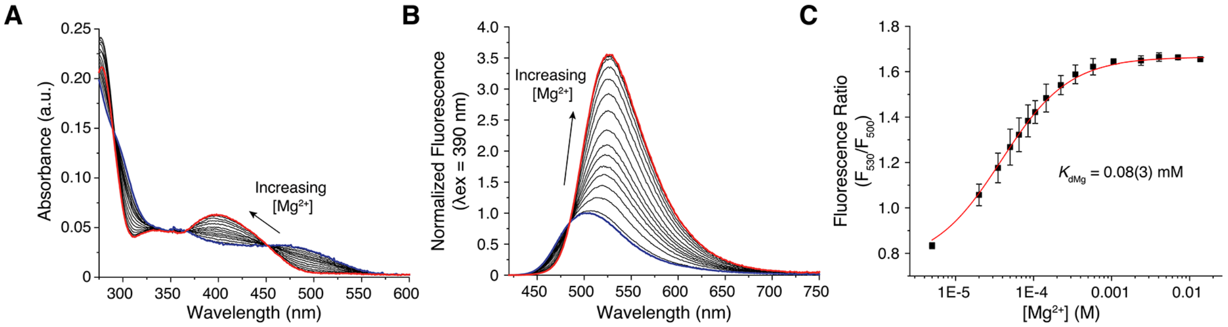

Figure S5. Absorption (A) and fluorescence (B) spectra of 10 μM MagZet1 in the presence of increasing concentrations of $MgCl_2$ in aqueous buffer (50 mM PIPES, 100 mM KCl, pH = 7.0) at 37 °C. (C) Non-linear fit of the fluorescence isotherm, λ_{exc} = 390 nm.

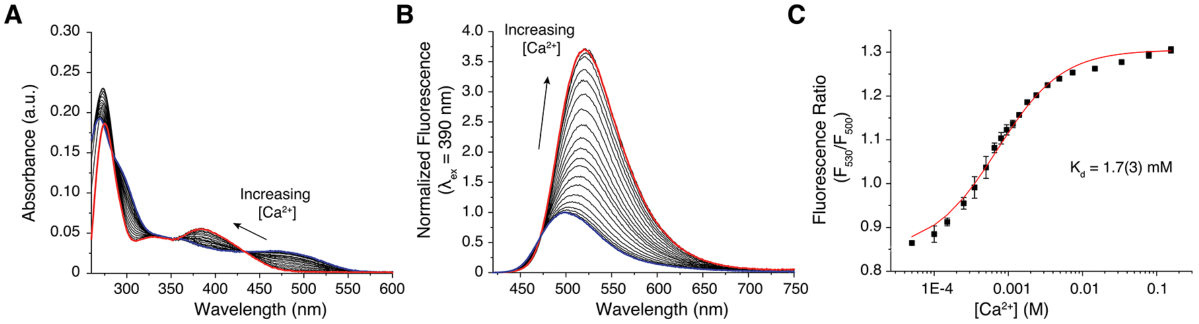

Figure S6. Absorption (A) and fluorescence (B) spectra of 10 μM MagZet1 in the presence of increasing concentrations of $CaCl_2$ in aqueous buffer (50 mM PIPES, 100 mM KCl, pH = 7.0) at 37 °C. (C) Non-linear fit of the fluorescence isotherm, λ_{exc} = 390 nm.

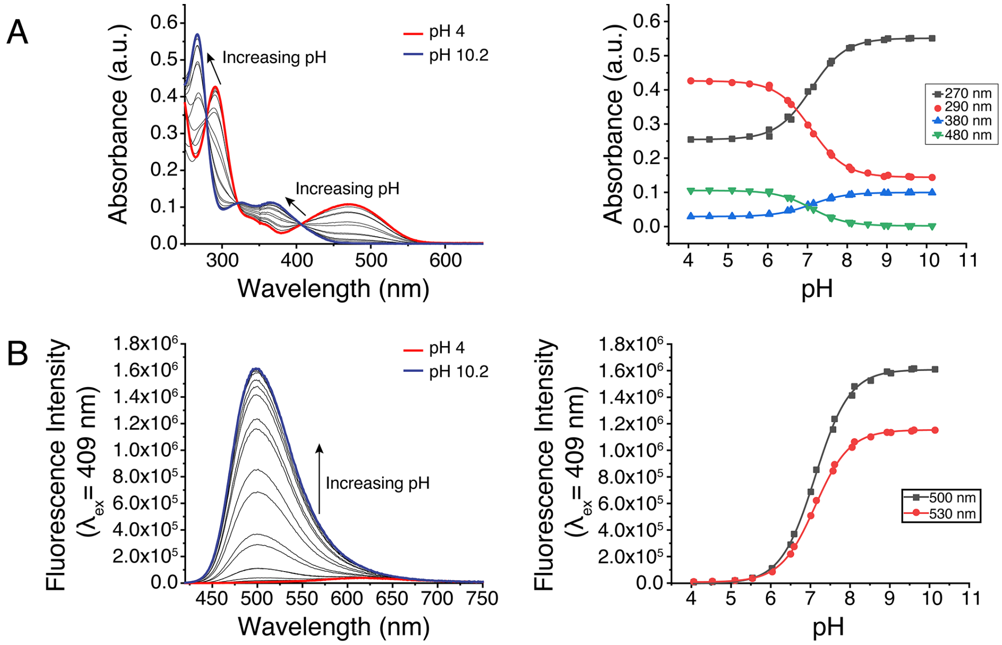

Figure S7. Absorption (A) and fluorescence emission (B) profiles of MagZet1 as a function of pH. Right: Nonlinear fit of the data at selected wavelengths.

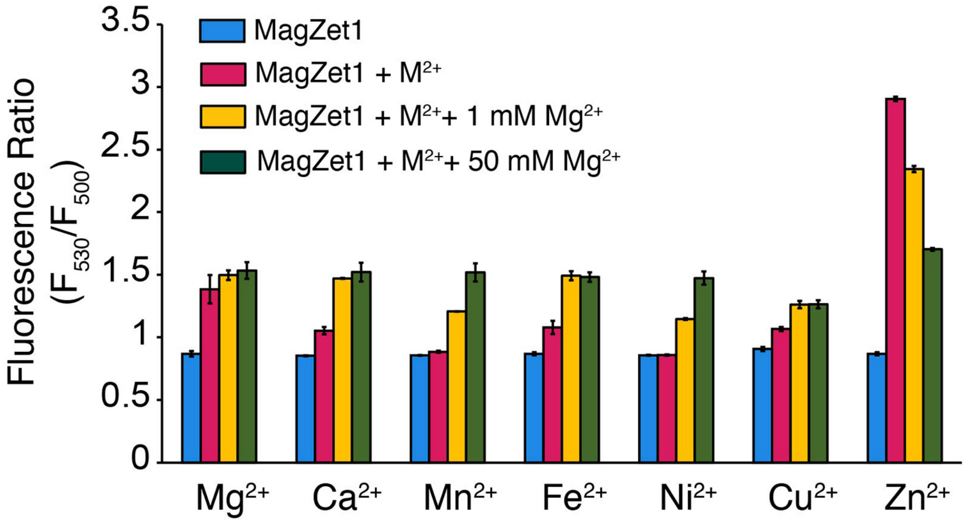

Figure S8. Metal selectivity profile of MagZet1 at pH 6. Fluorescence ratio of 5 μM MagZet1 in aqueous buffer at 25°C (blue bars); in the presence of biologically-relevant divalent cations (1 mM Mg^{2+}, 50 μM Ca^{2+}, or 5 μM for other metals, red bars); in the presence of Mg^{2+} (1 mM) and competing cations (yellow bars); or in the presence of saturating Mg^{2+} (50 mM) and competing cations (green bars). Error bars correspond to the standard deviation of triplicate experiments. (λ_{exc} = 390 nm)

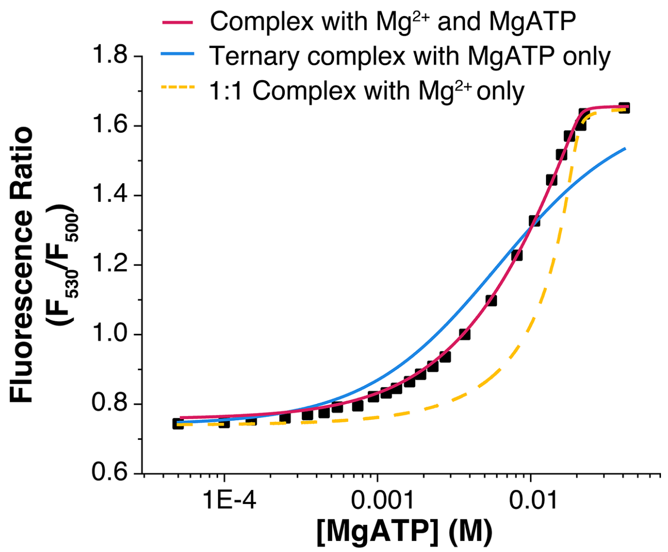

Figure S9. Nonlinear fit of the titration curve of MagZet1 in the presence of increasing amounts of MgATP. Yellow curve: fit considering only formation of a Sensor·Mg^{2+} complex. Blue curve: fit considering only formation of ternary complex Sensor·Mg^{2+}·ATP. Red curve: fit considering both the formation of a Sensor·Mg^{2+} binary complex and Sensor·Mg^{2+}·ATP ternary complex.

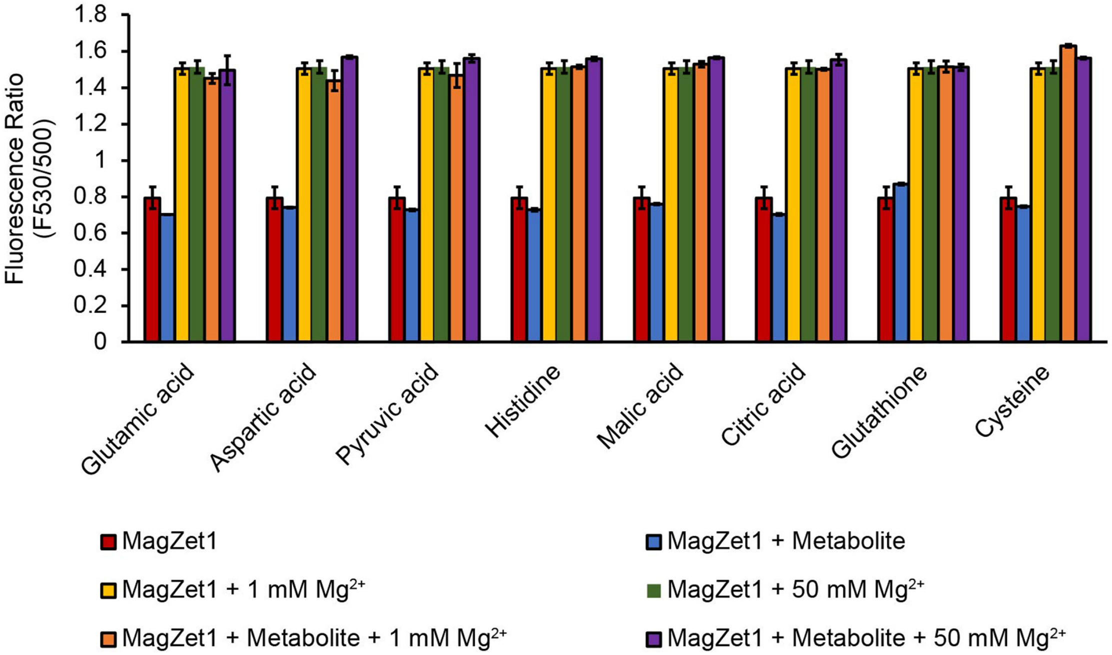

Figure S10. Fluorescence ratio of 5 μM MagZet1 in aqueous buffer at pH 7.0, 25°C (red bars); in the presence of biologically-relevant anions and metabolites (50 mM L-glutamic acid, 15 mM L-aspartic acid, 5 mM pyruvic acid, 1 mM other metabolites; blue bars); in the presence of Mg^{2+} (1 mM) and metabolites (orange bars); or in the presence of saturating Mg^{2+} (50 mM) and metabolites (purple bars). Error bars correspond to the standard deviation of triplicate experiments. (λ_{exc} = 390 nm).

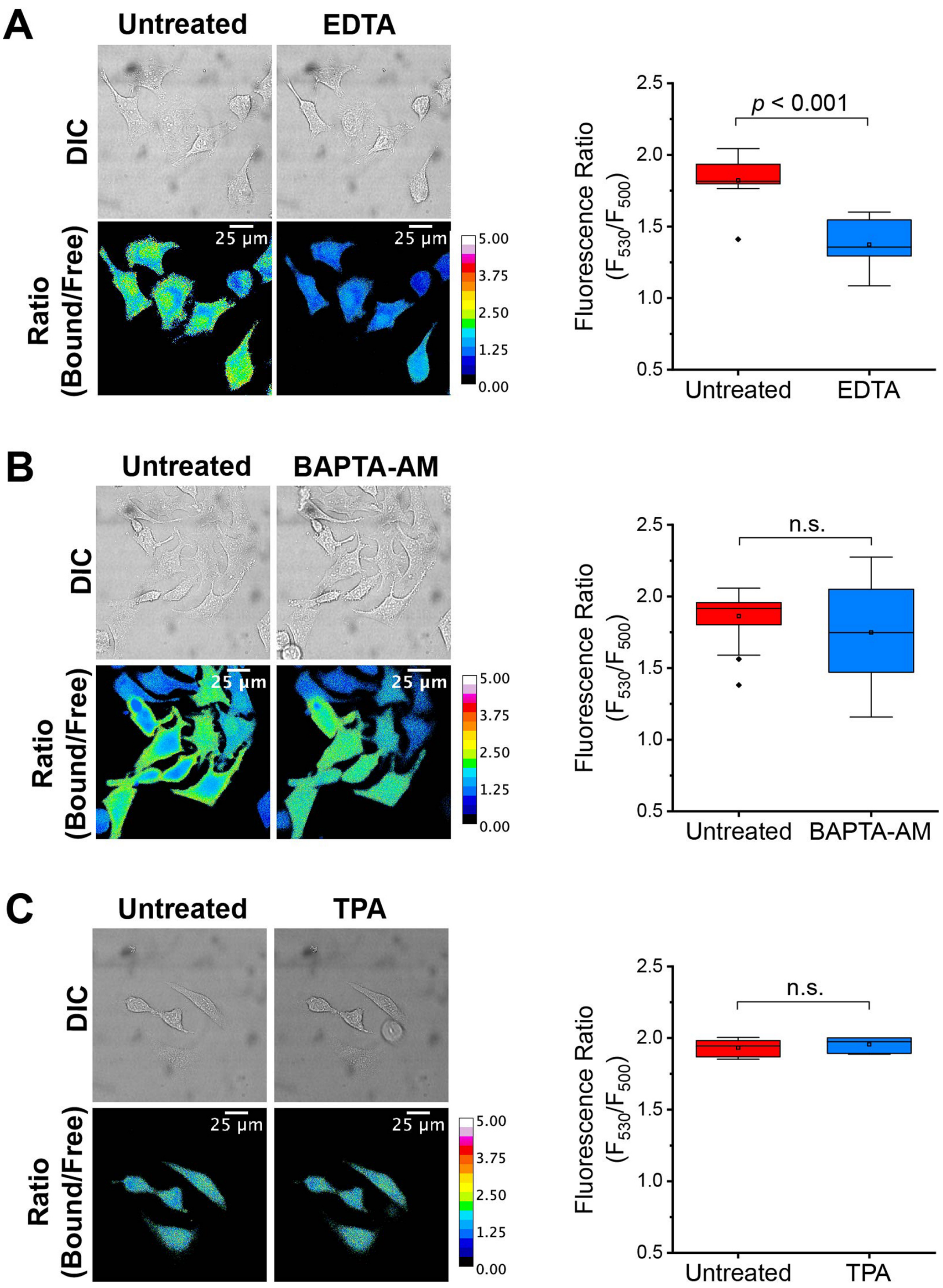

Figure S11. Fluorescence images of HeLa cells stained with 5 µM MagZet1AM before and after treatment with (A) EDTA + ionophore, (B) BAPTA-AM, a Ca^{2+} chelator, or (C) TPA, a zinc chelator.

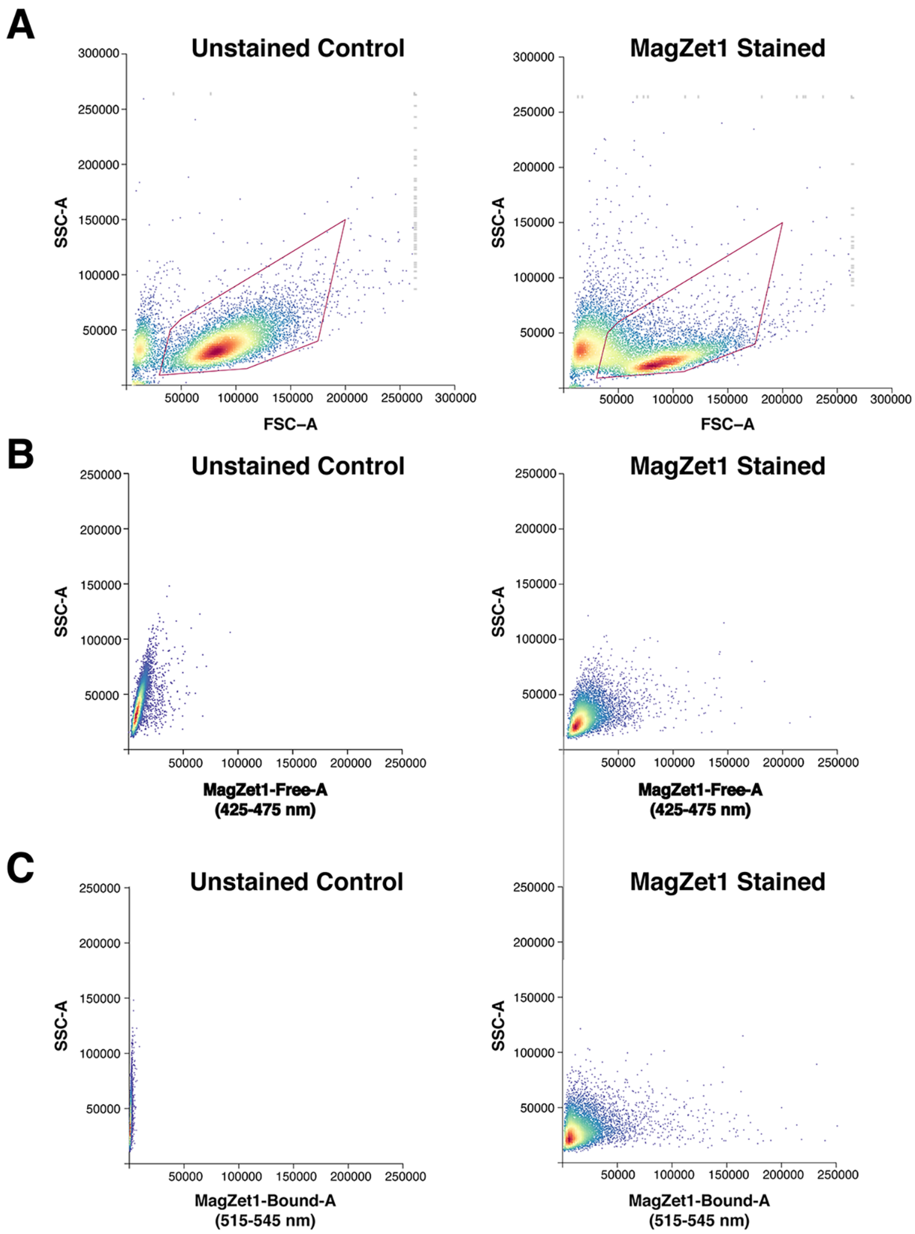

Figure S12. MagZet1 stains HeLa cells and does not lead to a significant change in size or granularity. Scatterplots of the (A) forward v. side scattered light, (B) fluorescence from the "free" sensor v. side scattered light, and (C) fluorescence from the "bound" sensor v. side scattered light for unstained HeLa cells (left) compared to HeLa cells stained with 5μM MagZet1-AM. Red lines represent the cell debris exclusion gate.

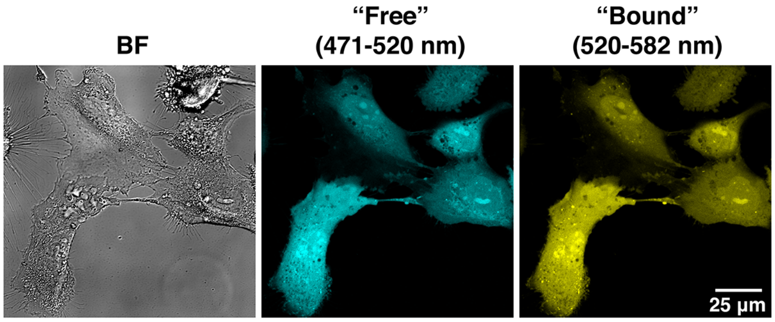

Figure S13. Fluorescence microscopy images of live THLE-2 cells stained with 5 µM MagZet1AM showing diffuse cytosolic signal.

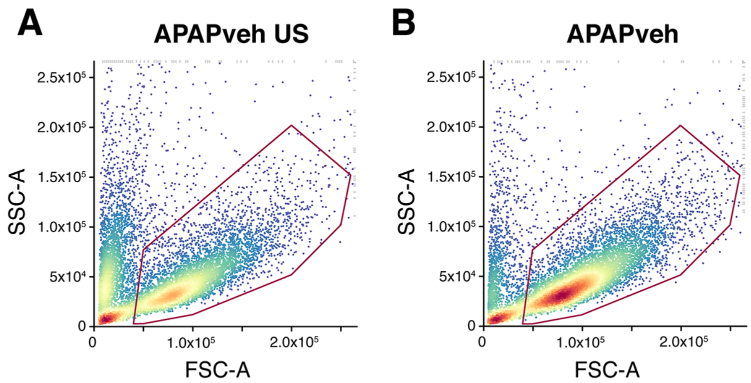

Figure S14. Staining THLE-2 cells with MagZet1 does not lead to a significant change in size or granularity. Scatterplots of the forward v. side scattered light for (A) unstained THLE-2 cells or (B) THLE-2 cells stained with 5µM MagZet1-AM treated with DMSO vehicle.

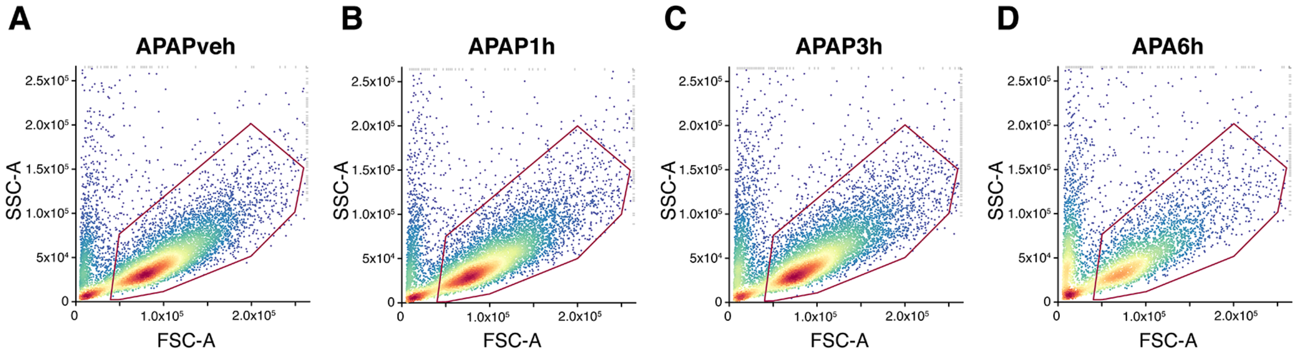

Figure S15. Treatment of THLE-2 cells with 10 mM APAP does not lead to a significant change in size or granularity over the course of the experiment. Scatterplots of the forward v. side scattered light for (THLE-2 cells stained with 5µM MagZet1-AM treated with (A) DMSO vehicle, or 10 mM APAP for (B) 1 h, (C) 3 h, or (D) 6 h.

2 Experimental Details

2.1 Synthesis of new compounds

2.1.1 General synthetic details

All reagents were purchased from commercial sources and used as received. Solvents were purified and degassed by standard procedures. Bromomethyl acetate was prepared as described by Grynkiewicz[1] and Conway[2]. Analytical thin layer chromatography (TLC) was conducted on SorbTech polyester-backed 200 μm silica gel sheets. NMR spectra were acquired on Bruker Avance III 400 and Avance III HD 400 MHz spectrometers. ^{1}H NMR chemical shifts are reported in ppm relative to $SiMe_4$ (δ = 0) and were referenced internally with respect to residual protio impurity in the solvent (δ = 7.26 for $CDCl_3$, 2.05 for Acetone-d_6, 3.31 for Methanol-d_4). ^{13}C NMR chemical shifts are reported in ppm relative to $SiMe_4$ (δ = 0) and were referenced internally with respect to the solvent signal (δ = 77.16 for $CDCl_3$, 29.84 for Acetone-d_6, 49.00 for Methanol-d_4). Coupling constants are reported in Hz. High resolution mass spectra (HRMS) were acquired on an Agilent 6224 Accurate-Mass TOF LC/MS using APCI or ESI ionization. Reversed phase HPLC analyses were conducted using an Agilent 1260 Infinity system with UV-vis and fluorescence detection using a Zorbax Extend C18 reversed phase column (4.6×50 mm, 1.8 μm particle size) and a gradient from 10% to 100% acetonitrile/water (+ 0.1% trifluoroacetic acid). Low resolution mass spectra (LRMS) were acquired on an Agilent 6120 Single Quadrupole LCMS spectrometer using ESI ionization and a Kinetex C18 reversed phase column (3×50 mm, 2.6 μm particle size) and a gradient of 5% to 100% acetonitrile/water (+ 0.1% formic acid).

2.1.2 Synthesis of MagDMA

Synthesis of dimethyl 2-((2-(methoxycarbonyl)phenyl)amino)maleate (1)

A solution of methyl anthranilate (1.4 mL, 10.8 mmol) and dimethyl acetylene dicarboxylate (1.6 mL, 13.1 mmol, 1.2 equiv.) in methanol (15 mL) was stirred at room temperature overnight. After 15 h, solvent was removed *in vacuo* and the crude mixture was purified by flash chromatography

(SiO_2, 0-20% ethyl acetate/hexanes, R_f = 0.38 in 20% ethyl acetate/hexanes) to yield the desired product as a pale yellow solid (3.089 g, 98%). Characterization matched reported values.[3]

Synthesis of dimethyl 4-oxo-1,4-dihydroquinoline-2,8-dicarboxylate (2)

In a high pressure vial, dimethyl 2-((2-(methoxycarbonyl)phenyl)amino)maleate (1.2 g, 4.0 mmol) was suspended in diphenyl ether (10 mL) and heated to 250 °C in a heating block. After 1 h, the reaction was removed from heat and cooled to room temperature. The brown solution was poured into hexanes (700 mL). The resulting precipitate was collected by vacuum filtration, washed with excess hexanes, and dried to yield the desired product as a pale brown solid (635.5 mg, 61%). Product characterization matched reported values.[3]

Synthesis of dimethyl 6-nitro-4-oxo-1,4-dihydroquinoline-2,8-dicarboxylate (3)

A suspension of dimethyl 4-oxo-1,4-dihydroquinoline-2,8-dicarboxylate (502.7 mg, 1.93 mmol) in concentrated H_2SO_4 (4 mL) was stirred until homogeneous and cooled to 0 °C. Nitric acid (340 μL) was then added dropwise. The reaction was allowed to warm to room temperature over 4 h and stirred overnight. After 23 h, the reaction was poured into ice water (100 mL) and vigorously stirred, during which a bright yellow solid formed. The precipitate was collected and dried under vacuum to yield the desired product as a bright yellow powder (508.1 mg, 86%).

^{1}H NMR (400 MHz, $CDCl_3$, δ) 12.49 (s, 1H), 9.40 (dd, *J* = 2.7, 0.7 Hz, 1H), 9.22 (d, *J* = 2.7 Hz, 1H), 7.07 (d, *J* = 1.7 Hz, 1H), 4.10 (d, *J* = 8.1 Hz, 6H). ^{13}C{^{1}H} NMR (101 MHz, $CDCl_3$, δ) 178.20, 166.44, 162.30, 143.28, 138.04, 130.30, 128.48, 126.95, 117.17, 113.90, 77.48, 77.16, 76.84, 54.32, 53.67. ESI-MS (*m/z*): $[M+H]^+$ calcd for $C_{13}H_{10}N_2O_7$, 307.1; found 307.0

Synthesis of dimethyl 6-amino-4-oxo-1,4-dihydroquinoline-2,8-dicarboxylate (4)

A solution of dimethyl 6-nitro-4-oxo-1,4-dihydroquinoline-2,8-dicarboxylate (107.35 mg, 0.33 mmol) and 10% Pd/C (45.6 mg, 13% with respect to the nitro compound) in methanol was stirred under a balloon of H_2 for 1 h. The crude mixture was filtered through celite. The filtrate was

collected and evaporated to produce the title compound as a brown solid (87.9 mg, 91%). The compound was used without further purification.

^1H NMR (400 MHz, $CDCl_3$, δ) 12.17 (s, 1H), 7.90 – 7.81 (m, 2H), 6.93 (s, 1H), 4.03 (d, *J* = 7.9 Hz, 6H), 3.97 (brs, 2H). $^{13}C\{^1H\}$ NMR (101 MHz, $CDCl_3$, δ) 178.46, 167.57, 163.15, 142.68, 136.22, 133.39, 128.95, 124.86, 117.23, 114.95, 110.46, 77.48, 77.16, 76.84, 53.79, 52.94. ESI-MS (*m/z*): $[M+H]^+$ calcd for $C_{13}H_{12}N_2O_5$, 277.1; found 277.1

Synthesis of dimethyl 6-(dimethylamino)-4-oxo-1,4-dihydroquinoline-2,8-dicarboxylate (5)

Dimethyl 6-amino-4-oxo-1,4-dihydroquinoline-2,8-dicarboxylate (50.8 mg, 0.2 mmol) and paraformaldehyde (166.5 mg, 6 mmol, 30 equiv) were combined in glacial acetic acid (3 mL). The reaction mixture was stirred at room temperature for 30 min and treated with $NaB(CN)H_3$ (22.2 mg, 0.4 mmol, 2 equiv), then stirred overnight. After 22 h, the reaction mixture was neutralized by the addition of sat. $NaHCO_3$(aq) and extracted into dichloromethane (3×). The combined organics were washed with brine (2×), dried over Na_2SO_4, and evaporated to yield a bright orange solid as the desired product (30.0 mg, quant.).

^1H NMR (400 MHz, $CDCl_3$, δ): 12.20 (s, 1H), 7.95 (d, *J* =3.2 Hz, 1H), 7.76 (d, *J* =3.1 Hz, 1H), 6.97(d, *J* =1.6 Hz, 1H), 4.04 (s, 3H), 4.03 (s, 1H), 3.09 (s, 6H). $^{13}C\{^1H\}$ NMR (100 MHz, $CDCl_3$, d): 178.5,167.9, 163.2, 146.6, 135.7, 131.9, 128.8, 122.5, 116.9, 111.8, 110.0, 53.7, 52.9, 40.8. ESI-MS (*m/z*): [M+H]+ calculated for $C_{15}H_{16}N_2O_5$, 305.1; found 305.0

Synthesis of MagDMA Methyl Ester (6)

A solution of dimethyl 6-(dimethylamino)-4-oxo-1,4-dihydroquinoline-2,8-dicarboxylate (27.1 mg, 0.09 mmol) in dichloromethane (700 μL) in a sealed vial was treated with $POCl_3$ (800 μL, 0.09 mmol, 1 equiv.) dropwise. The reaction was warmed to 42 °C and stirred for 30 min. The reaction was then cooled to 0 °C and treated with sat. $NaHCO_3$(aq) until a yellow color persisted. The neutralized mixture was then stirred at room temperature for 15 min and extracted into dichloromethane (3×). The combined organics were washed with water (1×) and dried over

Na_2SO_4. Evaporation of solvent produced the final product as a bright orange solid (32.6 mg, quant.).

^{1}H NMR (400 MHz, CDCl3, δ) 8.20 (s, 1H), 7.80 (d, *J* = 3.0 Hz, 1H), 7.19 (d, *J* = 3.0 Hz, 1H), 4.04 (d, *J* = 23.9 Hz, 6H), 3.19 (s, 6H). ^{13}C{^{1}H} NMR (100 MHz, $CDCl_3$, d): 1.67.7, 165.5, 149.2, 143.3, 140.6, 139.1, 133.5, 129.8, 122.2, 121.8, 103.1, 53.1, 53.0, 40.5. ESI-MS (*m/z*): $[M+H]^+$ calculated for $C_{15}H_{16}N_2O_5$, 323.1; found 323.0. HR-TOF-MS (*m/z*): $[M+H]^+$ calculated for $C_{15}H_{16}N_2O_5$, 323.0793; found 323.0796

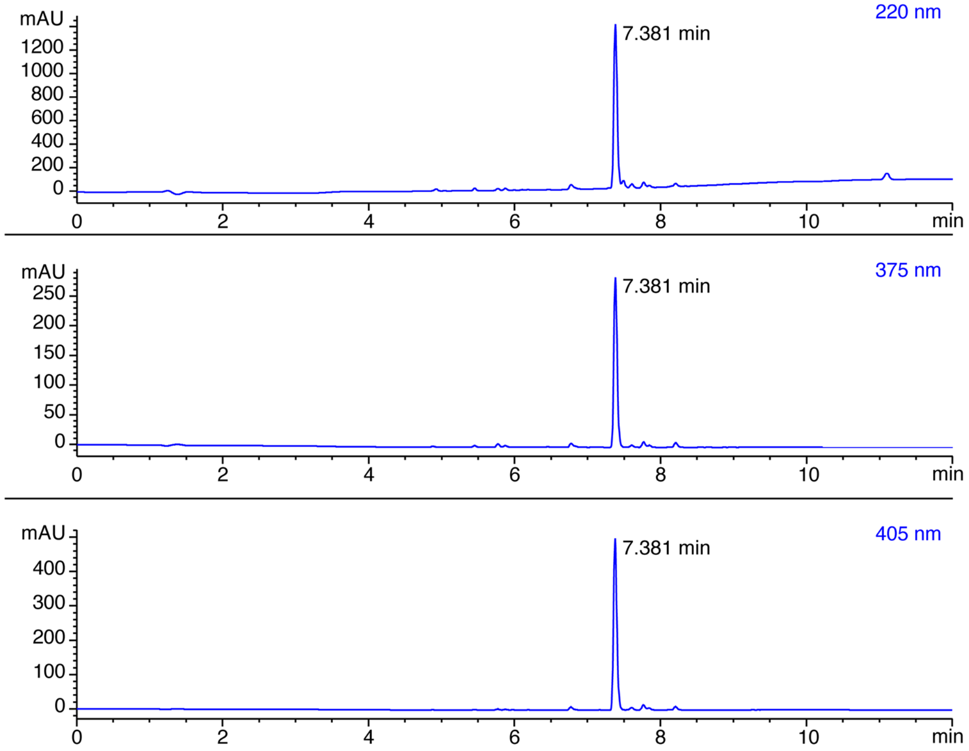

Figure S16. HPLC chromatogram of MagDMA methyl ester.

Quantitative hydrolysis of MagDMA methyl ester (6) to MagDMA (7)

In a typical experiment, a quantitative sample of dimethyl 4-chloro-6-(dimethylamino)quinoline-2,8-dicarboxylate (9.25 mg, 0.029 mmol) in THF (150 µL) was treated with 150 µL of 1 M KOH. After 2 h, the reaction mixture was transferred quantitatively to a volumetric flask and diluted to

2 mL with PIPES buffer (pH 7.04) to yield a 14.33 mM stock solution of MagDMA for spectroscopic studies. Reaction completion was verified by HPLC.

^{1}H NMR (400 MHz, NaOD, δ): 8.07 (s, 1H), 7.52 (d, J = 2.8 Hz, 1H), 7.14 (d, J = 2.8 Hz, 1H), 3.08 (s, 6H). ESI-MS (m/z): $[M+H]^+$ calculated for $C_{13}H_{11}ClN_2O_4$, 295.0; found 294.9

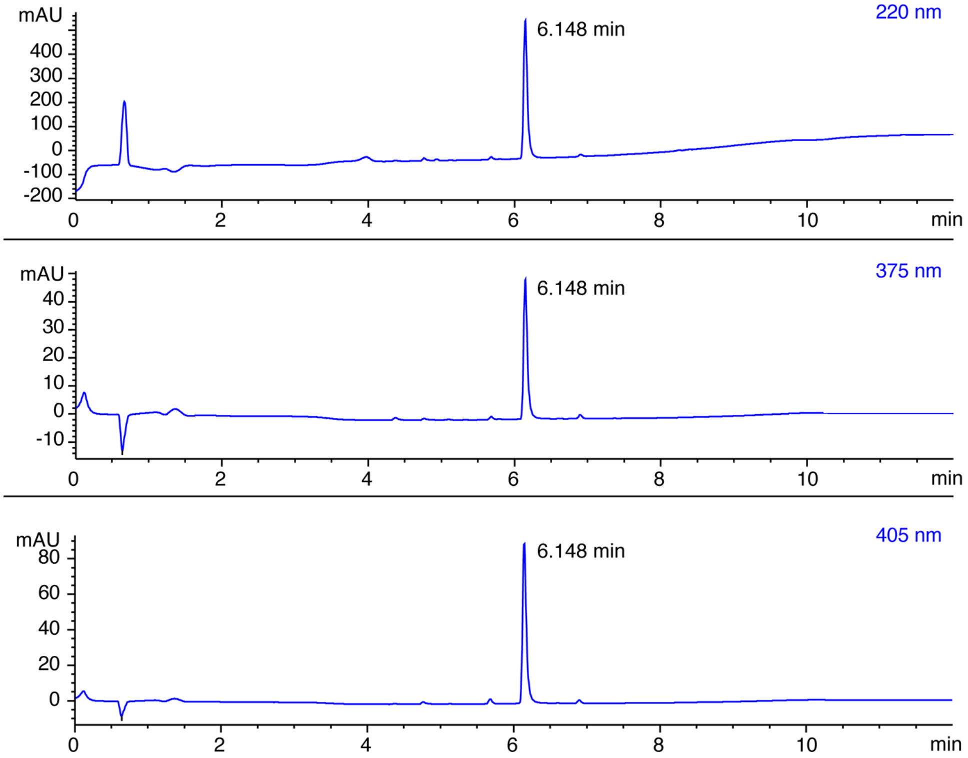

Figure S17. HPLC chromatogram of the fully hydrolyzed MagDMA.

2.1.3 Synthesis of MagZet1

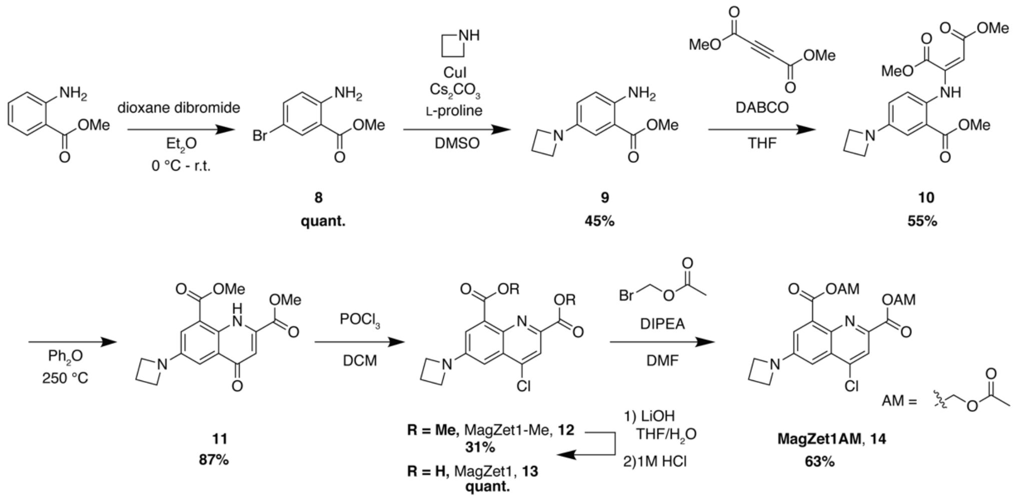

Synthesis of methyl 2-amino-5-bromobenzoate (8)

The brominated anthranilate was prepared following a modified procedure from Chaudhuri et. al.[4] Briefly, to a solution of methyl anthranilate (1 mL, 7 mmol, 1 equiv.) in diethyl ether (3 M) was added dioxane dibromide (3 g, 1.15 equiv.) at 0 °C. The reaction was then stirred and allowed to gradually warm to room temperature over 50 min, over which a precipitate formed. The waxy brown solid was collected by vacuum filtration, washed with cold ether (3×) and DCM (1×), and dried under vacuum to give the desired product in a quantitative yield. Characterization matched reported values.[4]

Synthesis of methyl 2-amino-5-(azetidin-1-yl)benzoate (9)

Under an N_2 atmosphere, methyl 2-amino-5-bromobenzoate (512, mg, 2.2. mmol), L-proline (75 mol%), and oven-dried Cs_2CO_3 (860 mg, 1.2 equiv.) were combined in anhydrous DMSO (4 mL). Azetidine (200 µL, 1.4 equiv.) was added followed by copper iodide (50 mol% with respect to the bromobenzoate). The reaction was heated to 90 °C and stirred overnight. After 18 h, the reaction was diluted with ethyl acetate and washed with water (2×) and brine. The aqueous layers were combined and extracted with ethyl acetate (2×). The organic layers were combined and dried over

Na_2SO_4. Evaporation under reduced pressure produced a brown solid that was purified by flash chromatography (SiO_2, 20-30% ethyl acetate/hexanes, R_f = 0.17 in 20% ethyl acetate/hexanes) to yield the desired product as a bright yellow solid (204.7 mg, 45%).

^{1}H NMR (400 MHz, $CDCl_3$, δ): 6.95 (d, *J*= 2.0 Hz, 1H), 6.63-6.60 (m, 2H), 5.27 (br s, 2H), 3.87 (s, 3H), 3.78 (t, *J* = 7.1 Hz, 4H), 2.32 (quint., *J* = 7.1 Hz, 2H). $^{13}C\{^1H\}$ NMR (100 MHz, $CDCl_3$, δ): 168.5, 144.0, 142.9, 119.9, 117.9, 112.5, 111.3, 53.1, 51.4, 17.1. ESI-MS (*m/z*): $[M+H]^+$ calcd for $C_{11}H_{14}N_2O_2$,, 207.1 found 207.0. Mp 139-142 °C.

Synthesis of dimethyl 2-((4-(azetidin-1-yl)-2-(methoxycarbonyl)phenyl)amino)maleate (10)

To a solution of methyl 2-amino-5-(azetidin-1-yl)benzoate (209.1 mg, 1.01 mmol) and 1,4-diazabicyclo[2.2.2]octane (10 mol%) in THF (10 mL) in a water bath at room temperature was added dimethylacetylene dicarboxylate (150 µL, 1.2 equiv.) dropwise over 5 min. The reaction was stirred at room temperature for 48 h after which no starting material was observed by NMR. The reaction mixture was concentrated and purified by column (SiO_2, 20% ethyl acetate/hexanes, R_f = 0.23) to give the desired product as an orange solid (195.0 mg, 55%).

^{1}H NMR (400 MHz, $CDCl_3$, δ): 11.07 (s, 1H), 6.99 (d, *J* = 2.8 Hz, 1H), 6.60 (d, *J* = 8.7 Hz, 1H), 6.50 (dd, *J* = 8.70, 2.8 Hz, 1H), 5.37 (s, 1H), 3.34 (s, 3H), 3.86 (t, *J* = 7.2 Hz, 4H), 3.76 (s, 3H), 3.69 (s, 3H), 2.36 (quint. *J* = 7.21 Hz, 2H). $^{13}C\{^1H\}$ NMR (100 MHz, $CDCl_3$, δ): 169.2, 167.4, 165.8, 147.9, 146.2, 132.9, 120.8, 119.4, 116.5, 113.3, 94.8, 52.9, 52.8, 52.4, 51.4, 17.1. ESI-MS (*m/z*): $[M+H]^+$ calcd for $C_5H_{10}BrNO$, 349.1 found 349.0. Mp 129-133 °C.

Synthesis of dimethyl 6-(azetidin-1-yl)-4-oxo-1,4-dihydroquinoline-2,8-dicarboxylate (11)

In a 5 mL vessel, a suspension of dimethyl-2-((4-(azetidin-1-yl)20(methoxycarbonyl) phenyl)maleate (309 mg, 0.89 mmol) in diphenyl ether (4 mL) was gently heated to dissolve all solids. The reaction mixture was then stirred, heated to 250°C, and exposed to microwave irradiation for 45 min. The reaction was cooled to room temperature and poured into 700 mL hexanes. The resulting precipitate was collected by vacuum filtration and washed with excess

hexanes. Drying under vacuum produced the title compound as a dark orange solid (243.7 mg, 87%).

^{1}H NMR (400 MHz, $CDCl_3$, δ): 12.22 (br s, 1H), 7.58 (d, *J* = 2.8 Hz, 1H), 7.52 (d, *J* = 2.7 Hz, 1H), 6.94 (d, *J* = 1.8 Hz, 1H), 4.05-3.99 (m, 10H), 2.44 (quint. *J* = 7.3 Hz, 2H). $^{13}C\{^{1}H\}$ NMR (100 MHz, $CDCl_3$, δ): 178.5, 167.8, 163.2, 148.1, 135.9, 132.3, 128.8, 121.1, 116.9, 111.0, 110.2, 53.7, 52.9, 52.8, 17.0. ESI-MS (*m/z*): $[M+H]^+$ calcd for $C_{16}H_{16}N_2O_5$, 317.1 found 317.0

Synthesis of MagZet1 Methyl Ester (12)

To a solution of dimethyl 6-(azetidin-1-yl)-4-oxo-1,4-dihydroquinoline-2,8-dicarboxylate (97.3 mg, 0.3 mmol) in THF (3 mL) was added $POCl_3$ (60 µL, 1 equiv.) dropwise. The reaction was stirred for 30 min after which starting material was not observed by TLC. The reaction was diluted in 200 mL ice water and stirred for 10 min. The aqueous mixture was extracted with ethyl acetate (3×). The aqueous layer was neutralized by the addition of saturated $NaHCO_3$(aq) and further extracted with ethyl acetate (1×). The organic layers were combined and dried over Na_2SO_4. Evaporation under reduced pressure produced a brown solid that was purified by column (SiO_2, 30% ethyl acetate/hexanes R_f = 0.17) to yield the title product as a yellow solid (32.3 mg, 31%).

^{1}H NMR (400 MHz, $CDCl_3$, δ): 8.19 (s, 1H), 7.39 (d, *J* = 2.6 Hz, 1H), 6.90 (*J* = 3.2 Hz, 1H), 4.13 (t, *J* = 7.3 Hz, 4H), 4.06 (s, 3H), 4.00 (s, 3H), 2.50 (quint. *J* = 7.4 Hz, 2H). $^{13}C\{^{1}H\}$ NMR (100 MHz, $CDCl_3$, δ): 167.5, 165.5, 150.1, 143.3, 140.3, 139.6, 133.8, 129.8, 122.2, 120.3, 101.6, 53.1, 52.9, 52.0, 16.6. ESI-MS (*m/z*): $[M+H]^+$ calcd for $C_{16}H_{15}ClN_2O_4$, 335.1 found 335.0. HR-TOF-MS (*m/z*): $[M+H]^+$ calcd for $C_{16}H_{15}ClN_2O_4$, 335.0793; found 335.0777. Mp 169-173 °C.

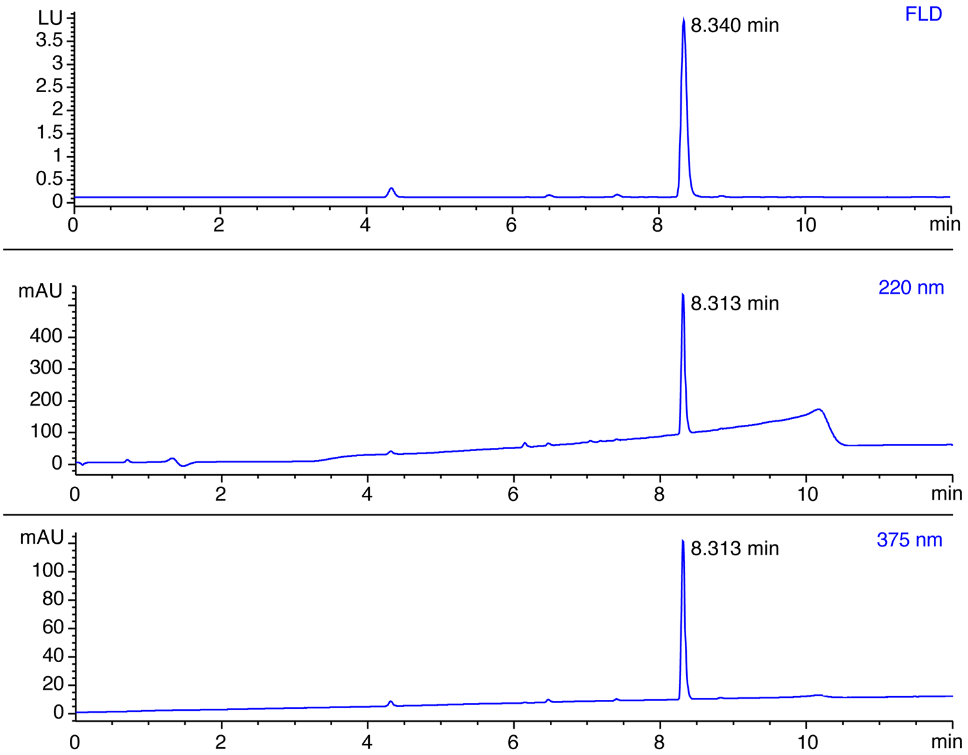

Figure S18. HPLC chromatogram of MagZet1 methyl ester, compound **12**.

Hydrolysis of MagZet1 methyl ester (12) to MagZet1 (13)

In a typical experiment, a quantitative sample of MagZet1 methyl ester, compound **12** (4.43 mg, 0.013 mmol), in THF (200 μL) was treated with 1 M LiOH (200 μL). The reaction was then stirred at room temperature. After 5 h, the reaction was transferred quantitatively to a volumetric flask and diluted in PIPES buffer (pH 7). Quantitative conversion was verified by HPLC. Aliquots of the solution were flash-frozen and stored at -20 °C until use for spectroscopic studies.

^{1}H NMR (400 MHz, NaOD, δ): 8.00 (s, 1H), 7.29 (d, J = 2.6 Hz, 1H), 6.96 (d, J = 2.6 Hz, 1H), 3.99 (t, J = 7.4 Hz, 4H), 2.38 (q , J = 7.4 Hz, 2H). ESI-MS (*m/z*): $[M+H]^+$ calcd for $C_{14}H_{11}ClN_2O_4$ 307.0, found 307.0.

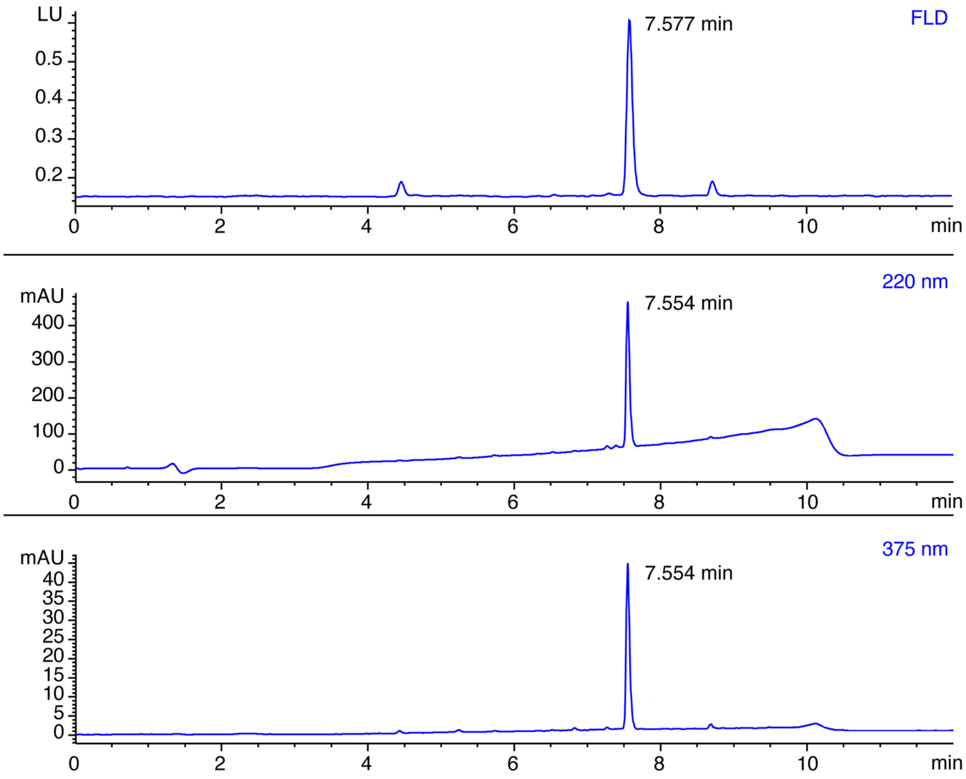

Figure S19. HPLC chromatogram of MagZet1, compound **13**.

Synthesis of MagZet1AM (14)

A solution MagZet1 methyl ester, compound **12** (8.8 mg, 0. 025 mmol), in THF (400 µL) was treated with 1 M LiOH (400 µL). The reaction was then stirred at room temperature and monitored by HPLC for the complete consumption of starting material. The reaction mixture was then neutralized by the dropwise addition of 1 M NaOH and lyophilized. The resulting powder was redissolved in anhydrous DMF (2 mL) and treated with anhydrous Hünig's base (70 µL, 16 equiv.) followed by bromomethylacetate (70 µL, 29 equiv.). The reaction was stirred for 2 h and monitored by HPLC for the disappearance of starting material. The solvent was evaporated and the resulting residue was taken up in ethyl acetate (50 mL) and washed with brine (5x). The combined organics were dried over Na_2SO_4 and evaporated to yield an orange residue. Purification by column chromatography (SiO_2, 0.8:7.2:2 Ethyl Acetate/Dichloromethane/Hexanes, R_f = 0.4) yielded the desired product as a yellow solid (7.3 mg, 63%).

1H NMR (400 MHz, $CDCl_3$, δ): 8.16 (s, 1H), 7.40 (d, J = 2.6, 1H), 6.90 (d, J = 2.6, 1H), 6.09 (s, 2H, 6.06 (s, 2H), 4.17 (t, J = 7.3, 4H), 2.56 (quin. J = 7.4, 2H), 2.20 (s, 3H), 2.14 (s, 3H). 13C{1H} NMR (100 MHz, $CDCl_3$, δ): 170.0, 169.7, 165.8, 163.4, 150.1, 142.1, 140.2, 139.6, 132.9, 122.5, 120.6, 101.7, 80.3, 52.0, 21.0, 20.9, 16.6. ESI-MS (m/z): calcd for $C_{20}H_{19}ClN_2O_8$, 451.1 found 451.0. HR-TOF-MS (*m/z*): $[M+H]^+$ calcd for $C_{20}H_{19}ClN_2O_8$ 450.0830; found 450.0823

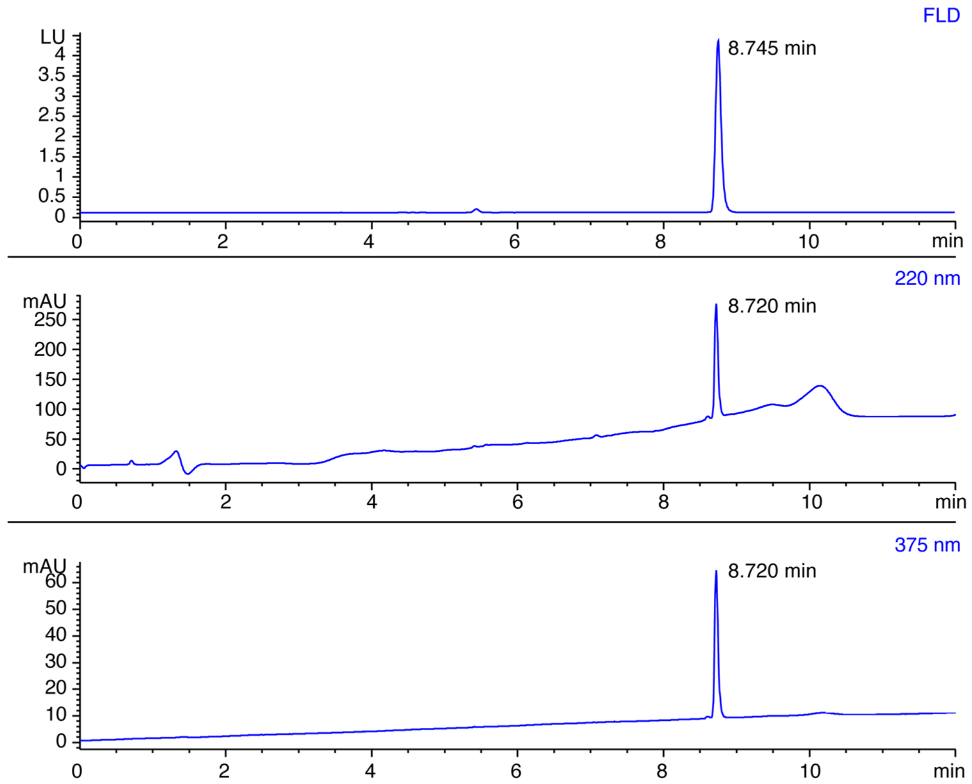

Figure S20. HPLC chromatograms of MagZet1-AM, compound **14**.

2.2 Spectroscopic Studies

2.2.1 General spectroscopic details

All aqueous solutions were prepared using deionized water having a resistivity of ≥ 18.2 MΩ·cm. Piperazine-N,N-bis(2-ethanesulfonic acid) (PIPES), 4-(2-hydroxyethyl)-1-piperazineethane sulfonic acid (HEPES), 2-ethanesulfonic acid (MES), 99.999% KCl, 99.999% $MgCl_2$, 99.999%

$ZnCl_2$, 99.99% $CaCl_2$, and 99.99% semiconductor grade KOH pellets were purchased from Sigma Aldrich. Trace metal grade 37% solution of HCl was purchased from Fisher Scientific. Spectroscopic measurements were conducted at pH 7.0 in aqueous buffer containing 50 mM PIPES, 100 mM KCl. Aqueous PIPES buffer was treated with CHELEX resin (Bio-Rad) according to the manufacturer protocol to remove trace metal ions. Absorption spectra were collected on a Cary 100 UV-Vis spectrophotometer using 1 cm quartz cuvettes. The temperature during analysis was maintained within ±0.02 °C by using a Quantum Northwest TC225 Temperature Controller. Fluorescence spectra were acquired on a QuantaMaster 40 Photon Technology International spectrofluorometer equipped with xenon lamp source, emission and excitation monochromators, excitation correction unit, and PMT detector. Emission spectra were corrected for the detector wavelength-dependent response. Measurements were conducted at 25.0 ± 0.1 °C and 37.0 ± 0.1 °C maintained by a Quantum Northwest Temperature Controller. Fluorescence quantum yields were determined using 5–50 µM solutions of the sensor in aqueous buffer at pH 7.0, exciting at 390 nm. Solutions of quinine sulfate in 0.5 M H_2SO_4 with a reported quantum yield of 0.54 at 347 nm excitation[5] were used as standards. Fluorescence emission was integrated over 490 to 750 nm. Data for metal selectivity studies were collected on a FlexStation 3 Multi-Mode Microplate Reader from Molecular Devices. The sensor was excited at 390 nm and the fluorescence intensity recorded at 500 and 530 nm to calculate a ratio, which replicates conditions used in imaging and cytometry experiments.

2.2.2 Spectroscopic Measurements

Determination of metal dissociation constants

Fluorescence titrations with $MgCl_2$ and $CaCl_2$ were conducted using 10 µM solutions of MagZet1 or 25 µM solutions of MagDMA in aqueous buffer at pH 7.0. Magnesium and calcium were added from a 100 mM or 1 M stock solution to cover a range from 0-50 mM total Mg^{2+} or 0-10 mM total Ca^{2+} in the cuvette. For each titration, metal stock solutions were treated with an appropriate

amount of sensor to match the concentration in the cuvette and prevent sensor dilution over the course of the titration. Values for the dissociation constant were obtained from nonlinear fit using a 1:1 sensor to metal binding model (eq S1) where R represents the ratio of fluorescence intensity at the metal bound (λ_1 = 530 nm for MagZet1 and MagDMA) and metal free (λ_2 = 500 nm for MagZet1, λ_2 = 475 nm for MagDMA) wavelengths ($R=I_{\lambda1}/I_{\lambda2}$) when excited at 390 nm for MagZet1, and at 456 nm for MagDMA; R_{min} is the fluorescence ratio of the metal-free sensor at the start of the titration; R_{max} is the fluorescence ratio of the metal-saturated sensor at the end of the titration; and S_{f2} and S_{b2} are proportionality coefficients for the fluorescence at λ_2 of the metal-free and metal-bound forms of the sensor, respectively.[6]

$$R = \frac{R_{max}[M^{2+}]_f - R_{min}\left(\frac{K_d S_{f2}}{S_{b2}}\right)}{[M^{2+}]_f + \left(\frac{K_d S_{f2}}{S_{b2}}\right)} \qquad \text{(eq S1)}$$

For Ca^{2+} and Mg^{2+} titrations, the approximation $[M^{2+}]_f \approx [M^{2+}]_T$ was made, where $[M^{2+}]_T$ is the total metal concentration. Data were fitted using OriginPro 2021 software. Reported K_d values represent the average of three independent titrations and the error represents the standard deviation.

To determine the affinity of the sensor for Zn^{2+}, titrations were conducted using an EGTA buffer system consisting of 10 mM EGTA and the total $ZnCl_2$ concentration varying from 0 to 1 mM. The concentration of free Zn^{2+} was calculated from the total metal concentration by solving eq S2 with an apparent binding constant for Zn^{2+}-EGTA of $\log K'_{ZnEGTA}$ = 8.24 at pH 7.0.

$$K'_{ZnEGTA}[Zn^{2+}]^2 + \left(1+[EGTA]_t K'_{ZnEGTA}[Zn^{2+}]_t\right)[Zn^{2+}] - [Zn^{2+}]_t = 0 \qquad \text{(eq S2)}$$

The apparent binding constant was calculated from the absolute stability constant $\log K_{ZnEGTA}$ = 12.6 (25 °C, μ = 0.1) listed in Martell and Smith,[7] using Schwarzenbach's α method, according to the following equation:

$$K'_{ZnEGTA} = \alpha_{EGTA^{4-}} K_{ZnEGTA} \quad \text{(eq S3)}$$

Where α_{EGTA4-} corresponds to the fraction of fully deprotonated EGTA at the pH of interest. The pK_a values for EGTA used to calculate α_{EGTA4-} were corrected upwards by 0.11 units to account for the fact that the tabulated pK_a values in Martell and Smith[7] are determined using concentration and not activity of the hydrogen ion. According to the National Bureau of Standards, pH is defined as -log(a_H), where a_H is 0.78[H^+] at 0.1 M ionic strength. As a result, the pK_a values for EGTA employed in our calculations were: pK_{a1} = 9.50, pK_{a2} = 8.88, pK_{a3} = 2.80, pK_{a4} = 2.10.

Selectivity studies

Metal selectivity experiments were conducted using 1 or 5 μM solutions of MagZet1 in aqueous buffer at the specified pH, treated with $CaCl_2$, $MnCl_2$, $FeSO_4$, $CoCl_2$, $NiCl_2$, $CuCl_2$, or $ZnCl_2$ for a final concentration of 1 μM M^{2+} for the *d*-block metals, or 50 μM for Ca^{2+}. Competition experiments were conducted using 1 μM MagZet1 and the concentration of competing metal as described above, in the presence of either 1 mM or 50 mM $MgCl_2$. Fluorescence emission from metal-free sensors was determined using solutions of MagZet1 treated with 10 μM EDTA. All reported values represent the average of three independent measurements with the error representing the standard deviation.

Metabolite interference experiments were conducted using 5 μM solutions of MagZet1 in aqueous buffer at pH 7.0 in the presence of amino acids or metabolites (50 mM (L)-glutamic acid, 15 mM (L)-aspartic acid, 5 mM pyruvic acid, or 1 mM of either (L)-histidine, citric acid, malic acid, glutathione (reduced), or (L)-cysteine). Competition experiments were conducted using 5 μM MagZet1 and the concentration of metabolite as described above, in the presence of either 1 mM

or 50 mM $MgCl_2$. All reported values represent the average of three independent measurements with the error bars representing the standard deviation.

Investigation of ternary complex formation with adenosine triphosphate (ATP)

A fluorescence titration was conducted using a solution of MagZet1 (20 μM) and ATP (20 mM) in aqueous buffer at pH 7. Increasing concentrations of MgATP spanning from 0 to 50 mM $[Mg^{2+}]_T$ were added from a 0.1 M stock solution, treated with the appropriate amount of MagZet1 to match the concentrations in the cuvette and prevent dilution throughout the experiment.

To determine the apparent affinity of MagZet1 for ATP-bound Mg^{2+}, plots of the fluorescence ratio against $[Mg^{2+}]_T$ were fitted assuming binary and ternary complex formation, in addition to competition between MagZet1 and ATP for free Mg^{2+} using the following equation:

$$R=\frac{S_{f1}K_{d,M}\left(K_{d,T}K_{d,ATP}+K_{d,T}[M]_f\right)+S_{b1}[M]_f\left(K_{d,T}K_{d,ATP}+K_{d,T}[M]_f\right)+S_{T1}K_{d,M}[M]_f[ATP]_T}{S_{f2}K_{d,M}\left(K_{d,T}K_{d,ATP}+K_{d,T}[M]_f\right)+S_{b2}[M]_f\left(K_{d,T}K_{d,ATP}+K_{d,T}[M]_f\right)+S_{T2}K_{d,M}[M]_f[ATP]_T} \qquad \text{(eq S4)}$$

Where R represents the ratio of fluorescence intensity at the metal bound (λ_1 = 530 nm) and metal free (λ_2 = 500 nm) wavelengths excited at 390 nm ($R=I_{\lambda1}/I_{\lambda2}$); S_{f1}, S_{b1}, and S_{T1} are proportionality coefficients for the fluorescence at λ_1 of the free sensor, bound sensor, and ternary complex, respectively; and S_{f2}, S_{b2}, and S_{T2} are proportionality coefficients for the fluorescence at λ_2 of the free sensor, bound sensor, and ternary complex, respectively. $K_{d,M}$ corresponds to the dissociation constant of MagZet1 for Mg^{2+}, $K_{d,ATP}$ corresponds to the affinity of ATP for Mg^{2+}, and $K_{d,T}$ corresponds to the dissociation constant of the ternary complex between MagZet1, ATP, and Mg^{2+}. $[ATP]_T$ represents the total concentration of ATP present in the cuvette, and $[M]_f$ is the concentration of free Mg^{2+} calculated from the solution to the cubic equation:

$$[M]_f{}^3+(K_{d,A}+K_{d,M}+[L]_T+[ATP]_T-[M]_T)[M]_f{}^2+(K_{d,A}K_{d,M}+K_{d,M}A_T+K_{d,A}[L]_T-K_{d,A}[M]_T-K_{d,M}[M]_T)[M]_f-K_{d,A}K_{d,M}[M]_T = 0 \quad \text{(eq S5)}$$

Where $[M]_f$ is the concentration of free Mg^{2+}, $K_{d,A}$ corresponds to the dissociation constant of ATP for Mg^{2+}, $K_{d,M}$ is the dissociation constant of MagZet1 for Mg^{2+}, $[L]_T$ corresponds to the total concentration of MagZet1 in the cuvette, $[M]_T$ is the total concentration of Mg^{2+} in the cuvette, and $[ATP]_T$ is the total concentration of ATP in the cuvette.

Determination of the acid dissociation constant (K_a)

A fluorescence titration of a solution of MagZet1 (20 μM) in aqueous buffer (50 mM MES, 100 mM KCl) was conducted as the pH of the solution was adjusted from pH 3 to pH 11 by the addition of small volumes of 37% HCl or 1 M KOH. The fluorescence ratio as a function of pH was then fitted according to eq S6 to obtain the acid dissociation constant (K_a):

$$R=\frac{R_a+10^{pH-pK_a}\frac{S_{b2}}{S_{a2}}R_b}{10^{pH-pK_a}\frac{S_{b2}}{S_{a2}}+1} \quad \text{(eq S6)}$$

where R represents the ratio of fluorescence intensity at the metal bound (λ_1 = 530 nm) and metal free (λ_2 = 500 nm) wavelengths when excited at 390 nm ($R=I_{\lambda1}/I_{\lambda2}$), R_a is the fluorescence ratio of the protonated sensor at low pH, R_b is the fluorescence ratio of the deprotonated sensor at high pH, and S_{b2} and S_{a2} are proportionality coefficients for the fluorescence at λ_2 of the deprotonated and protonated forms of the sensor, respectively.

Plots of the absorbance and fluorescence at a single wavelength against pH were also fitted according to eq S7, where I corresponds to the fluorescence intensity or absorbance, I_a is the fluorescence intensity or absorbance of the protonated sensor in acidic pH, and I_b is the fluorescence intensity or absorbance of the deprotonated sensor in alkaline solution.

$$I=\frac{I_A+(I_B-I_A)10^{-pH}}{K_a+10^{-pH}} \quad \text{(eq S7)}$$

Study of pH-dependence of the response to Mg^{2+}

The pH-dependence of the fluorescence response to Mg^{2+} was examined using 5 μM solutions of MagZet1 in aqueous buffer at pH values ranging from 5.0 to 8.0. Aqueous MES buffers (50 mM MES, 100 mM KCl) was used for pH values between 5.0 to 6.5 and aqueous HEPES buffers (50 mM HEPES, 100 mM KCl) was used for values between 7.0 and 8.0. Solutions of sensor at each pH were prepared containing no $MgCl_2$, 1 mM $MgCl_2$, or 50 mM $MgCl_2$. Emission spectra were then recorded upon excitation at 390 nm and a ratio was calculated using the fluorescence intensity at 500 nm and 530 nm. Fluorescence ratios were plotted against the pH of solution. Values represent the average of three independent measurements with the error representing the standard deviation.

2.3 Computational details

DFT calculations on all molecules were carried out using the Gaussian 16 program package[8] applying both a tightened self-consistent field convergence criterion (10^{-9}-10^{-10} au) and an improved optimization threshold (10^{-5} au on average forces). Ground state geometry optimized structures of MagZet1 complexed with Ca^{2+} and Mg^{2+} were obtained and the corresponding vibrational spectra were computed and analyzed to verify the conversion to a minimum by the absence of imaginary frequencies. All calculations used the same DFT integration grid, the ultrafine pruned (99590) grid, as well as the M06-2X hybrid exchange-correlation functional,[9] which has been shown to be a good choice for investigating structures and excited states push-pull type dyes.[10-13] Structural parameters were obtained with the extended 6-311+G(d,p) atomic basis set. Bulk solvation effects of the aqueous medium were estimated using the polarizable continuum model (PCM)[14] that was systematically applied to all computational steps. In all calculations, explicit water molecules were added to complete the metal coordination sphere (Mg, pseudo-

octahedral; Ca, 8-coordinate) as is common for these metals and observed in crystal structures. Optimized structures were visualized with and graphics were generated using the Mercury software package.[15]

Coordinates of the optimized MagZet1-Mg^{2+} complex

Atoms			
C	-2.01411297	-0.45021574	0.42533056
C	-2.11177581	-1.86877591	0.38691158
C	-0.96058333	-2.61373073	0.21255515
C	0.28252805	-1.96657869	0.08013794
C	0.37941955	-0.53766777	0.10266814
C	-0.82842269	0.22045475	0.28782526
C	1.50283551	-2.67996312	-0.08413129
C	2.69707588	-2.03328770	-0.21357475
C	2.68425654	-0.62881945	-0.18663773
N	1.58305711	0.07830881	-0.03639656
N	-3.33254677	-2.45952182	0.55501351
C	-4.63304909	-1.88575929	0.16709373
C	-3.69490968	-3.80220117	0.08025955
C	-0.92703122	1.75527021	0.35707309
O	-2.00004389	2.22788463	0.73147450
O	0.07989832	2.45014057	0.00962964
C	3.99038776	0.15536469	-0.34167551
O	3.86039027	1.42135049	-0.34328725
O	5.03505398	-0.48142147	-0.45068155
Mg	2.01525967	2.19263450	-0.17900703
C	-5.16153399	-3.32643375	-0.01881915
H	-4.57306316	-1.31871720	-0.76808731
H	-5.10870537	-1.27764444	0.93725394
H	-5.66822537	-3.53143779	-0.95779365
H	-5.77168043	-3.66261159	0.81685890
H	-3.47562146	-4.60719567	0.78309051
H	-3.24139085	-4.02891879	-0.89192567
Cl	1.48686331	-4.41861671	-0.12182392
O	2.31400753	2.52871039	1.86517185
H	3.07549869	2.14749375	2.31534618
O	1.80697746	2.29800221	-2.26262042
H	0.92832977	2.14869520	-2.62856879
O	2.57127891	4.17059807	-0.36827668
H	2.53843018	4.77259047	0.38229799
H	2.33419567	4.67090385	-1.15596021
H	2.43828591	1.88877854	-2.86379783
H	1.57740092	2.50448605	2.48517369
H	3.63290715	-2.55942287	-0.33706937
H	-1.00929520	-3.69408759	0.18955639
H	-2.91148751	0.13670082	0.57762257

Coordinates of the optimized MagZet1-Ca^{2+} complex

Atoms			
C	-2.08063362	-0.37338244	0.26295835
C	-2.17202154	-1.77602619	0.48880926
C	-1.01980327	-2.53898816	0.42437934
C	0.22075677	-1.93305666	0.14345052
C	0.32516670	-0.52275817	-0.06563949
C	-0.88450623	0.24014359	0.01831420
C	1.43237480	-2.66707726	0.03219142
C	2.60537954	-2.04456040	-0.26742329
C	2.59393763	-0.64757327	-0.47068777
N	1.50506446	0.08937976	-0.37854611
N	-3.38636429	-2.32971789	0.78622319
C	-4.69445281	-1.83854651	0.31796986
C	-3.74923649	-3.73657644	0.56363503
C	-0.89176512	1.74461834	-0.14340350
O	-1.73686055	2.25069127	-0.89818203
O	-0.01808383	2.38752598	0.51265482
C	3.92805498	0.02740378	-0.81109286
O	3.89483212	1.28912472	-0.94537596
O	4.91597376	-0.69750158	-0.92526807
Ca	2.07404323	2.72033556	-0.61265466
C	-5.21967684	-3.28949396	0.40630207
H	-4.64983169	-1.45344066	-0.70656834
H	-5.16162106	-1.09944307	0.96972164
H	-5.74226113	-3.66258720	-0.47014554
H	-5.81306426	-3.46865563	1.30044452
H	-3.51365342	-4.40355725	1.39409586
H	-3.31097903	-4.13100910	-0.36080177
Cl	1.42711358	-4.39104895	0.27239128
O	2.33506285	2.14891222	1.77897294
H	2.91586888	1.46144289	2.11852655
O	3.02821887	2.94224997	-2.86506570
H	3.30685455	3.69288263	-3.39764700
O	1.45946313	4.70380381	0.70352251
H	1.99680884	4.65630320	1.50251490
H	0.59753211	4.33662649	0.95053295
H	3.80323629	2.37756431	-2.73898613
H	1.42880337	1.80962740	1.83940458
H	3.53749174	-2.58305763	-0.35901596
H	-1.06768041	-3.60737830	0.58777000
H	-2.98147075	0.22932934	0.29925659
O	0.35201758	3.65401037	-2.03668842
H	-0.54823460	3.37627640	-1.78045033
O	4.19812195	3.79362989	-0.05301725
H	4.76405200	3.05386851	-0.31794824
H	0.41218407	3.57702358	-2.99342330
H	4.59585752	4.59318332	-0.40956111

2.4 Cell culture

HeLa cells were grown in a culture system containing Dulbecco's Modified Eagle Medium (DMEM) supplemented with 10% fetal bovine serum (FBS) at 37 °C in a 5% CO_2 atmosphere according to ATCC guidelines. Cells were subcultured or the medium was changed twice a week. THLE-2 cells were cultured in a system containing Bronchial Epithelial Cell Basal Medium (BMEM) supplemented with growth factor BulletKit (Lonza) from which gentamycin/amphotericin (GA) and epinephrine are discarded. The medium is then further supplemented with epidermal growth factor (EGF, 5 ng/mL), phosphoethanolamine (70 ng/mL), 10% FBS, 1% glutamine, and 1% Penicillin and 1% Streptomycin. THLE-2 cells were grown on plates precoated with a mixture of 0.01 mg/mL fibronectin, 0.01 mg/mL bovine serum albumin, and 0.03 mg/mL collagen. Cells were sub-cultured or the medium was changed twice a week.

2.5 Imaging

2.5.1 General imaging details

Confocal images were obtained using a 63× oil immersion objective (NA = 1.4) in a Zeiss 880 inverted laser scanning confocal microscope equipped with a Zeiss AxioObserver inverted stand, environmental chamber for temperature and stage insert for CO_2 control, and Definite Focus autofocusing system; or in a Leica TCS SP8 confocal laser scanning microscope with GaAsP hybrid detectors and OkoLab stage-top environmental chamber. A 405 nm excitation laser line was used and emission was collected from 470 to 520 for the free form and 520 to 582 for the metal bound sensor. Image processing and ratio measurements were conducted using ImageJ.[16] For fluorescence ratio images, background correction and thresholding was applied to individual images prior to ratio calculation.

2.5.2 Imaging free Mg^{2+} in HeLa cells with MagZet1AM

HeLa cells, grown as described above, were plated on 3.5 cm glass bottom imaging dishes (MatTek). Cells were grown to ~80% confluency prior to imaging. Cells were washed with warm DMEM without FBS (1×2 mL) and then bathed in DMEM without FBS, containing 5 μM MagZet1AM added from a stock solution of the sensor suspended in 20% Pluronic F127 according to manufacturer protocol (final concentration of Pluronic in the plate was 0.01%). Sensor loading was conducted for 30 min at room temperature, protected from light. After this period, the medium was replaced with fresh DMEM without FBS (2 mL) and the cells were left at room temperature protected from light for an additional 30 min to allow for complete de-esterification of the sensor. The cells were then washed with HBSS without divalent cations (1×2 mL) and were then bathed in HBSS without cations (1 mL). To confirm the sensors response to Mg^{2+}, cells on the microscope stage were supplemented with $MgCl_2$ and the nonfluorescent ionophore 4-Br-A23187 (Molecular Probes) for a total concentration of 50 mM and 10 μM, respectively. Additionally, cells on the microscope stage were supplemented with an aqueous solution of EDTA free acid and the nonfluorescent ionophore 4-Br-A23187 (Molecular Probes) for a total concentration of 50 mM and 10 μM, respectively, to deplete Mg^{2+} levels. Images were collected immediately before treatment with Mg^{2+} (or EDTA) and every 5 min afterward for 1 h. For comparison, images were acquired and processed under identical conditions.

2.5.3 Control experiments to rule out detection of Ca^{2+} or Zn^{2+}

To test for possible interference from other cations in cellulo, HeLa cells, loaded with MagZet1AM as described above and bathed with HBSS without metal cations, were treated on the microscope stage with Ca^{2+}-selective chelator BAPTA-AM for a final concentration of 10 μM. Images were collected every 5 min for 30 min. To rule out interference from Zn^{2+}, cells on the microscope stage were treated with Zn^{2+}-selective chelator TPA for a final concentration of 10 μM and imaged 5 min after treatment. For comparison, images were acquired and processed under identical conditions.

2.5.4 Imaging free Mg^{2+} in paracetamol-treated THLE-2 cells with MagZet1AM

THLE-2 cells, cultured as described above, were plated on 3.5 cm glass bottom imaging dishes (MatTeK) and were grown to ~80% confluency prior to treatment and imaging. Paracetamol (Sigma-Aldrich) was dissolved in DMSO to prepare 1 M stock solution. Cells were treated at a dose of 10 mM in growth medium for 1, 3, or 6 h. A vehicle control consisting of DMSO in growth medium was used for comparison and normalization. After the designated treatment times, cells were washed with imaging medium (DMEM, no phenol red) without FBS. Cells were then bathed in fresh imaging medium without FBS containing 5 μM MagZet1AM added from a stock solution of the sensor suspended in 20% Pluronic F127 according to manufacturer protocol (final concentration of Pluronic in the plate was 0.01%). Sensor loading was conducted for 30 min at room temperature, protected from light. After this period, the medium was replaced with fresh DMEM without FBS (2 mL) and the cells were left at room temperature protected from light for an additional 30 min to allow for de-esterification of the sensor. The cells were then washed with DMEM without FBS (1×2 mL), bathed in DMEM without FBS or phenol red and imaged. For comparison, images were acquired and processed under identical conditions.

2.6 Flow cytometry

For flow cytometry experiments, MagZet1 was detected using a BD LSRII flow cytometer with a 407 nm violet excitation laser. Emission from the "free" sensor was collected using a BV421 filter set with a 450/50 bandpass filter and the signal from the "bound" sensor was collected using a BV510 filter set with a 505 nm LP dichroic mirror and a 530/30 nm bandpass filter. Prior to running samples for analysis, a "Mg^{2+} saturated" sample, containing 50 mM $MgCl_2$ and 10 μM the nonfluorescent ionophore 4-Br-A23187 in the medium, and a "Mg^{2+} depleted" sample, containing 100 mM EDTA acid and 10 μM the nonfluorescent ionophore 4-Br-A23187 in the medium, were used to determine the full dynamic range of the sensor. The fluidics were then purged for several minutes with 70% ethanol and sheath to remove traces of ionophore.

HeLa cells or THLE-2 cells were grown on 10 cm dishes, trypsinized, and collected by centrifugation. Cell pellets were then resuspended in DMEM without FBS or phenol red to a final concentration of ~1×10^6 cells/mL. Cells were stained in suspension using medium containing 5 μM MagZet1AM added from a stock solution of the sensor suspended in 20% Pluronic F127 according to manufacturer protocol (final concentration of Pluronic in solution was 0.01%) for 30 min at room temperature protected from light. After this period, cells were collected by centrifugation, resuspended in DMEM without FBS or phenol red, and left at room temperature protected from light for an additional 30 min to allow for de-esterification of the sensor. Cells were then pelleted and resuspended in DMEM without phenol red and FBS. Samples were aliquoted and kept on ice. Cell suspensions were gently vortexed and warmed to room temperature immediately prior to data acquisition. FCS files were exported and analyzed in FlowJo v10.9. Bivariate scatterplots were analyzed and gated according to the following scheme: time gate for cleaning > FSC v. SSC to gate out debris > FSC-H v FSC-A to gate out doublets > SSC v. BV421 or BV510 for mean fluorescence intensities (MFIs) for the metal-bound and metal-free forms of the sensor, respectively. Fluorescence minus one (FMO) samples were used to set gates for "stained" cells.

2.6.1 Statistical analysis

Changes in fluorescence ratio upon treatment were analyzed using the population comparison feature in FlowJo v10.9, which is based on a Cox Chi Squared approach[17] but using probability binning. Histograms of fluorescence ratio (510nm/421nm) of each sample was compared with a control, vehicle-treated sample analyzed and processed using the same protocol and parameters. Chi-squared values from pair-wise population comparisons were used to calculate *p*-values reported.

2.7 Analysis of transporter expression in paracetamol-treated THLE-2 cells

2.7.1 RNA isolation and first strand cDNA generation

THLE-2 cells were grown in 3.5 cm dishes to approximately 80% confluency and then were treated with paracetamol as described above. Cells were washed with DPBS (1×2mL) and total RNA was extracted using the PureLink RNA mini kit (Invitrogen) following the manufacturer protocol. RNA was treated with DNase (Agilent) to degrade genomic DNA at 37 °C for 1 h and then with 5 mM EDTA at 75 °C for 10 min. Total RNA concentration was measured by nanodrop (DeNovix DS-11+). To synthetize cDNA, reverse transcription was performed using M-MLV (Moloney-Murine Leukemia Virus) reverse transcriptase (Invitrogen) added to 1.0 µg of total RNA for 50 min at 37 °C. Samples from reverse transcription were then diluted 1:10, aliquoted, and stored at -20 °C.

2.7.2 Quantitative Real-Time Polymerase Chain Reaction (RT-qPCR)

Samples consisting of ~10 ng template cDNA, forward and reverse primers (400-800 nM), 10 µL PowerUp^{TM} SYBR Green master mix (ThermoFisher), and 7 µL nuclease free water were used to analyze gene expression levels by RT-qPCR on a LightCycler 480 (Roche). Nontemplate control (NTC) samples exchanging water for cDNA and control samples not subjected to reverse transcription (NRT) were also conducted. Experiments were carried out at 50 °C for 3 min and 95 °C for 5 min, followed by 75 cycles of 15 s at 95 °C and 30 s at 55 °C, followed by a melt curve analysis. After normalizing to housekeeping gene expression (ARP), the relative mRNA expression was calculated by the Livak method ($2^{-\Delta\Delta Ct}$). Reported values represent the average of three biological replicates and were plotted as heatmaps using R(v 4.1.1).[18] Primers used are listed in Table S1.

Table S1. Primer sequences used for quantitative real time PCR.

Gene	Species	Forward Primer (5’→3’)	Reverse Primer (5’→3’)
ARP	Human	CGACCTGGAAGTCCAACTAC	ATCTGCTGCATCTGCTTG
CNNM1	Human	TAA GCACCTTCTACACGCGG	CAGCACCTCCTCCACAACTT
CNNM2	Human	ATGATGATGACCTTCCCCGC	CGAGGTCGTTGTAGGGATCG

CNNM3	Human	TCCTGTCCCGAGAAGTGGAT	CTGGTTGACACTGGGATGCT
CNNM4	Human	GAGCTGCAACAACTCGTGTG	TCCACCTCGGTGAAGGAGAT
MagT1	Human	CCTAAGGACAGTTTGATGGGACA	ACAACATTAAGAATGAGGGACCA
MMgT1	Human	CGCTTTCGCCTCTTCGTTTA	GAGGGCTGCGCTATGGAG
Mrs2	Human	AGAGCAGTGCTGGGATTGAC	GAGATCGTCAGCCAATCGGT
TRPM7	Human	GTTGGAAAGTATGGGGCGGA	ACATTTGGCCCATCAAA
TRPM6	Human	AGTTCTCTGGTCTCCACCA	TGGCAGACTTGGCATACTGG

2.7.3 Protein isolation and Western blotting

THLE-2 cells were grown in 10 cm dishes to approximately 80% confluency. Cells were then treated with paracetamol as described above. Cells were then trypsinized and collected by centrifugation. The cell pellet was stored at -80 °C until the cells were lysed by sonication in RIPA lysis buffer (50 mM Tris-HCl pH 7.4, 150 mM NaCl, 1% NP-40, 0.25% Na-deoxycholate) containing 2 mM of the protease inhibitor phenylmethylsulfonyl fluoride (PMSF). Total protein content was determined by Bradford assay (BioRad). Cell lysate (~15 µg/lane) was resolved in sodium dodecyl sulfate-polyacrylamide gels and transferred to nitrocellulose membranes. After blocking with 5% nonfat milk, the membrane was incubated at 4 °C overnight with primary Rab-AntiCNNM4 (Abcam AB191207, 1:1000) and mouse-Anti-GAPDH (Abcam AB8245, 1:10000) and then with anti-Mouse (Cell Signaling Technologies 7076S, 1:5000) and antiRabbit (Cell Signaling Technologies 7074S, 1:1000) peroxidase conjugated secondary antibodies at room temperature for 1 h. The reactive proteins were visualized with Clarity^{TM} Western ECL substrate (Bio-Rad) and images were captured with a ChemiDoc MP Imaging System (Bio-Rad).

3 Spectroscopic data for new compounds

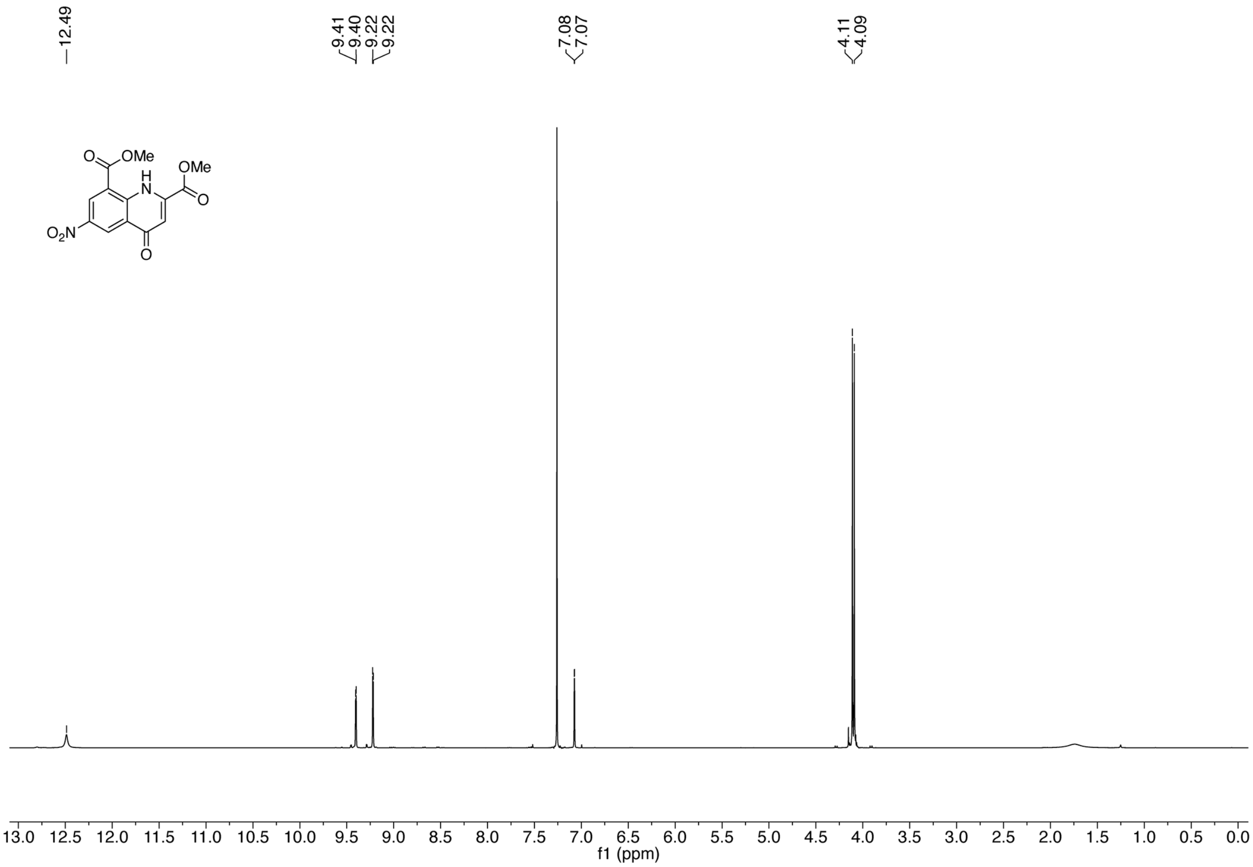

Figure S21. ^{1}H NMR of compound **3** in $CDCl_3$

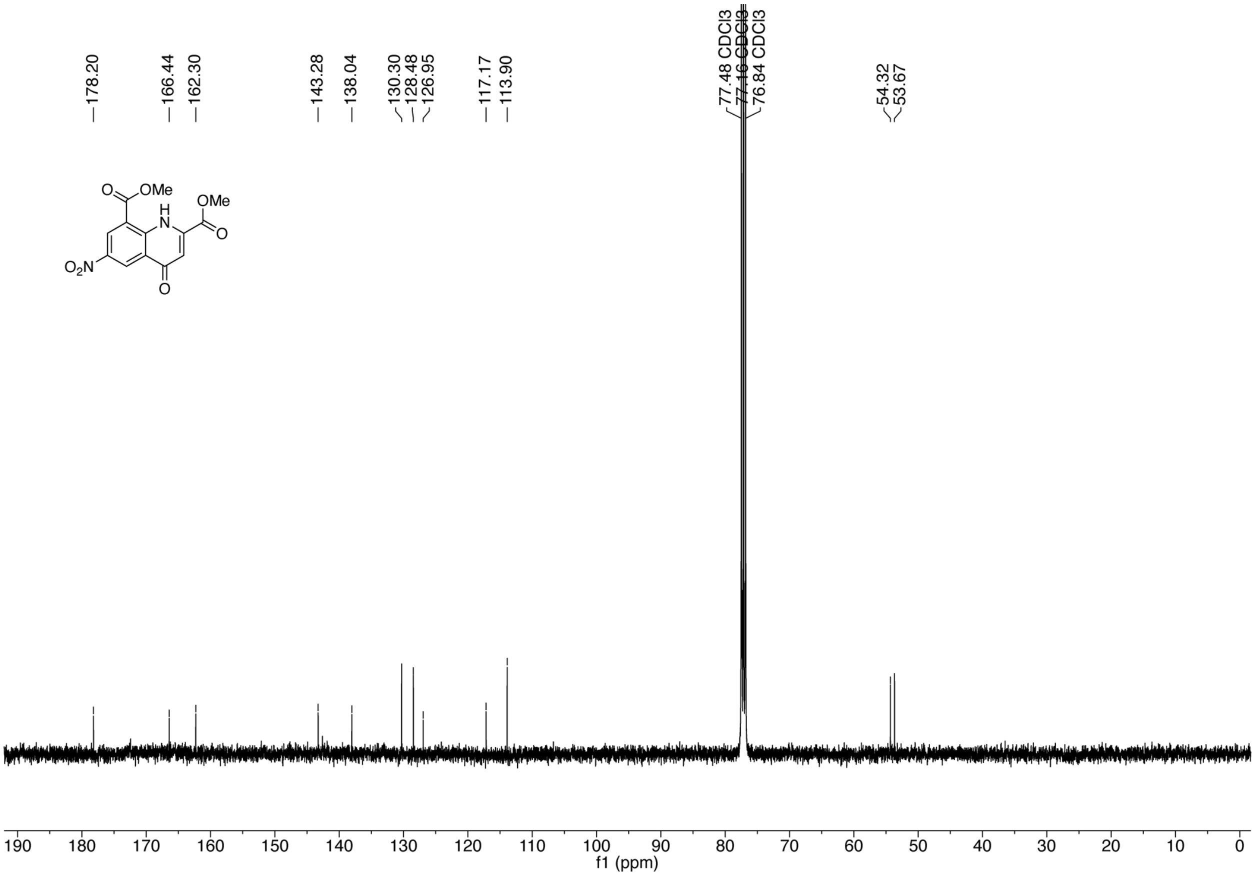

Figure S22. $^{13}C\{^1H\}$ NMR of compound **3** in $CDCl_3$

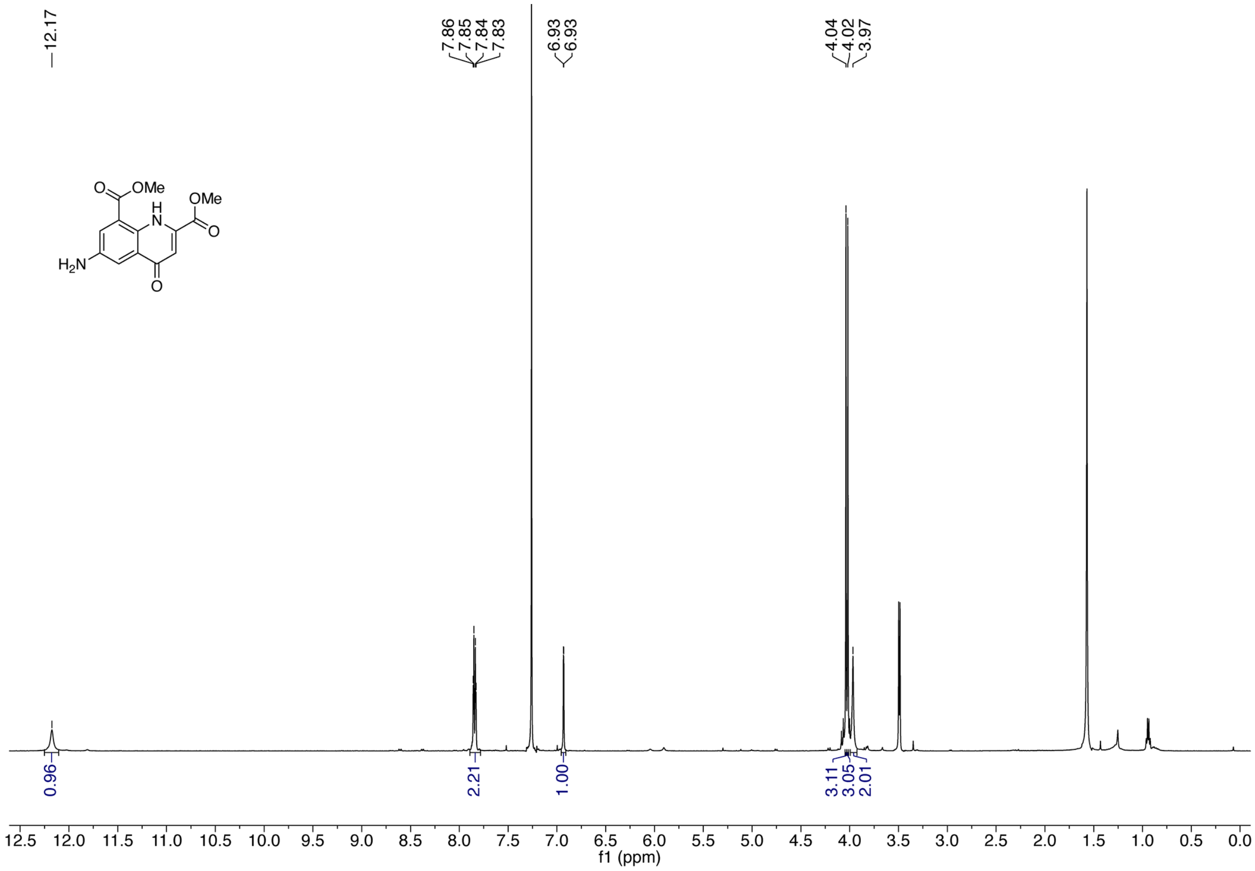

Figure S23. ^{1}H NMR of compound **4** in $CDCl_3$

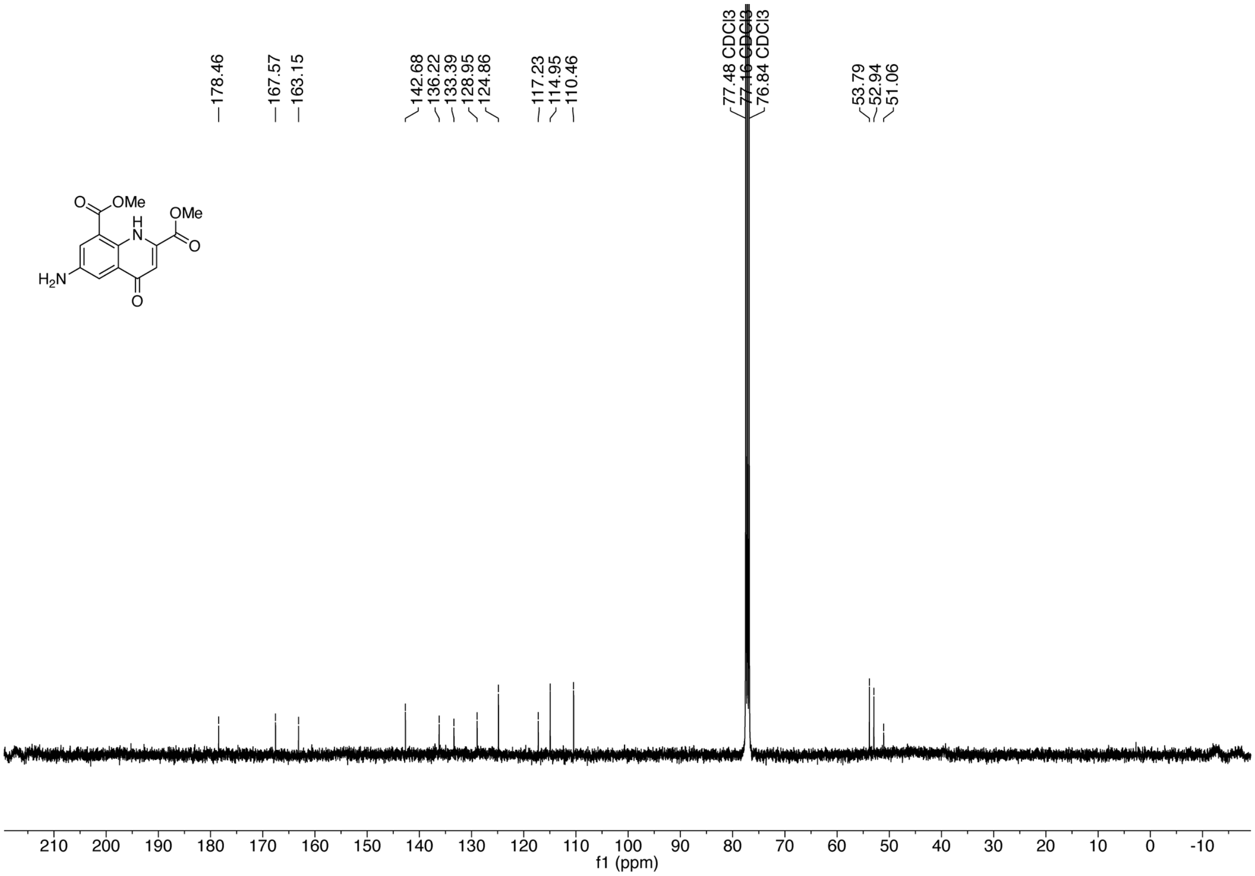

Figure S24. $^{13}C\{^{1}H\}$ NMR of compound **4** in $CDCl_3$

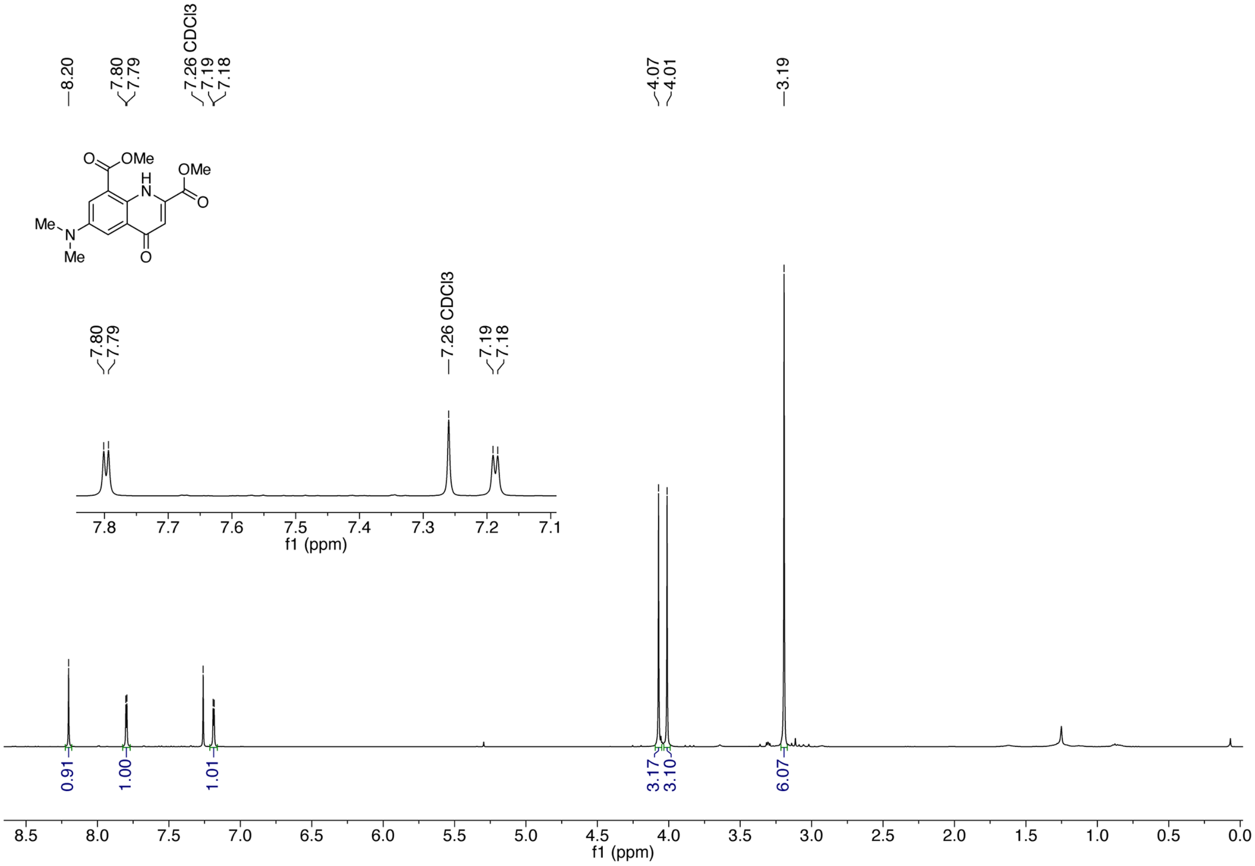

Figure S25. ^{1}H NMR of compound **5** in $CDCl_3$

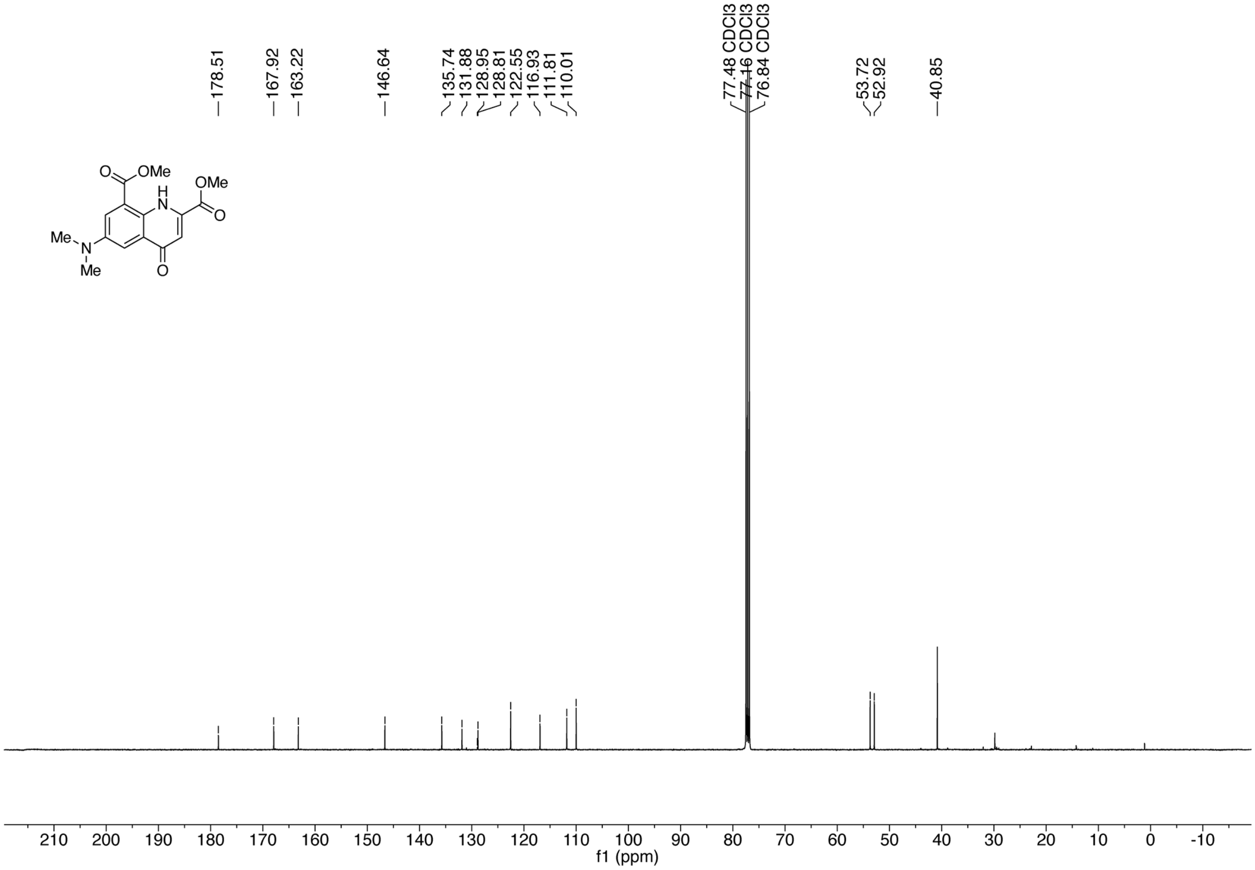

Figure S26. $^{13}C\{^{1}H\}$ NMR of compound **5** in $CDCl_3$

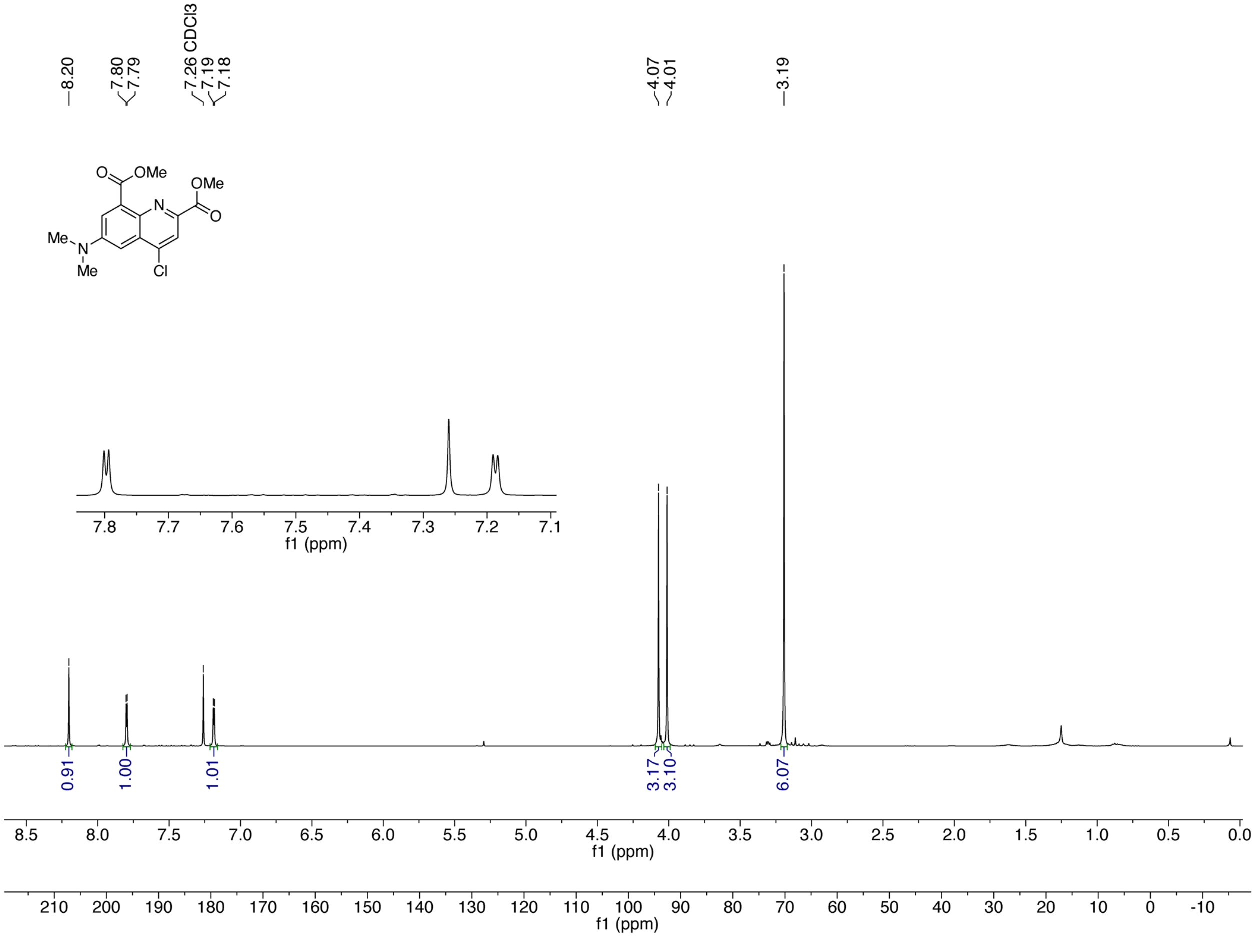

Figure S27. ^{1}H NMR of compound **6** in $CDCl_3$

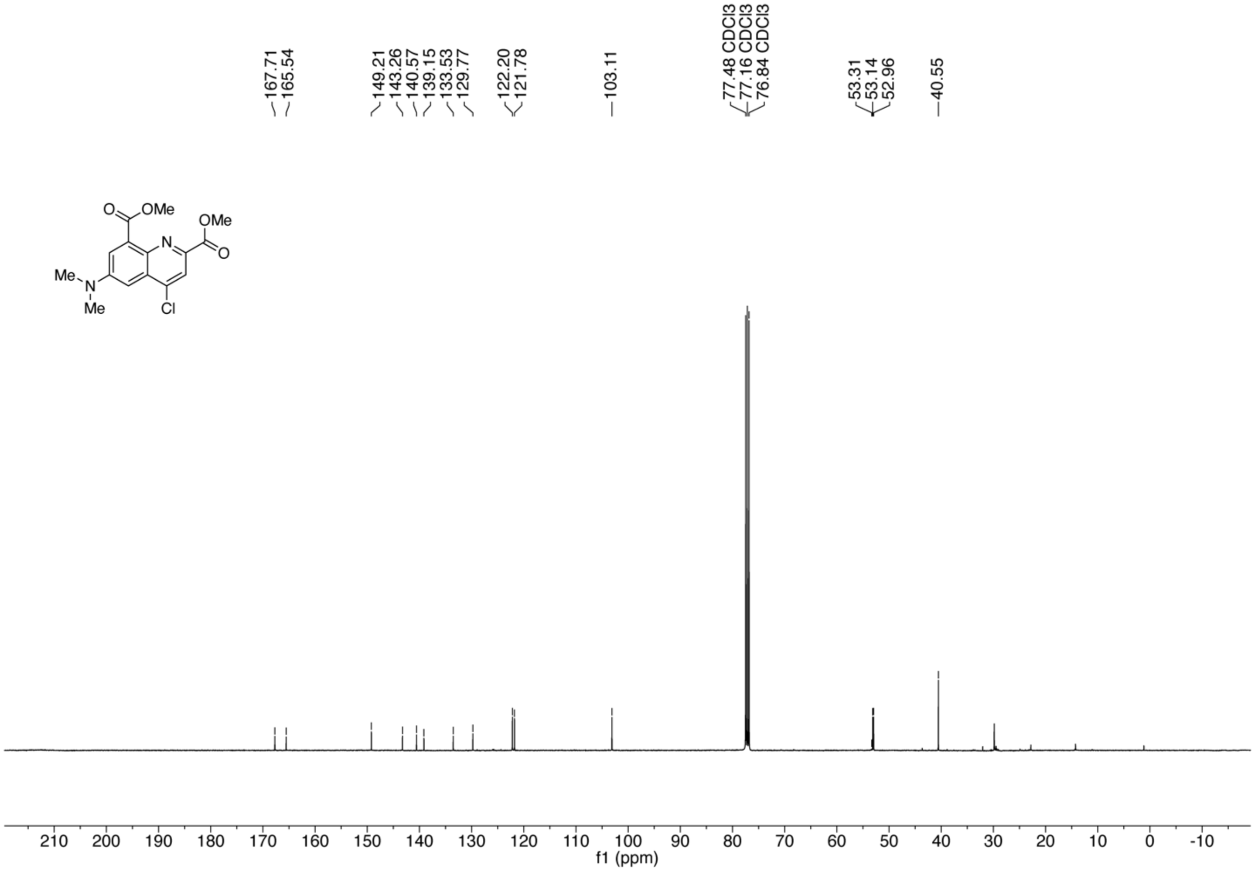

Figure S28. $^{13}C\{^{1}H\}$ NMR of compound **6** in $CDCl_3$

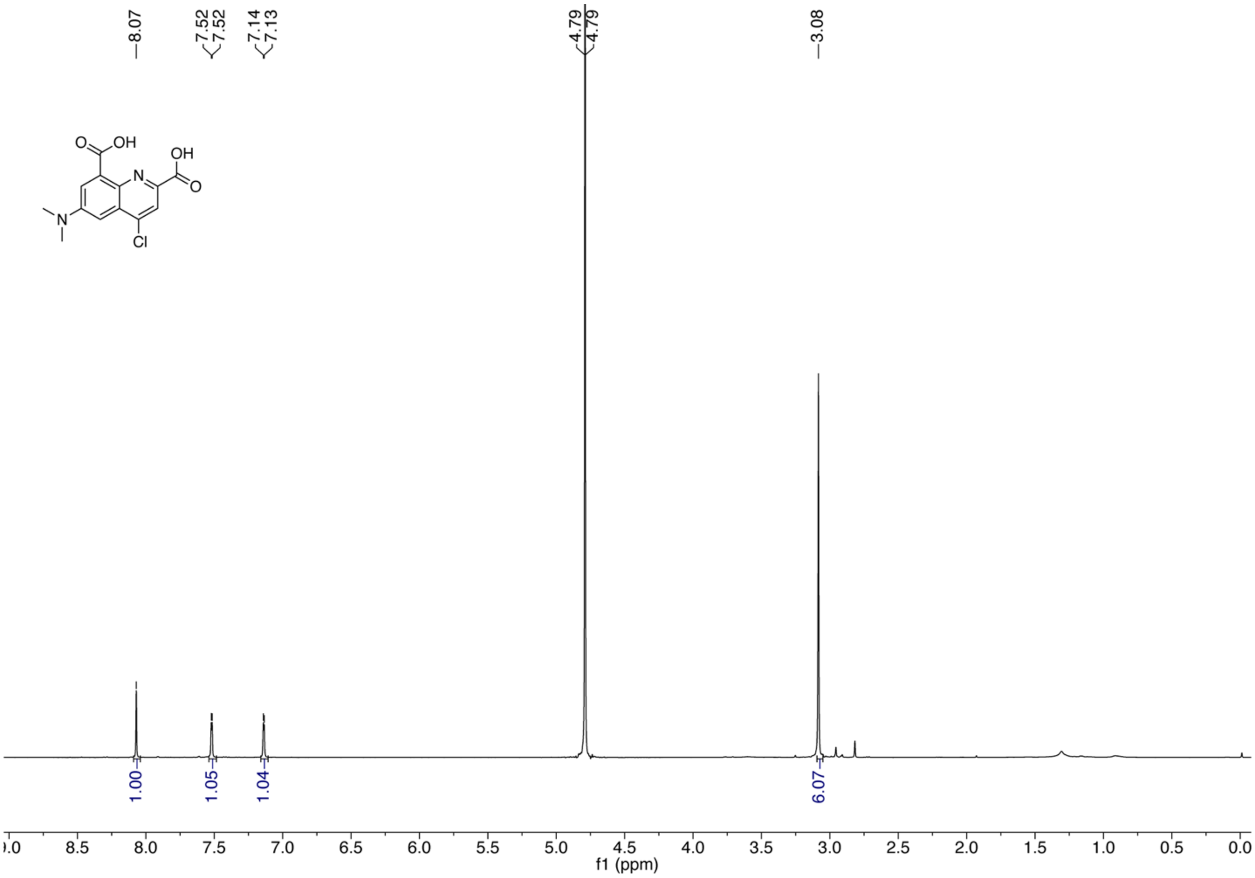

Figure S29. ^{1}H NMR of compound **7** in NaOD.

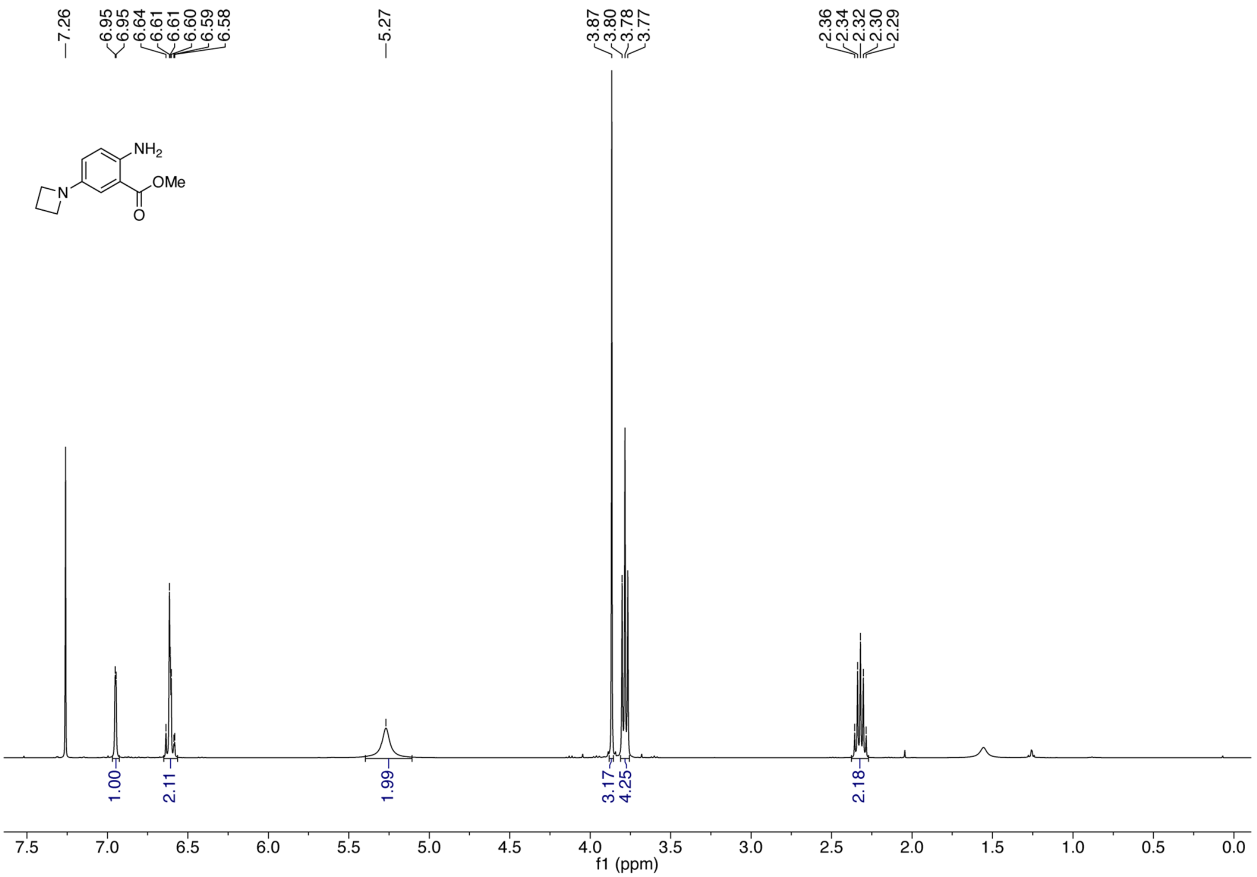

Figure S30. ^{1}H NMR of compound **9** in $CDCl_3$

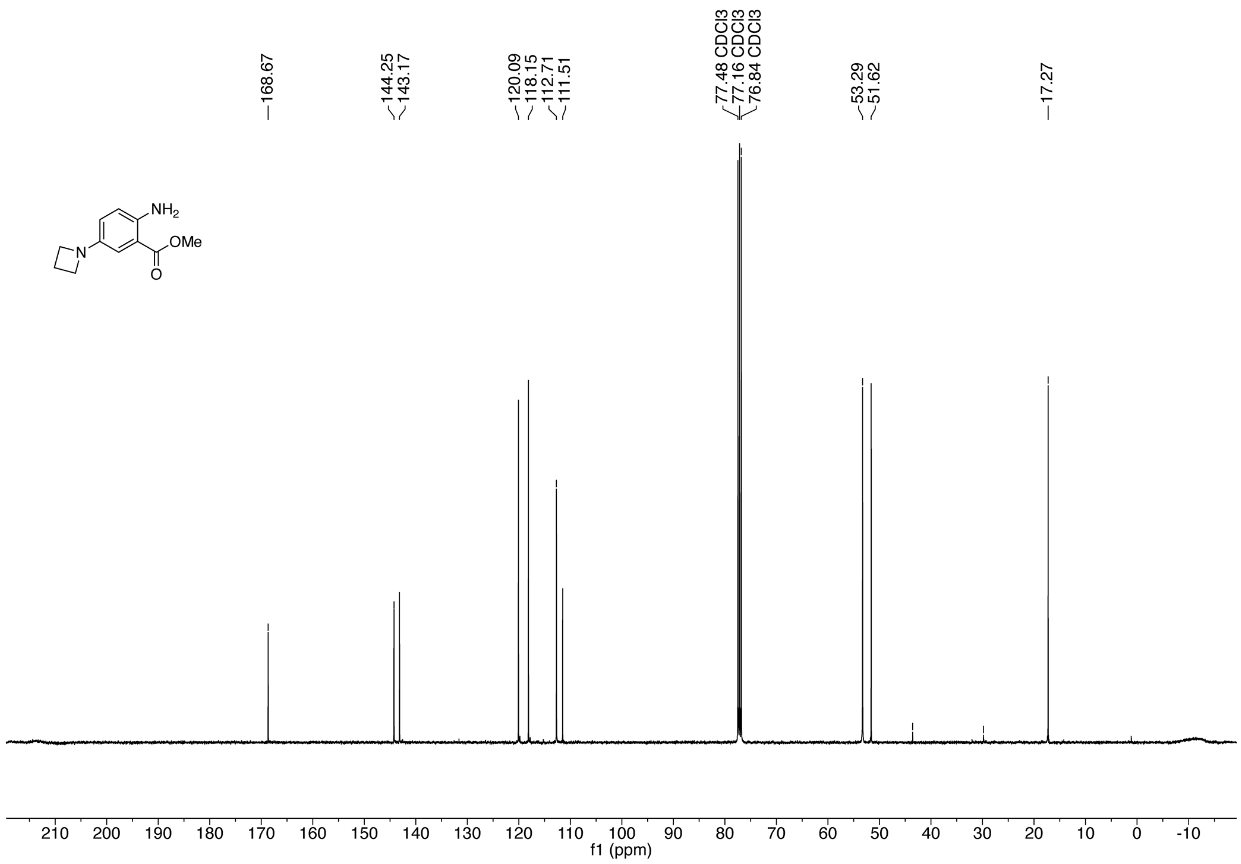

Figure S31. $^{13}C\{^{1}H\}$ NMR of compound **9** in $CDCl_3$

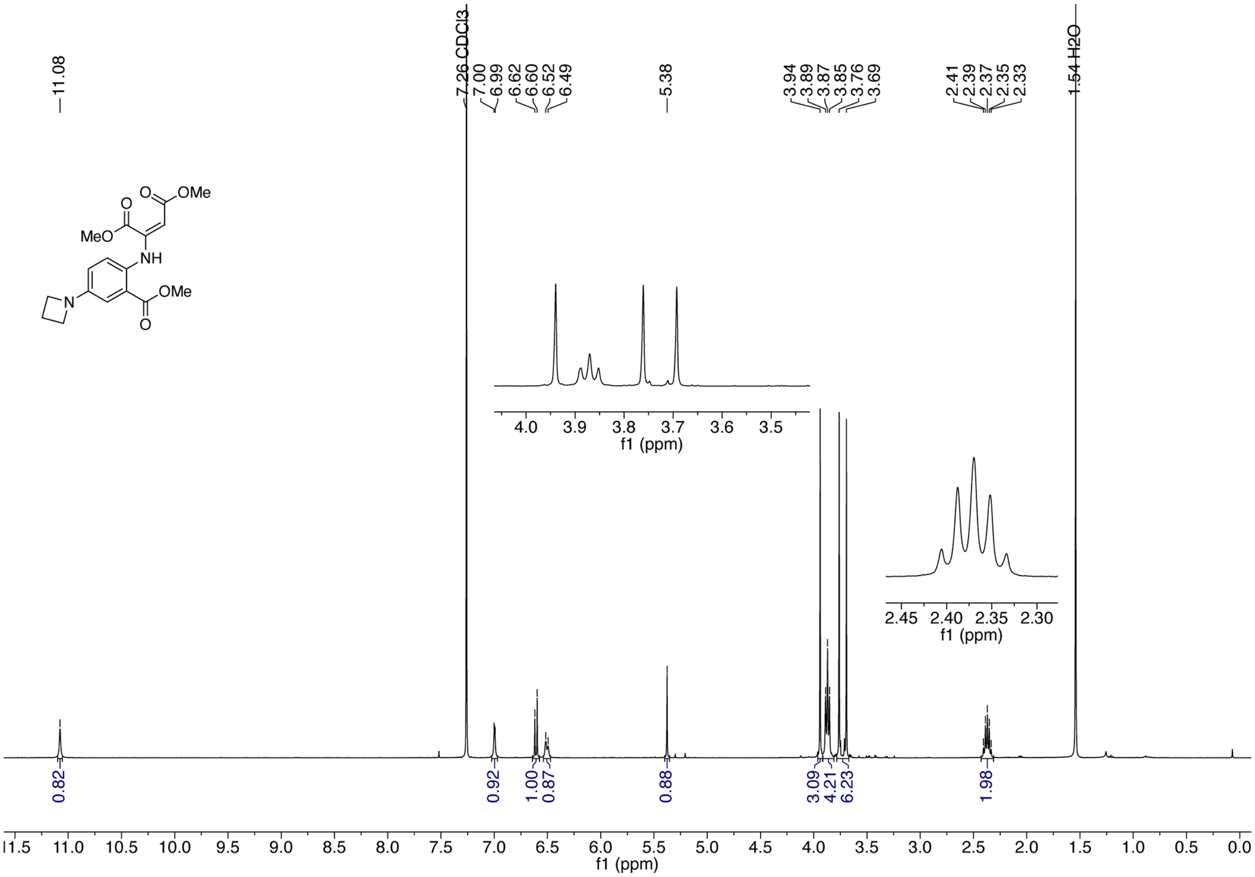

Figure S32. ^{1}H NMR of compound **10** in $CDCl_3$

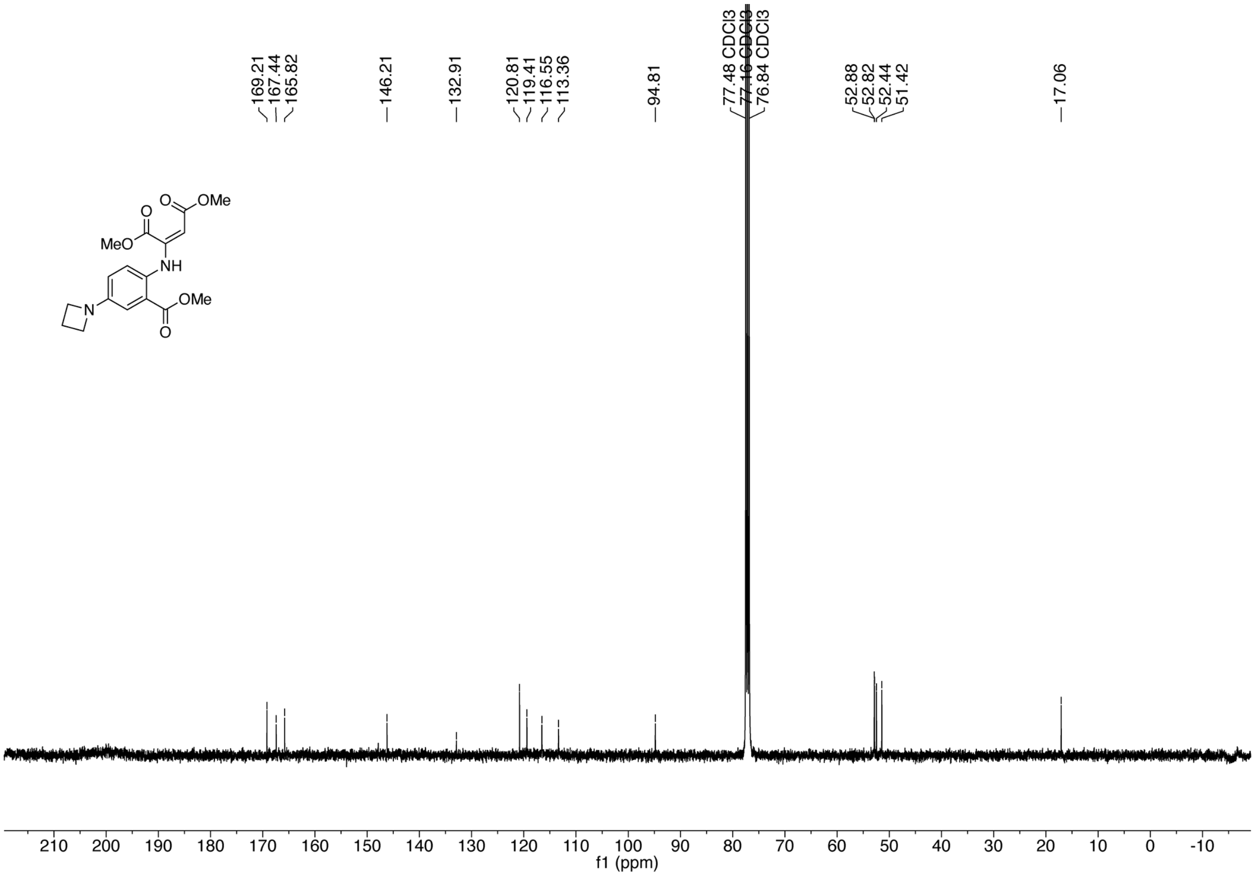

Figure S33. $^{13}C\{^{1}H\}$ NMR of compound **10** in $CDCl_3$

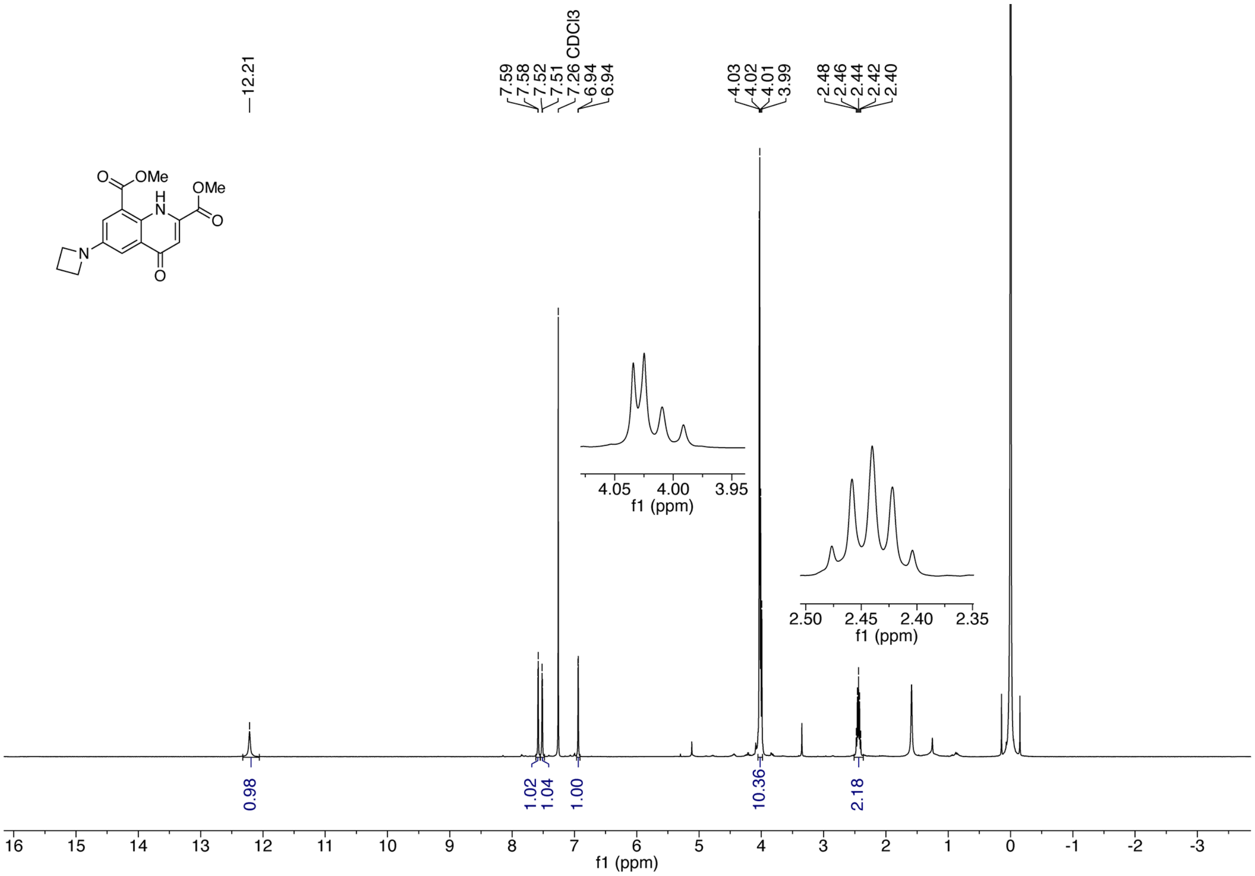

Figure S34. ^{1}H NMR of compound **11** in $CDCl_3$

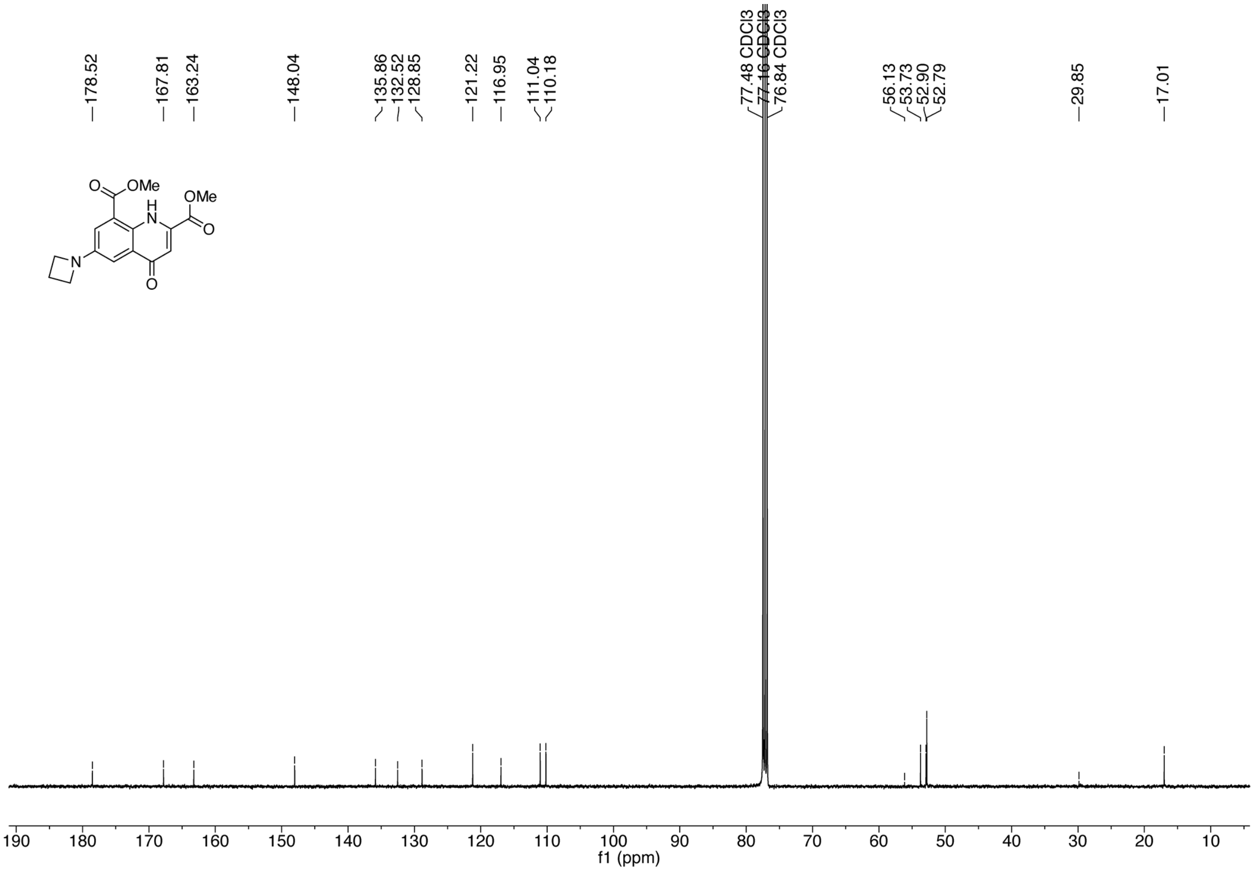

Figure S35. $^{13}C\{^{1}H\}$ NMR of compound **11** in $CDCl_3$

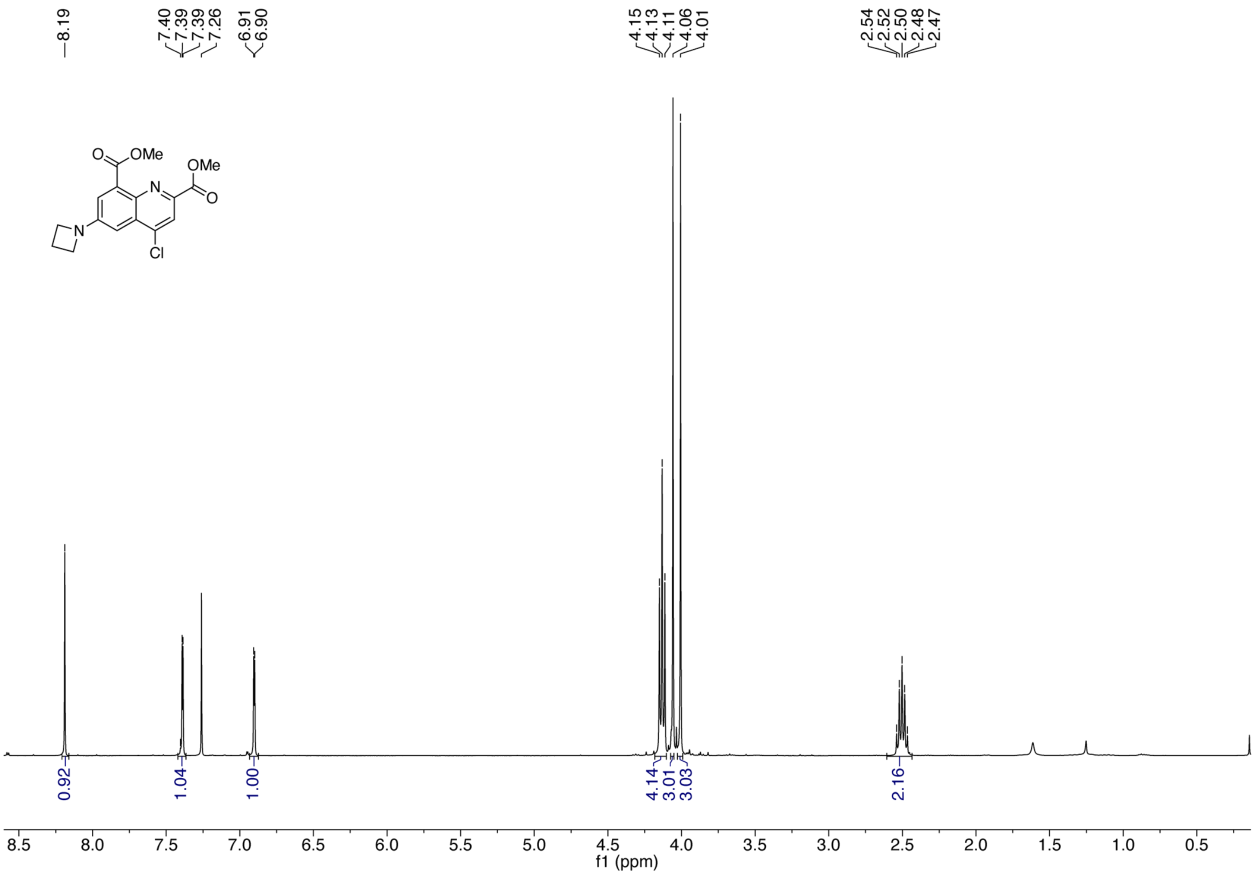

Figure S36. ^{1}H NMR of compound **12** in $CDCl_3$

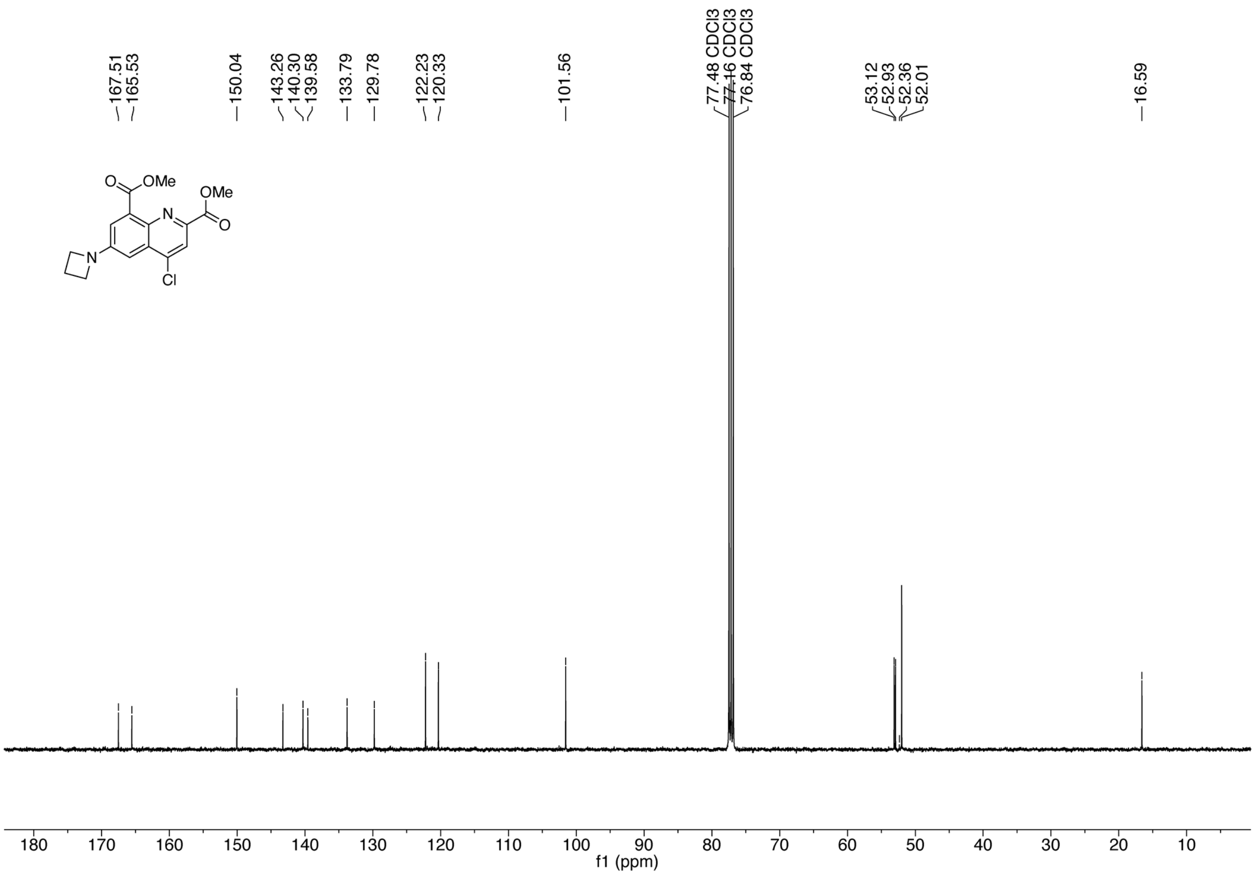

Figure S37. $^{13}C\{^{1}H\}$ NMR of compound **12** in $CDCl_3$

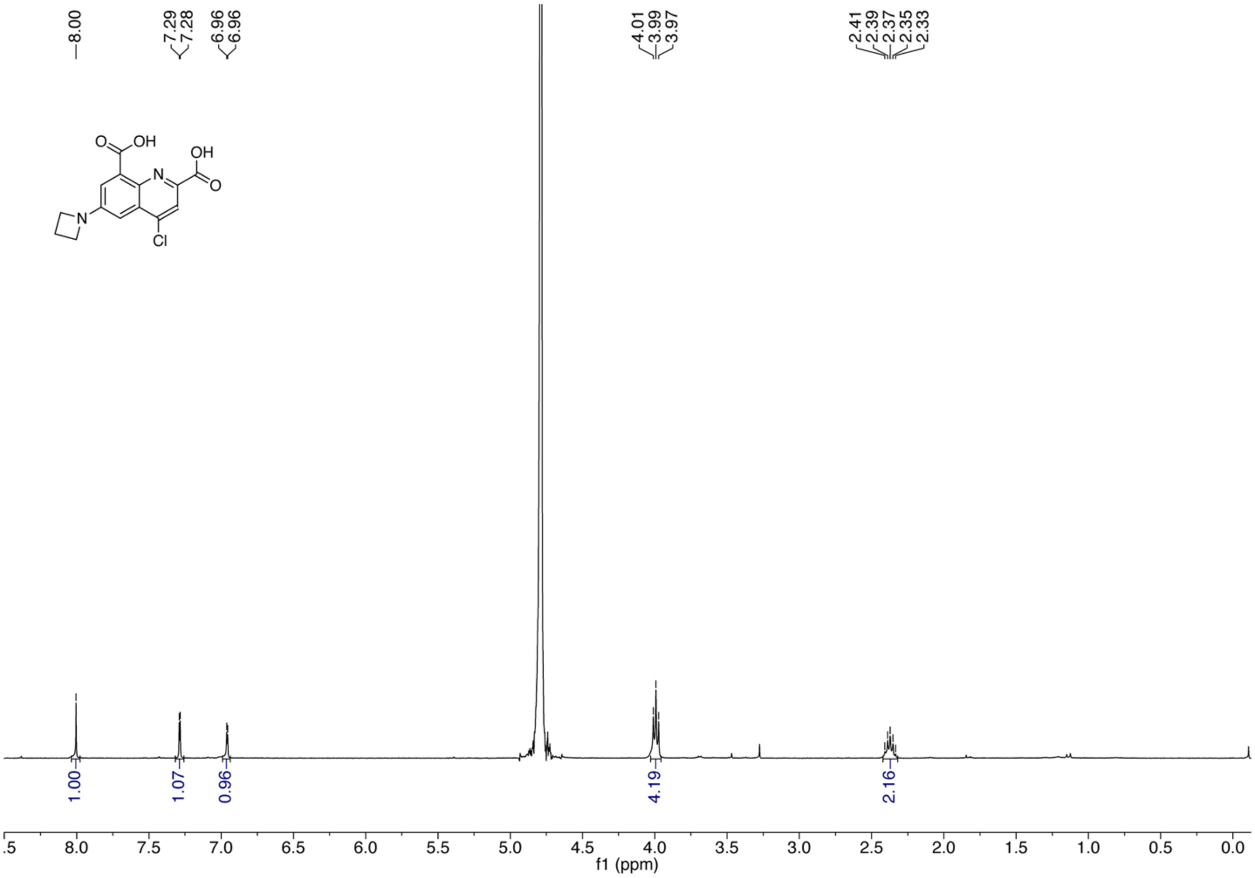

Figure S38. 1H NMR of compound **13** in NaOD.

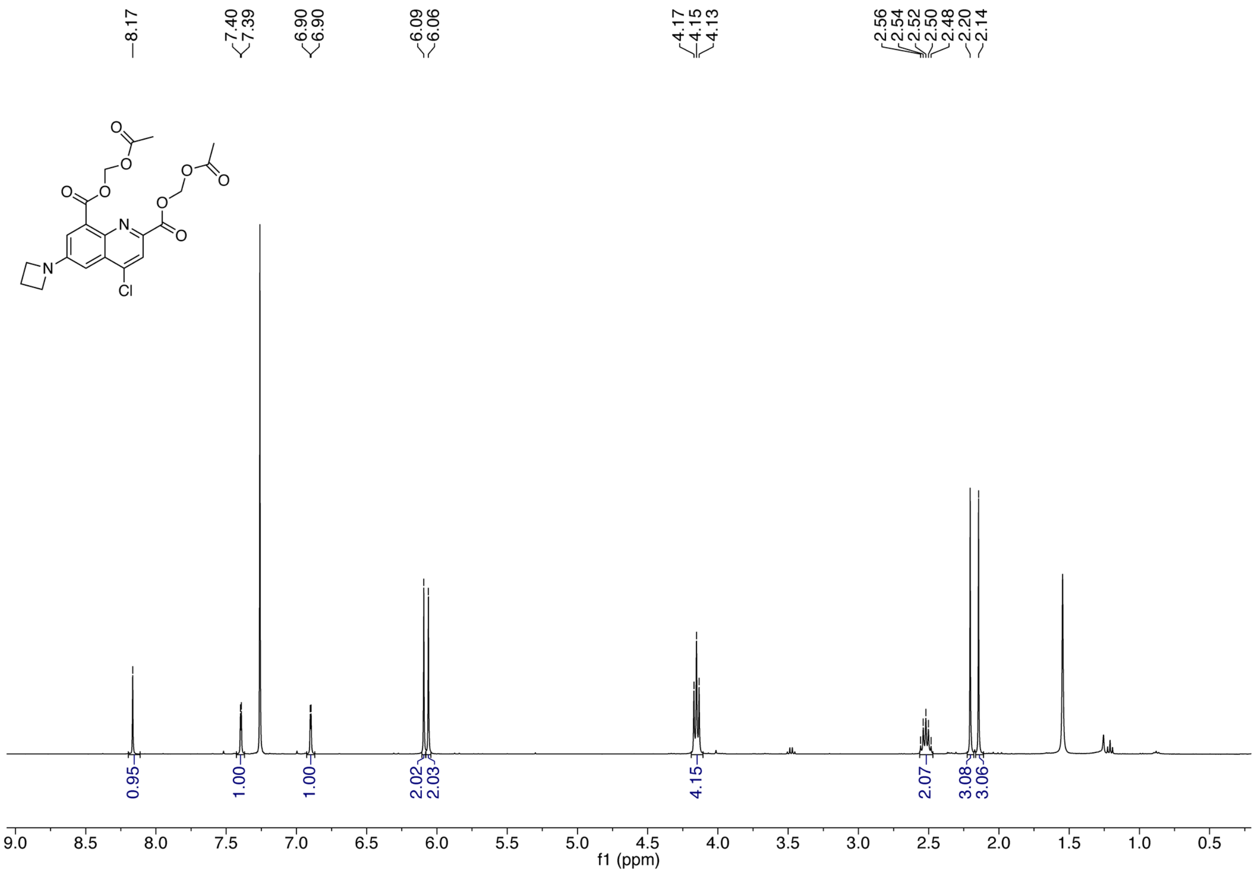

Figure S39. ^{1}H NMR of compound **14** in $CDCl_3$.

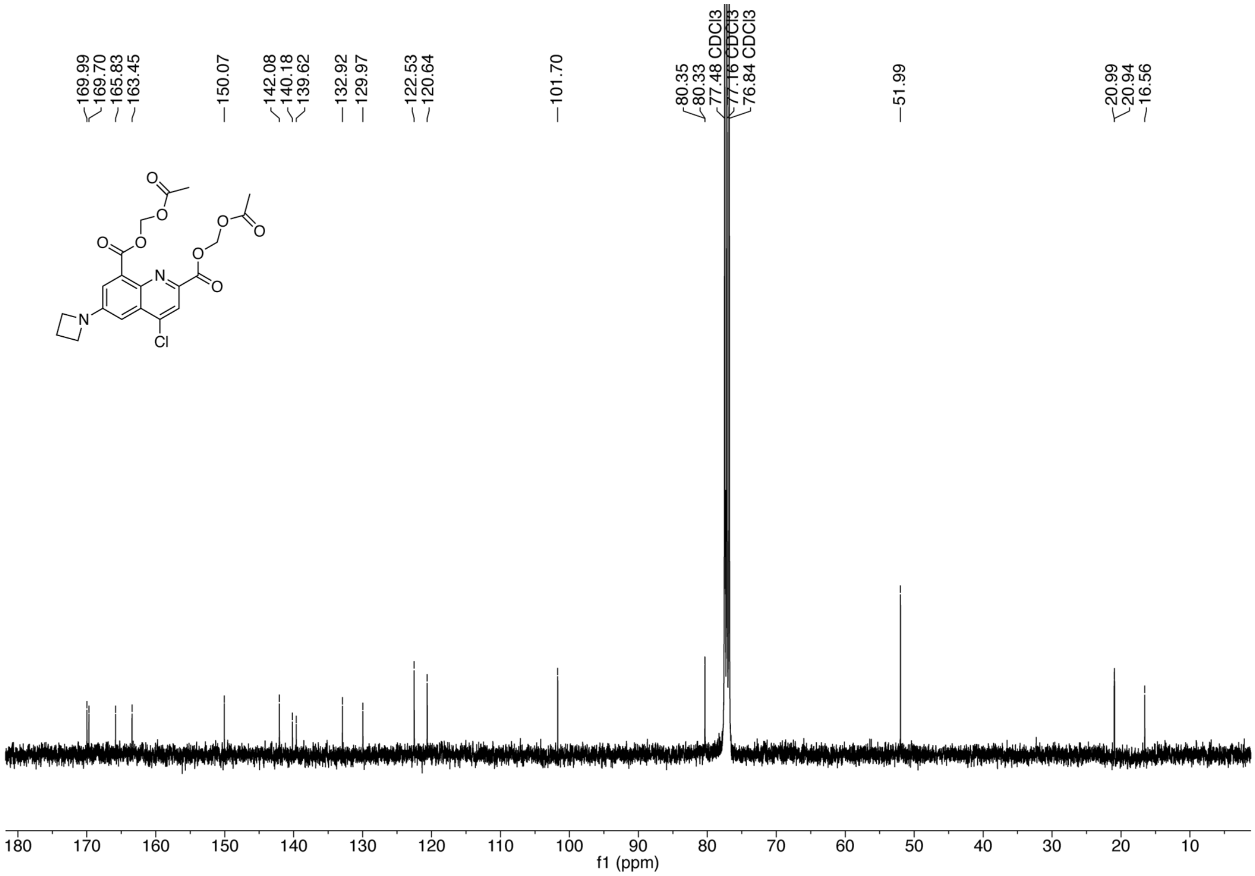

Figure S40. $^{13}C\{^{1}H\}$ NMR of compound **14** in $CDCl_3$

4 References

(1) Grynkiewicz, G.; Tsien, R. Preparation of acyloxymethyl halides from acylals. *Pol. J. Chem.* **1987**, *61* (4-6), 443-447.

(2) Conway, S. J.; Thuring, J. W.; Andreu, S.; Kvinlaug, B. T.; Roderick, H. L.; Bootman, M. D.; Holmes, A. B. The Synthesis of Membrane Permeant Derivatives of myo-Inositol 1,4,5-Trisphosphate. *Aust. J. Chem.* **2006**, *59* (12), 887-893.

(3) Matsui, Y.; Sadhu, K. K.; Mizukami, S.; Kikuchi, K. Highly selective tridentate fluorescent probes for visualizing intracellular Mg^{2+} dynamics without interference from Ca^{2+} fluctuation. *Chem. Commun.* **2017**, *53* (77), 10644-10647.

(4) Chaudhuri, S. K.; Roy, S.; Saha, M.; Bhar, S. Regioselective Aromatic Electrophilic Bromination with Dioxane Dibromide Under Solvent‐Free Conditions. *Synth. Commun.* **2007**, *37* (4), 579-583.

(5) Eaton, D. F. International Union of Pure and Applied Chemistry Organic Chemistry Division Commission on Photochemistry. Reference materials for fluorescence measurement. *J. Photochem. Photobiol. B* **1988**, *2* (4), 523-531.

(6) Grynkiewicz, G.; Poenie, M.; Tsien, R. Y. A new generation of Ca^{2+} indicators with greatly improved fluorescence properties. *J. Biol. Chem.* **1985**, *260* (6), 3440-3450.

(7) Martell, A.; Smith, R. M. *Critical Stability Constants. First Supplement*; Plenum Press, 1982.

(8) *Gaussian 16 Rev. A.03*; Wallingford, CT, 2016.

(9) Zhao, Y.; Truhlar, D. G. The M06 suite of density functionals for main group thermochemistry, thermochemical kinetics, noncovalent interactions, excited states, and transition elements: two new functionals and systematic testing of four M06-class functionals and 12 other functionals. *Theor. Chem. Acc.* **2008**, *120* (1), 215-241.
(10) Jacquemin, D.; Planchat, A.; Adamo, C.; Mennucci, B. TD-DFT Assessment of Functionals for Optical 0–0 Transitions in Solvated Dyes. *J. Chem. Theory Comput.* **2012**, *8* (7), 2359-2372.
(11) Li, R.; Zheng, J.; Truhlar, D. G. Density functional approximations for charge transfer excitations with intermediate spatial overlap. *Phys. Chem. Chem. Phys.* **2010**, *12* (39), 12697-12701.
(12) Leang, S. S.; Zahariev, F.; Gordon, M. S. Benchmarking the performance of time-dependent density functional methods. *J. Chem. Phys.* **2012**, *136* (10), 104101.
(13) Charaf-Eddin, A.; Planchat, A.; Mennucci, B.; Adamo, C.; Jacquemin, D. Choosing a Functional for Computing Absorption and Fluorescence Band Shapes with TD-DFT. *J. Chem. Theory Comput.* **2013**, *9* (6), 2749-2760.
(14) Tomasi, J.; Mennucci, B.; Cammi, R. Quantum Mechanical Continuum Solvation Models. *Chem. Rev.* **2005**, *105* (8), 2999-3094.
(15) Bruno, I. J.; Cole, J. C.; Edgington, P. R.; Kessler, M.; Macrae, C. F.; McCabe, P.; Pearson, J.; Taylor, R. New software for searching the Cambridge Structural Database and visualizing crystal structures. *Acta Crystallogr. B* **2002**, *58* (Pt 3 Pt 1), 389-397.
(16) Schindelin, J.; Arganda-Carreras, I.; Frise, E.; Kaynig, V.; Longair, M.; Pietzsch, T.; Preibisch, S.; Rueden, C.; Saalfeld, S.; Schmid, B.; et al. Fiji: an open-source platform for biological-image analysis. *Nat. Methods* **2012**, *9* (7), 676-682.
(17) Cox, C.; Reeder, J. E.; Robinson, R. D.; Suppes, S. B.; Wheeless, L. L. Comparison of frequency distributions in flow cytometry. *Cytometry* **1988**, *9* (4), 291-298.
(18) *R: A language and environment for statistical computing*; R Foundation for Staistical Computing: Vienna, Austria, 2021. https://www.R-project.org/
